# Global burden of *Klebsiella pneumoniae* infections and antimicrobial resistance in 2019

**DOI:** 10.1186/s12879-025-12120-w

**Published:** 2025-11-21

**Authors:** Hui-Wen Song, Yong Lin, Tu-er Wan, Gui-Hua Yang, Mei-Ting Jiang, Bin-Hong Fu, Jin-Shui Pan

**Affiliations:** 1 Department of Hepatology, The First Affiliated Hospital of Fujian Medical University, No. 20, Chazhong Road, Fuzhou, Fujian 350005 China; 2 Department of Infectious Diseases, Sanming First Hospital Affiliated to Fujian Medical University, Sanming, Fujian China; 3 The School of Clinical Medicine, Fujian Medical University, Fuzhou, Fujian China; 4 Department of Radiology, Sanming First Hospital Affiliated to Fujian Medical University, Sanming, Fujian China; 5 Department of Infectious Diseases, Shaowu Municipal Hospital, No. 10, Ligangdong Road, Shaowu, Fujian 354000 China; 6 Hepatology Research Institute, Fujian Medical University, Fuzhou, Fujian China; 7 Fujian Clinical Research Center for Liver and Intestinal Diseases, Fuzhou, Fujian China

**Keywords:** Mortality, Disability-adjusted life years, Global burden of disease, *Klebsiella pneumoniae*, Antimicrobial resistance

## Abstract

**Purpose:**

To conduct an extensive analysis of the global burden of *Klebsiella pneumoniae* (*K. pneumoniae*) infections and antimicrobial resistance (AMR) using data from 2019, encompassing international, regional, and national perspectives.

**Methods:**

The methodology employed in this study involved collecting data on *K. pneumoniae* infections and AMR from two reputable sources: the Global Burden of Disease Study (GBD) 2019 and the Global Burden of Antimicrobial Resistance 2019 study. The analysis was based on a vast compilation of 471 million records, which informed eight distinct modeling components.

**Results:**

*K. pneumoniae* infections have resulted in approximately 800,000 deaths worldwide, with 80% associated with AMR. Notably, lower respiratory tract infections, bloodstream infections, and intra-abdominal infections were identified as the primary infections caused by *K. pneumoniae*, accounting for nearly 90% of *K. pneumoniae*-related deaths and exhibiting the highest age-standardized mortality rates. South Asia and sub-Saharan Africa showed the greatest incidence of fatalities linked to *K. pneumoniae* infections, with the latter region additionally displaying the highest number and rate of deaths associated with antimicrobial-resistant *Klebsiella pneumoniae* (KP-AMR). The mortality attributed to KP-AMR was predominantly caused by resistance to carbapenems and third-generation cephalosporins, accounting for more than 50% of the reported deaths.

**Conclusions:**

*K. pneumoniae* infections and antimicrobial resistance significantly impact sub-Saharan Africa and South Asia, underscoring an urgent need for targeted interventions.

**Supplementary Information:**

The online version contains supplementary material available at 10.1186/s12879-025-12120-w.

## Introduction

*Klebsiella pneumoniae*, a member of the Enterobacteriaceae family, is implicated in severe clinical conditions such as pneumonia, bloodstream infections, and infections within neonatal and intensive care units [1, 2]. The bacterium poses a substantial challenge to global public health, as evidenced by the escalating issue of antimicrobial resistance (AMR). The World Health Organization (WHO) has identified carbapenemase-resistant *K. pneumoniae* (CRKP) as one of the highest-priority pathogens [3], a concern strongly supported by recent global surveillance data indicating critically high and rising resistance levels, particularly in resource-limited settings [4]. *K. pneumoniae* is further classified into classical *K. pneumoniae* (cKP) and hypervirulent *K. pneumoniae* (hvKP). The hvKP strain is distinguished by its heightened virulence, invasiveness, and pathogenicity, and has the capacity to induce severe invasive infections, including liver and lung abscesses, as well as central nervous system infections [5, 6].

Despite recent research publications addressing the consequences of bacterial infections and drug resistance on disease burden [7–10], studies focusing on the specific role of individual bacteria in disease causation remain scarce. The WHO and the Maternal and Child Epidemiology Estimation have provided global estimates concerning the prevalence of *Streptococcus pneumoniae* and *Haemophilus influenzae* type b infections among children [11]. Furthermore, in 2014, estimates were generated regarding the worldwide incidence of antibiotic-resistant infections caused by *Escherichia coli* and *K. pneumoniae* [12]. Recent reports from the WHO Global Antimicrobial Resistance and Use Surveillance System (GLASS) indicate that resistance to carbapenems in *K. pneumoniae* bloodstream infections has reached 15.1% globally, with alarming annual increases, underscoring the need for updated epidemiological assessments. Despite the comprehensive assessments of the global impact of *K. pneumoniae* infections and AMR, such as the study published in The Lancet estimating the burden of AMR and its effects on mortality [7], there is still a lack of thorough evaluations. Moreover, the emergence of trends in bacterial drug resistance significantly hinders the prioritization of public health initiatives.

Conducting a thorough investigation into the epidemiological characteristics of *K. pneumoniae* and its resistance patterns is crucial to elucidate its role in the global disease burden and inform the development of effective public health strategies. This study seeks to comprehensively analyze the global impact of *K. pneumoniae* infections and AMR in 2019. The analysis encompasses eight distinct infectious syndromes and six combinations of pathogens and antibiotics. To assess the prevalence of infections and AMR due to *K. pneumoniae* at global, regional, and national levels, we employed data from the Global Burden of Disease Study (GBD) 2019.

## Methods

### Data sources

The data regarding the quantity and prevalence of fatalities related to *K. pneumoniae* infections and AMR were obtained from the GBD 2019 (http://ghdx.healthdata.org/gbd-2019) and the Antimicrobial Resistance 2019 studies (https://www.healthdata.org/research-analysis/health-risks-issues/antimicrobial-resistance-amr). The GBD study is an extensive collaborative research dataset encompassing all member countries of the WHO, offering epidemiological insights from 204 territories or countries. This comprehensive dataset includes information on 369 disorders and 87 associated risk factors [13]. To assess the magnitude of AMR, a total of 471 million separate records or isolates were gathered, encompassing a range of factors, including multiple causes of mortality, hospital discharge, minimally invasive tissue sampling, comprehensive analysis of published studies, and microbiology laboratory findings obtained from both domestic and international surveillance systems [7]. These extensive datasets were utilized to identify eight modeling components, which were subsequently used to estimate the burden of *K. pneumoniae* infections and their association with AMR [8].

### International classification of diseases (ICD) codes mapped to the GBD cause list

The appendix accompanying this study provides critical information on the corresponding ICD codes for each disease analyzed.

### Related terms

GBD 2019 is defined by the following terms:

#### Years of life lost (YLLs)

years lost resulting from premature mortality.

#### Years lived with disability (YLDs)

the total number of years an individual lives with a condition that limits their ability to function at total capacity.

#### Disability-adjusted life years (DALYs)

the summation of years of life lost because of YLLs and YLDs.

### Statistical analysis

The study was conducted in two phases. The initial phase encompassed the acquisition of data about infectious syndromes caused by *K. pneumoniae* through three modeling steps, which have been documented in a previous publication [8]. In the first stage, fatalities were identified where the infections contributed to the death. Subsequently, the proportion of fatalities attributable to the illness and its specific contagious condition was computed. Finally, the proportion of deaths attributable to a particular microorganism responsible for the infectious condition was ascertained [8]. The estimation of the burden of KP-AMR in the latter part encompasses five primary elements: the quantification of fatalities impacted by the infections, the proportion of infectious deaths associated with a specific contagious syndrome, the allocation of infectious syndrome deaths attributed to a particular pathogen, the percentage of a specific pathogen exhibiting resistance to a targeted antibiotic, and the supplementary risk of mortality or duration of an infection associated with this resistance. By adhering to these procedures, the assessment of the impact of AMR can be ascertained by considering two hypothetical scenarios: deaths attributed to AMR and fatalities associated with AMR, as previously documented [7]. The final estimations of the ranked values were computed utilizing the 2.5th and 97.5th percentiles from 1000 posterior samples, accompanied by 95% uncertainty intervals (UIs).

In this study, data concerning mortality, YLLs, YLDs, and DALYs associated with *K. pneumoniae* infections and AMR were extracted from the GBD 2019 database. The data were then subjected to retrospective analysis and comparative evaluation across various regions, countries, infection types, and drug-pathogen pairs utilizing GraphPad Prism 9 software. Statistical significance was determined at a threshold of *P* < 0.05 (two-tailed).

## Results

The GBD study identified eight distinct categories of infections globally associated with *K. pneumoniae*. These categories include lower respiratory tract infections (LRIs) and related thoracic infections, bloodstream infections (BSIs), peritoneal and intra-abdominal infections (IAIs), urinary tract infections (UTIs) and pyelonephritis, meningitis and other bacterial infections affecting the central nervous system (CNS), endocarditis and other infections impacting the heart, bacterial skin and subcutaneous inflammation, and infections of the bones, joints, and associated organs.

Based on the study, it was determined that the infections caused by *K. pneumoniae* gave rise to eight distinct infectious syndromes, resulting in an estimated 800,000 fatalities (95% UI: 571,000–1,062,000) across all age groups (Table 1). Additionally, the age-standardized mortality rate (ASMR) for *K. pneumoniae* infectious syndromes in that particular year was calculated to be 10.6 (with a range of 7.7 to 14.2) per 100,000 people (Supplementary Table 1). Among the infectious syndromes caused by *K. pneumoniae*, the most prevalent were LRIs, which resulted in 276,000 (220,000-343,000) deaths; BSIs, which accounted for 265,000 (157,000-416,000) deaths; and IAIs, which caused 158,000 (103,000-234,000) deaths (Table 1). These three syndromes collectively contributed to nearly 90% of *K. pneumoniae* infection-associated fatalities, amounting to a total of 700,000 deaths. Notably, LRIs, BSIs, and IAIs also exhibited the highest ASMRs: LRIs 3.85 (3.07–4.77), BSIs 3.85 (3.07–4.77), and IAIs 1.99 (1.30–2.94) per 100,000 population (Supplementary Table 1).

**Table 1:** Global death counts associated with *K. pneumoniae* infections in GBD regions, by infectious syndrome, 2019

	All infectious syndromes	LRIs and all related infections in the thorax	Bloodstream infections	Peritoneal and intra-abdominal infections	UTIs and pyelonephritis	Meningitis and other bacterial CNS infections	Endocarditis and other cardiac infections	Bacterial infections of the skin and subcutaneous systems	Infections of bones, joints, and related organs
Global	789,804 (570943-1061927)	275,600 (219980-343066)	264,745 (157333-416027)	157,874 (103335-233820)	38,654 (26943-55797)	33,388 (23604-47040)	11,166 (8092-15388)	7004 (1073-25783)	1373 (389-3196)
East Asia	76,889 (49011-117944)	16,603 (11517-23636)	32,447 (17867-54291)	18,947 (11155-30825)	5336 (2525-9674)	1250 (738-2339)	962 (560-1601)	1163 (145-4204)	180 (57-426)
Southeast Asia	65,170 (45778-90378)	21,318 (16544-27445)	20,076 (11591-32439)	17,305 (11311-25126)	3245 (2033-4852)	1563 (1077-2342)	832 (631-1127)	728 (109-2674)	103 (28-249)
Oceania	1937 (1368-2675)	1082 (784-1441)	568 (333-884)	134 (75-229)	37 (20-60)	86 (54-132)	17 (12-23)	12 (1-47)	2 (1-6)
Central Asia	8513 (5811-12063)	2514 (1968-3220)	3064 (1704-5133)	2148 (1342-3210)	529 (362-781)	111 (70-196)	86 (51-138)	53 (6-206)	9 (2-22)
Central Europe	12,831 (8267-19061)	2436 (1830-3299)	5045 (2651-8665)	3567 (2285-5304)	1002 (590-1629)	101 (55-202)	538 (295-893)	127 (16-464)	17 (5-40)
Eastern Europe	24,020 (15575-34649)	4347 (3159-5997)	9366 (4979-15841)	7001 (4529-10137)	2100 (1383-3141)	271 (177-471)	607 (358-1001)	288 (44-1003)	38 (11-91)
High-income Asia Pacific	20,310 (14903-27055)	7193 (5894-8292)	5683 (3148-9316)	4863 (3025-7355)	1686 (1219-2353)	84 (52-156)	600 (336-847)	178 (24-660)	21 (7-48)
Australasia	1825 (1231-2581)	361 (273-476)	663 (354-1107)	481 (308-723)	209 (158-284)	12 (7-25)	64 (40-90)	32 (5-108)	3 (1-7)
Western Europe	43,998 (31144-61058)	9634 (7853-11771)	14,172 (7486-24028)	12,157 (8080-17471)	5051 (3843-6707)	240 (155-433)	2088 (1327-2867)	597 (89-2006)	58 (19-131)
Southern Latin America	8940 (6766-11674)	3337 (2837-3934)	2406 (1306-4058)	1909 (1287-2742)	849 (633-1090)	78 (57-116)	252 (198-313)	100 (17-318)	9 (2-21)
High-income North America	32,880 (22761-46580)	7476 (5641-9960)	11,163 (6034-18733)	8674 (5733-12608)	3671 (2855-4907)	215 (136-397)	1220 (755-1638)	407 (67-1315)	53 (17-122)
Caribbean	5317 (3681-7485)	1783 (1328-2313)	1794 (1045-2830)	1107 (702-1681)	225 (158-324)	264 (161-429)	57 (43-76)	76 (12-257)	10 (3-24)
Andean Latin America	7017 (4916-9634)	2794 (2178-3603)	2216 (1289-3502)	1545 (985-2300)	306 (203-438)	62 (39-106)	52 (38-69)	35 (4-133)	8 (2-18)
Central Latin America	23,424 (15778-33008)	5819 (4238-7794)	8434 (4695-13533)	6838 (4481-9837)	1564 (1091-2239)	247 (163-405)	233 (168-317)	250 (40-871)	37 (10-89)
Tropical Latin America	25,752 (18913-34611)	8921 (7219-11118)	8070 (4605-13205)	5302 (3627-7562)	2424 (1777-3016)	272 (191-408)	449 (336-563)	279 (49-926)	36 (10-84)
North Africa and Middle East	42,628 (28933-61186)	13,749 (10387-17896)	15,469 (8790-24547)	9557 (5879-14514)	1408 (709-2411)	1411 (814-2584)	576 (399-832)	341 (41-1309)	117 (30-277)
South Asia	190,582 (135235-260363)	70,612 (53724-90705)	64,776 (37234-103476)	36,008 (22797-55115)	7762 (5605-10777)	7754 (5534-11281)	1679 (1187-2348)	1544 (163-6097)	446 (119-1060)
Central Sub-Saharan Africa	20,319 (15280-26630)	10,727 (8251-13763)	5685 (3754-8333)	2341 (1399-3761)	138 (79-211)	1207 (808-1786)	101 (66-152)	90 (7-383)	29 (7-71)
Eastern Sub-Saharan Africa	67,792 (52123-87210)	30,834 (24505-38780)	22,277 (14688-32182)	8169 (5224-12110)	462 (248-682)	5365 (3833-7441)	312 (210-466)	283 (25-1147)	89 (25-204)
Southern Sub-Saharan Africa	12,045 (9241-15574)	5610 (4592-6857)	4192 (2727-6118)	1406 (919-2129)	150 (84-239)	473 (349-630)	101 (74-140)	93 (11-360)	19 (6-45)
Western Sub-Saharan Africa	97,672 (74879-125479)	48,476 (38160-60701)	27,191 (17890-39383)	8427 (5068-13079)	499 (316-718)	12,324 (7965-18605)	340 (258-460)	328 (30-1308)	88 (23-209)

95% uncertainty intervals are shown in parantheses. *K. pneumoniae* Klebsiella pneumoniae. LRIs = Lower respiratory infections. UTIs = Urinary tract infections. CNS = central nervous system. GBD = Global Burden of Diseases, Injuries, and Risk Factors Study

The geographic distribution of the eight *K. pneumoniae* infectious syndromes varied considerably across 21 GBD regions. South Asia recorded the highest number of fatalities (191,000 deaths; 135,000–260,000), followed by sub-Saharan Africa and East Asia (Table 1). However, when assessed by age-standardized mortality rates (ASMRs), four sub-Saharan African regions ranked highest, ahead of Oceania and South Asia (Supplementary Table 1). In contrast, Australasia, Oceania, and the Caribbean had the fewest deaths, each under 6,000 (Table 1). Australasia also showed the lowest ASMR, at 3.48 (2.34–4.99) per 100,000 people (Supplementary Table 1).

**Fig. 1:**
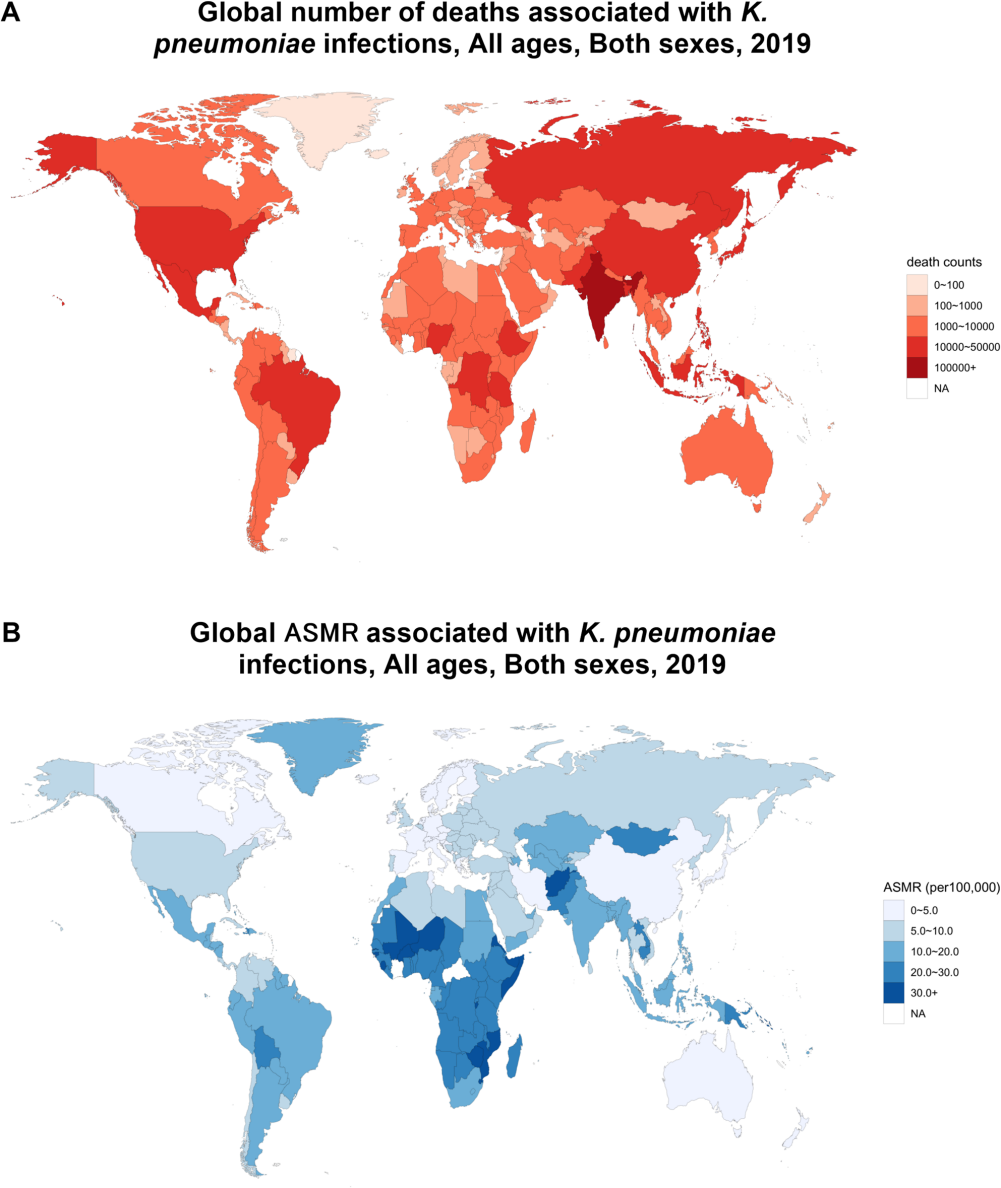
Global number of deaths and age-standardized mortality rates associated with *Klebsiella pneumoniae* infections in 204 countries and territories. (**A**) Deaths associated with *Klebsiella pneumoniae* infections in 204 countries and territories. (**B**) Global age-standardized mortality rates associated with *Klebsiella pneumoniae* infections in 204 countries and territories

At the country level, India had the highest number of deaths associated with *K. pneumoniae* infections (134,000; 94,000–186,000), followed by China (72,000; 45,000–111,000) and Nigeria (46,000; 34,000–59,000) (Fig. 1A). The Central African Republic recorded the highest ASMR (41.01 per 100,000; 29.24–55.90), ahead of Lesotho (40.09; 28.56–55.26) and Guinea-Bissau (36.35; 27.25–48.22). In contrast, Iceland exhibited the lowest ASMR (2.97; 2.04–4.15), followed by Bermuda (3.16; 2.04–4.68) and Switzerland (3.34; 2.30–4.65) (Fig. 1B).

Antimicrobial resistance is a significant and concerning threat, resulting in an estimated 642,000 (465,000-863,000) deaths associated with KP-AMR. Notably, 193,000 deaths were attributed solely to KP-AMR in 2019. The impact of KP-AMR exhibited variability across the subregions of the GBD, with sub-Saharan Africa experiencing the highest level of impact, resulting in 186,000 deaths (144,000-240,000) associated with KP-AMR. South Asia closely followed with 175,000 deaths (124,000-241,000), while Southeast Asia, East Asia, and Oceania reported 104,000 deaths (69,000-151,000) (Table 2). However, there was a shift in the ranking order, with sub-Saharan Africa emerging as the region with the highest mortality rate associated with KP-AMR across all age groups, at 17.29 (13.33–22.17) per 100,000 individuals. Central Europe, Eastern Europe, and Central Asia were closely related at 9.8 (6.49–14.17), and South Asia was closely related at 9.71 (6.88–13.35) (Table 2). Table 2 also presents the corresponding estimates of YLLs, DALYs, and YLDs resulting from KP-AMR.

**Table 2:** Deaths, YLLs, YLDs and dalys resulting from KP-AMR, all ages, 2019

	Associated with resistance				Attributable to resistance			
	Deaths	YLLs	DALYs	YLDs	Deaths	YLLs	DALYs	YLDs
**Counts, thousand**								
Sub-Saharan Africa	186 (144-240)	11,820 (9067-15250)	11,851 (9086-15277)	31 (20-47)	51 (34-72)	3227 (2154-4613)	3235 (2160-4622)	8 (5-13)
South Asia	175 (124-241)	7722 (5560-10414)	7768 (5589-10490)	46 (29-72)	62 (43-88)	2696 (1907-3738)	2710 (1919-3751)	14 (8-22)
Southeast Asia, East Asia, and Oceania	104 (69-151)	2940 (2022-4171)	2954 (2033-4188)	14 (8-22)	30 (18-47)	862 (530-1309)	865 (533-1313)	4 (2-6)
High-income	50 (36-69)	840 (585-1188)	846 (589-1197)	7 (4-10)	12 (8-18)	210 (134-317)	211 (135-318)	2 (1-3)
Latin America and Caribbean	48 (34-66)	1412 (997-1934)	1419 (1003-1943)	8 (5-12)	14 (9-19)	394 (261-572)	396 (263-574)	2 (1-3)
Central Europe, Eastern Europe, and Central Asia	41 (27-59)	1063 (722-1510)	1069 (726-1518)	6 (4-9)	13 (8-19)	325 (208-482)	327 (210-484)	2 (1-3)
North Africa and Middle East	37 (25-53)	1514 (1027-2152)	1521 (1031-2164)	7 (4-11)	11 (7-17)	452 (280-672)	454 (282-674)	2 (1-3)
Global	642 (465-863)	27,315 (20187-35908)	27,433 (20260-36055)	118 (75-177)	193 (130-272)	8166 (5528-11381)	8199 (5554-11412)	33 (20-51)
**Rates**,** per100 000**								
Sub-Saharan Africa	17.29 (13.33-22.17)	1096.26 (840.9-1414.35)	1099.11 (842.66-1416.86)	2.86 (1.83-4.37)	4.71 (3.13-6.66)	299.25 (199.76-427.87)	300.02 (200.32-428.64)	0.77 (0.48-1.2)
Central Europe, Eastern Europe, and Central Asia	9.8 (6.49-14.17)	254.51 (172.79-361.38)	255.88 (173.87-363.44)	1.37 (0.87-2.1)	3.03 (1.93-4.53)	77.82 (49.88-115.49)	78.2 (50.22-115.95)	0.38 (0.23-0.59)
South Asia	9.71 (6.88-13.35)	427.75 (308.01-576.88)	430.31 (309.58-581.09)	2.57 (1.6-4)	3.42 (2.37-4.87)	149.36 (105.66-207.04)	150.11 (106.28-207.81)	0.75 (0.45-1.21)
Latin America and Caribbean	8.24 (5.88-11.27)	241.54 (170.54-331)	242.89 (171.66-332.51)	1.35 (0.86-2.04)	2.31 (1.53-3.3)	67.35 (44.66-97.95)	67.71 (44.96-98.24)	0.35 (0.22-0.56)
North Africa and Middle East	6.1 (4.12-8.77)	248.7 (168.68-353.59)	249.85 (169.34-355.45)	1.15 (0.73-1.78)	1.86 (1.17-2.78)	74.23 (46.05-110.33)	74.55 (46.25-110.74)	0.31 (0.19-0.5)
Southeast Asia, East Asia, and Oceania	4.8 (3.19-6.99)	136.17 (93.62-193.16)	136.8 (94.15-193.97)	0.63 (0.38-1)	1.39 (0.84-2.17)	39.9 (24.55-60.63)	40.06 (24.69-60.8)	0.17 (0.1-0.27)
High-income	4.62 (3.29-6.39)	77.46 (53.92-109.62)	78.06 (54.32-110.45)	0.61 (0.4-0.9)	1.15 (0.74-1.69)	19.33 (12.35-29.21)	19.48 (12.48-29.36)	0.15 (0.09-0.23)
Global	8.29 (6.01-11.16)	353.02 (260.9-464.08)	354.55 (261.83-465.98)	1.52 (0.98-2.29)	2.49 (1.68-3.52)	105.54 (71.44-147.09)	105.96 (71.78-147.49)	0.42 (0.26-0.66)

95% uncertainty intervals are given in parentheses. DALYs=disability-adjusted life-years; KP-AMR=antimicrobial-resistant *Klebsiella pneumoniae*; YLDs=years lived with disability; YLLs=years of life lost

**Fig. 2:**
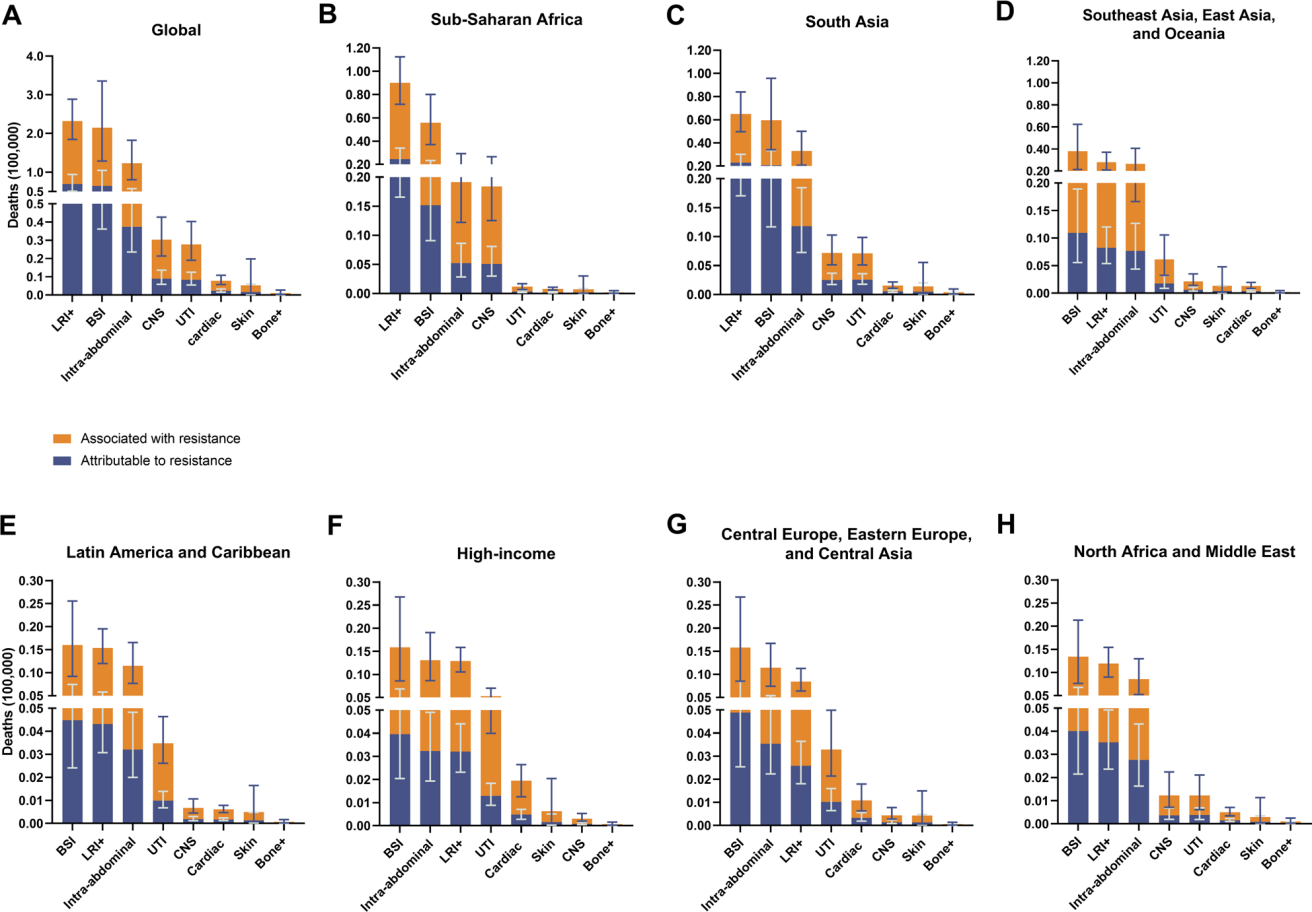
Global and regional mortality associated with and attributable to antimicrobial-resistant *Klebsiella pneumoniae*, by infectious syndrome, 2019. (**A**) Global mortality. (**B**) Mortality in sub-Saharan Africa. (**C**) Mortality in South Asia. (**D**) Mortality in Southeast Asia, East Asia, and Oceania. (**E**) Mortality in Latin America and the Caribbean. (**F**) Mortality in high-income regions. (**G**) Mortality in Central Europe, Eastern Europe, and Central Asia. (**H**) Mortality in North Africa and the Middle East

**Fig. 3:**
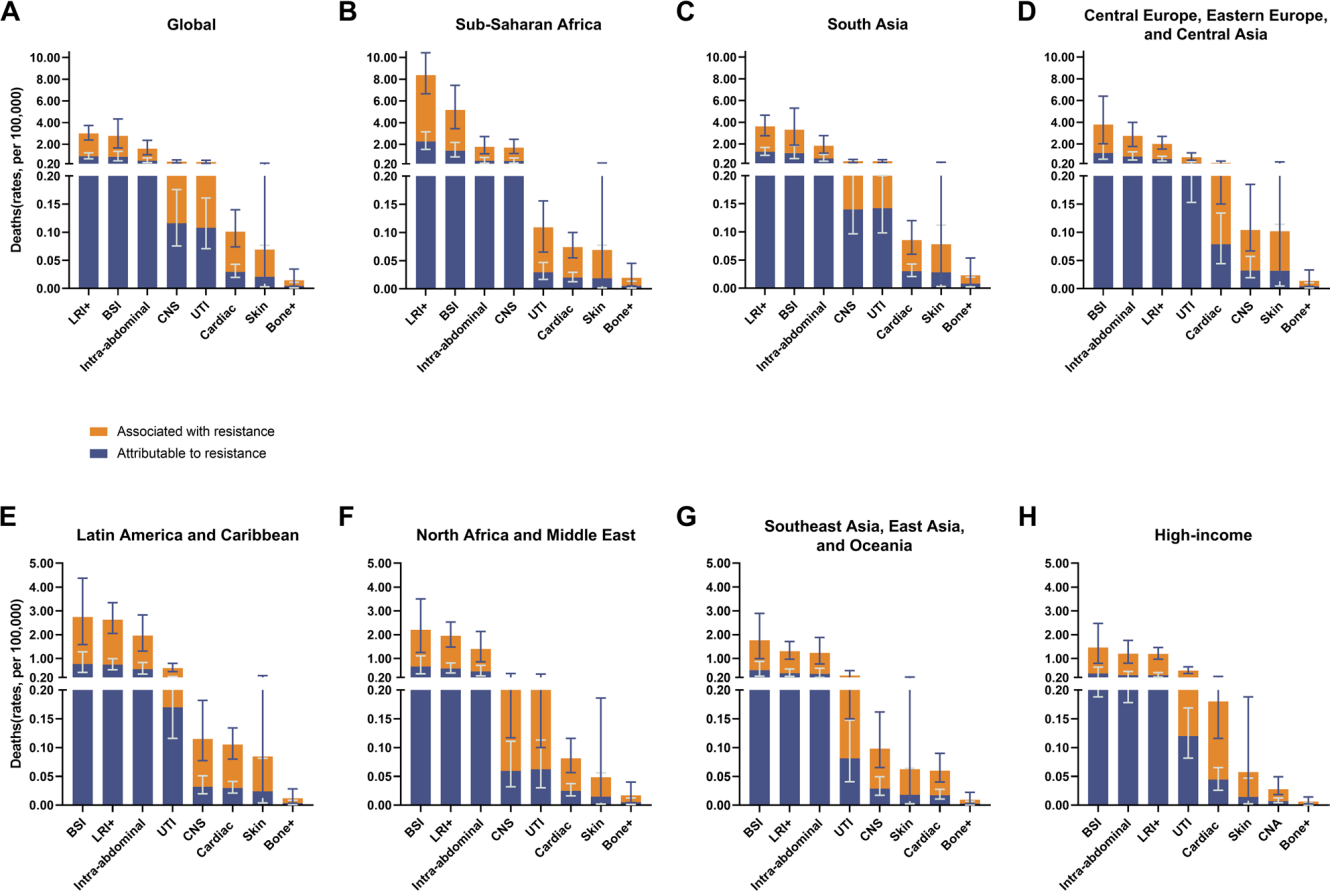
Global and regional mortality rates associated with and attributable to antimicrobial-resistant *K. pneumoniae*, all ages, by infectious syndrome, 2019. (**A**) Global mortality rates. (**B**) Mortality rates in sub-Saharan Africa. (**C**) Mortality rates in South Asia. (**D**) Mortality rates in Central Europe, Eastern Europe, and Central Asia. (**E**) Mortality rates in Latin America and the Caribbean. (**F**) Mortality rates in North Africa and the Middle. (**G**) Mortality rates in Southeast Asia, East Asia, and Oceania. (**H**) Mortality rates in high-income regions

**Fig. 4:**
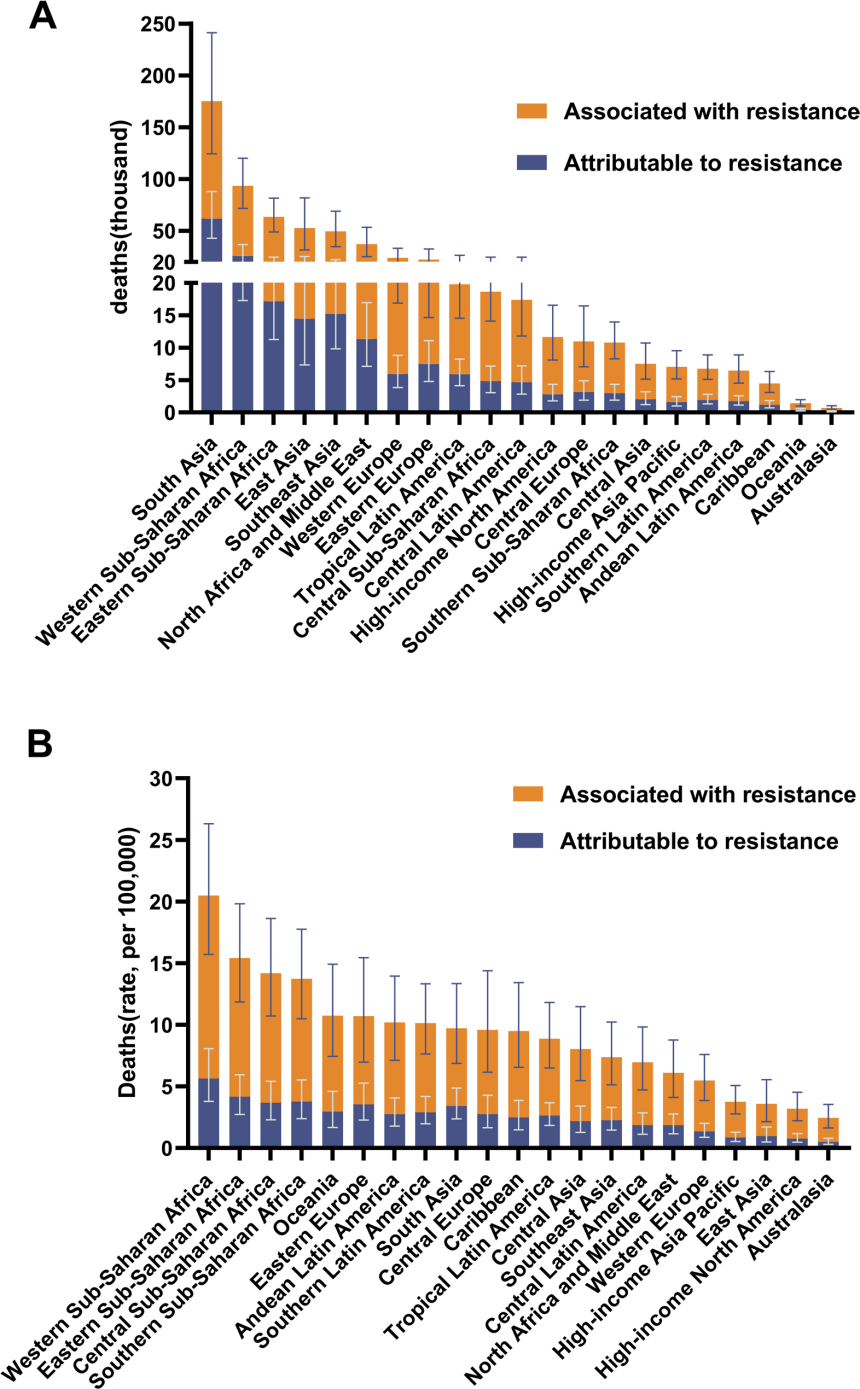
Mortality (numbers and all-age rates) associated with and attributable to antimicrobial-resistant *K. pneumoniae* (KP-AMR) in 21 Global Burden of Disease regions, 2019. (**A**) Deaths linked to KP-AMR. (**B**) All-age mortality rates linked to KP-AMR

LRIs, BSIs, and IAIs accounted for the majority of the global burden associated with KP-AMR across GBD super regions (Figs. 2 and 3). LRIs were the leading cause of AMR-related deaths in the *K. pneumoniae* infectious syndromes, except in sub-Saharan Africa and South Asia, where BSIs were the primary cause in all the other five super regions (Figs. 2 and 3). Furthermore, among the 21 GBD regions, South Asia had the highest number of deaths associated with KP-AMR, with 175,000 (124,000-241,000) fatalities, while sub-Saharan Africa had the highest mortality rate across all age groups (Fig. 4). Moreover, South Asia has emerged as the predominant region in terms of fatalities associated with KP-AMR across all eight infectious syndromes (Supplementary Fig. 1, Supplementary Fig. 2).

According to the GBD 2019 study, carbapenem resistance resulted in more than 55,000 deaths, while third-generation cephalosporins (3GCs) caused 50,000 fatalities, and fluoroquinolones led to 29,000 deaths. The fatalities resulting from infectious syndromes attributable to KP-AMR exhibited geographical variation based on the combination of pathogens and drugs (Supplementary Table 2). South Asia consistently maintained the highest ranking in terms of mortality attributable to KP-AMR, encompassing all six combinations of pathogens and drugs and accounting for more than 50% of the deaths attributable to carbapenem resistance. This percentage was nearly seven times greater than that of Southeast Asia, which secured the second position (Supplementary Table 2). The corresponding mortality rates for all age groups are presented in Supplementary Table 3.

## Discussion

Our study aimed to examine the global implications of *K. pneumoniae* infections and AMR, with a focus on regional disparities and six specific pathogen-drug combinations in 2019. The outcomes of our investigation are as follows: (1) *K. pneumoniae* infections resulted in 800,000 fatalities globally, with 80% associated with AMR. (2) LRIs, BSIs, and IAIs accounted for nearly 90% of the deaths associated with *K. pneumoniae* infections and were among the top three causes of KP-AMR-related mortality. (3) The prevalence of *K. pneumoniae* infections and AMR varied significantly across countries and regions. (4) The highest fatality rates attributed to *K. pneumoniae* infections were observed in South Asia and sub-Saharan Africa, with the latter region experiencing the greatest number of deaths associated with AMR. (5) In the case of all six pathogen‒drug combinations, resistance to carbapenems and 3GCs contributed to more than 50% of the deaths caused by KP-AMR.

In 2019, the burden of eight infectious syndromes caused by AMR was significantly impacted by *K. pneumoniae*. These infectious syndromes include LRIs, BSIs, IAIs, UTIs, CNS infections, endocarditis, and skin and bone infections. Strikingly, LRIs, BSIs, and IAIs collectively accounted for 90% fatalities associated with *K. pneumoniae*, underscoring their clinical priority. *Klebsiella* species are commonly found in healthy individuals’ intestinal and respiratory tracts but become opportunistic pathogens in immunocompromised individuals or during invasive medical procedures [14]. These bacteria can access the bloodstream through multiple pathways, including the respiratory and urinary tracts, intestines, abdominal cavities, intravenous injections, and the umbilical cord in neonates, making bloodstream infections the second most prevalent manifestation despite potential diagnostic biases. Regarding urinary tract infections, while *K. pneumoniae* accounts for 5–15% of all UTIs (substantially less than E. coli) [15], this translates to a significant disease burden given the high overall incidence of UTIs globally [16]. Clinically, *K. pneumoniae*-associated UTIs show a strong predilection for patients with structural urological abnormalities (prostatic hyperplasia, urethral strictures), indwelling catheters, or immunocompromised status [17–19]. However, two distinct epidemiological patterns emerge: (1) in resource-limited settings, empirical UTI treatment without culture confirmation may underestimate *Klebsiella* prevalence in urine isolates [20]; (2) in critically ill hospitalized patients, blood cultures are more systematically obtained than urine cultures, potentially inflating the relative proportion of bloodstream infection reports [21]. The diagnostic stewardship paradox – wherein blood cultures are prioritized in severe cases while urine cultures are omitted in empirical UTI management – creates artificial disparities in reported infection sites. Multidisciplinary antimicrobial stewardship programs that standardize culture collection protocols across body sites could reveal more accurate epidemiological patterns [22].

The incidence of *K. pneumoniae* infections and KP-AMR varies considerably across different regions worldwide. Low- and middle-income areas, such as sub-Saharan Africa and South Asia, bear the brunt of *K. pneumoniae* infections and KP-AMR compared to high-income areas. In recent studies, it has been reported that in low- and middle-income countries (LMICs) where AMR is on the rise, *K. pneumoniae* has been identified as the primary contributing factor to neonatal sepsis and mortality in intensive care units (ICU) [23, 24]. The significant burden of KP-AMR in sub-Saharan Africa and South Asia can be attributed to several factors. These include the increased use of antimicrobial agents, the prevalence of unnecessary self-medication [25, 26], inadequate public health systems, limited access to effective antimicrobial agents [27], poor hygiene practices [28], and limited healthcare-associated infection prevention programs. However, there is a lack of sufficient data on AMR in LMICs due to inadequate surveillance, absence of standardized laboratory facilities, shortage of skilled personnel, and inadequate data management [29].

*K. pneumoniae* exhibits resistance through several mechanisms: (1) production of specific enzymes capable of hydrolyzing β-lactams, with the most clinically relevant being extended-spectrum β-lactamases (ESBLs) such as *Cefotaximases*, and *Carbapenemases* including *K. pneumoniae Carbapenemase* (KPC), *oxacillinase-48*-Like (OXA-48), and *New Delhi metallo-β-lactamase* (NDM); (2) changes in the outer membrane that blocks antibiotic entry [30]; (3) increased capsular polysaccharide synthesis for protection; (4) overexpression of efflux pumps to expel antibiotics; and (5) plasmid-mediated horizontal transfer of resistance genes, which disseminates determinants such as *bla*_CTX−M_ (for ESBL production), *bla*_KPC_ (for carbapenem resistance), and the mobile colistin resistance gene *mcr*. Additional chromosomal mutations (e.g., in *pmrB*, *crrB*, or *mgrB*) may further contribute to polymyxin resistance [31]. AMR is a major global health issue that increases mortality rates by making standard treatments less effective, especially in ICUs. The rise of resistant bacteria limits treatment options, prolongs illness, and contributes to higher mortality [32]. Particularly concerning are the resistances to carbapenems and third-generation cephalosporins (3GCs), which are often last-resort treatments for severe infections caused by gram-negative bacteria. Carbapenem-resistant *Enterobacteriaceae* (CRE) can lead to treatment failures and higher mortality rates, as seen in *Klebsiella* spp. bacteremia [33].

In this study, the majority of deaths caused by KP-AMR can be attributed to resistance to carbapenems and 3GCs. In LMICs, more than one-third of hospital-acquired *K. pneumoniae* infections exhibited resistance to carbapenems, while nearly 80% were resistant to 3GCs [34]. This resistance to 3GCs is linked to higher in-hospital mortality rates, increased healthcare costs, longer hospital stays, and a reduced likelihood of being discharged alive [35, 36]. The evolutionary trajectory of carbapenem resistance reveals three distinct epidemiological phases: (1) localized emergence of KPC-producing clones in US hospitals during the 1990s; (2) intercontinental transfer of *bla*_OXA−48_ via healthcare networks in Mediterranean countries post-2001; and (3) rapid global dissemination of *bla*_NDM_ through medical tourism routes since 2008 [37]. This triphasic expansion has resulted in CRE being documented across all 21 GBD regions, with the annual incidence rising from 0.6 to 1.1 per 100,000 population between 2015 and 2021 [38]. Regional surveillance data demonstrate heterogeneous distribution patterns: CRKP prevalence in China fluctuated between 0.9% (2007) and 19.7% (2020) [39, 40], while US cases declined from 2016 to 2020 before rebounding to pre-pandemic levels by 2022 [41]. Managing these infections requires a stratified approach: (1) Initial empirical therapy with polymyxins or novel tetracycline derivatives (e.g., eravacycline) guided by clinical urgency and drug pharmacokinetics [42]; (2) Targeted combination regimens (e.g., ceftazidime-avibactam + aztreonam for NDM producers) tailored to infection site, severity, and local resistance profiles [42, 43]; (3) Dose optimization using therapeutic drug monitoring to balance efficacy and toxicity.

This study is constrained by two significant limitations. Firstly, inadequate surveillance systems in LMICs have resulted in insufficient data, particularly in sub-Saharan Africa and South Asia. This has caused the emergence of inaccurate information and selection bias. Secondly, the diverse criteria used by various nations to distinguish between drug resistance and susceptibility may lead to discrepancies in classifications.

## Conclusion

In 2019, *K. pneumoniae* infections caused approximately 800,000 global deaths, with 80% of these cases associated with AMR. Lower respiratory, bloodstream, and intra-abdominal infections constituted the majority of this burden, which disproportionately affected sub-Saharan Africa and South Asia, highlighting an urgent need for targeted interventions.

## Supplementary Information

Below is the link to the electronic supplementary material.

Supplementary Material 1

Supplementary Material 2

## Data Availability

Data is provided within the supplementary information files.
