# Supplementary material for: Global burden of Klebsiella pneumoniae infections and antimicrobial resistance in 2019
Source: BMC Infect Dis. 2025 Nov 21;25:1773. doi: 10.1186/s12879-025-12120-w (PMC12751905; doi:10.1186/s12879-025-12120-w)
Supplement: Supplementary file 1 — Supplementary Material 1 [file 12879_2025_12120_MOESM1_ESM.pdf]

Figure S1 Deaths associated with and attributable to antimicrobial-resistant *K. pneumoniae* (KP-AMR) in 21 Global Burden of Disease regions caused by infectious syndromes in 2019

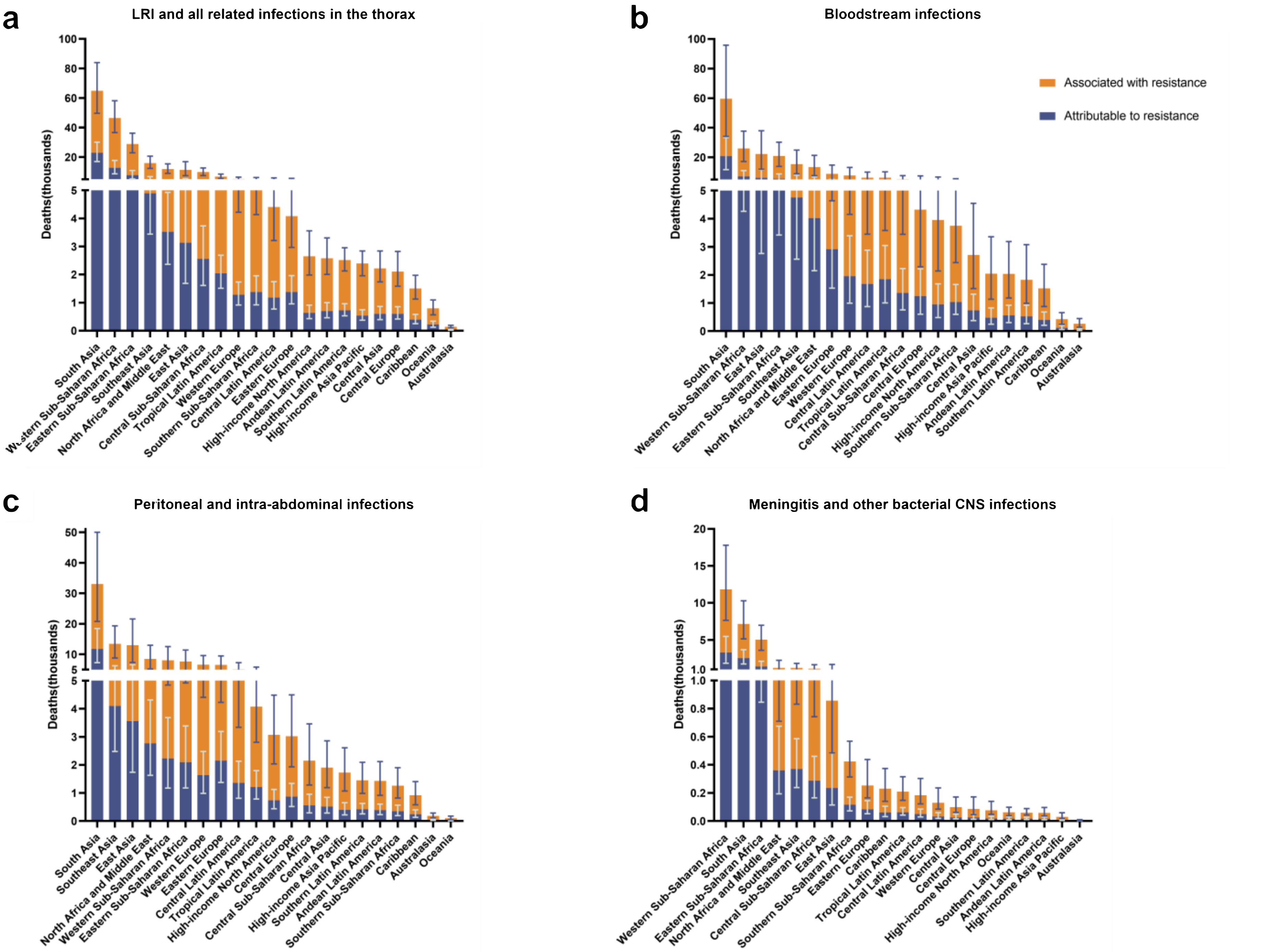

(continued from the previous page)

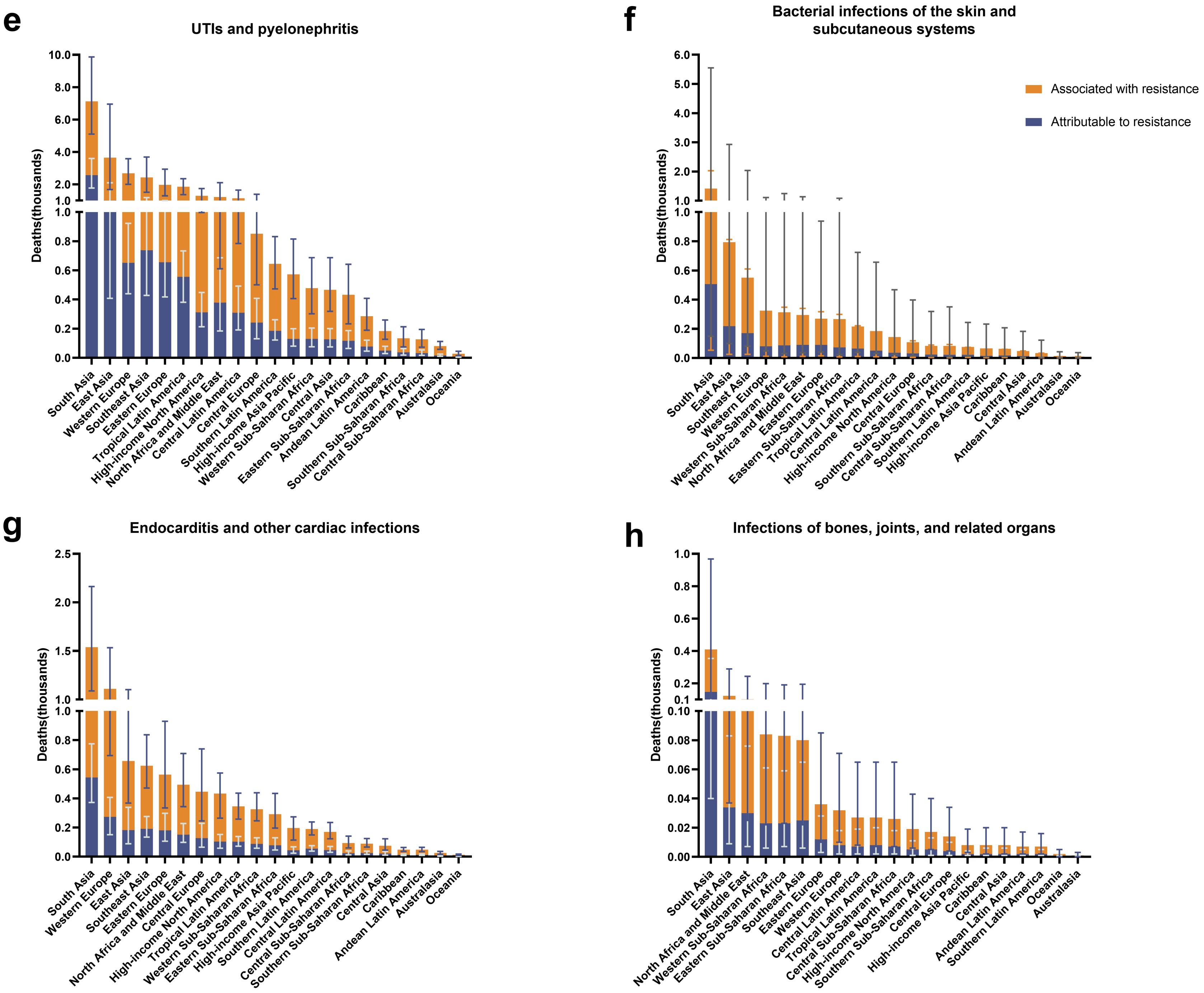

Figure S2 Mortality rates at all ages linked to antimicrobial-resistant *K. pneumoniae* (KP-AMR) in 21 Global Burden of Disease regions, stratified by infectious syndrome in 2019

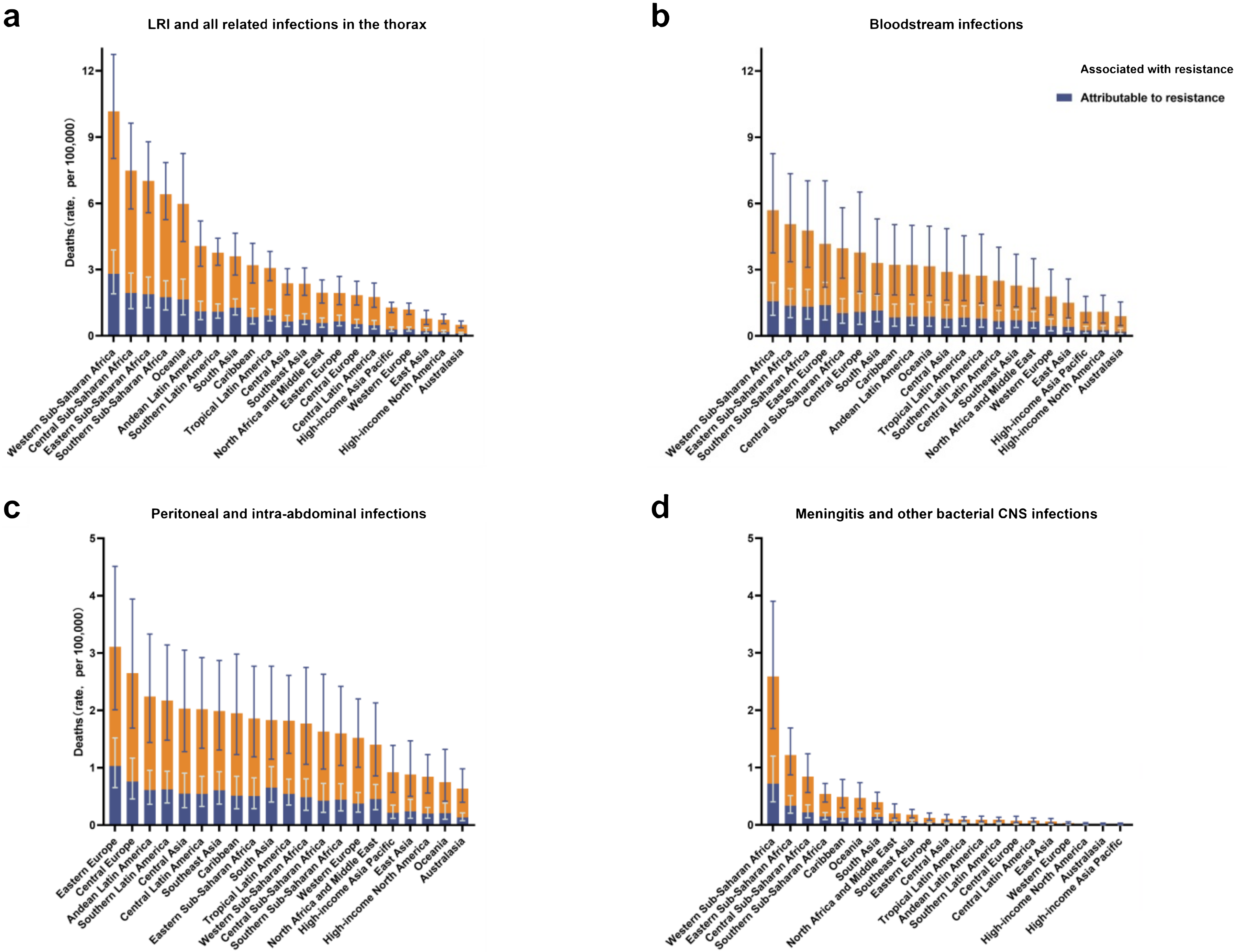

(continued from the previous page)

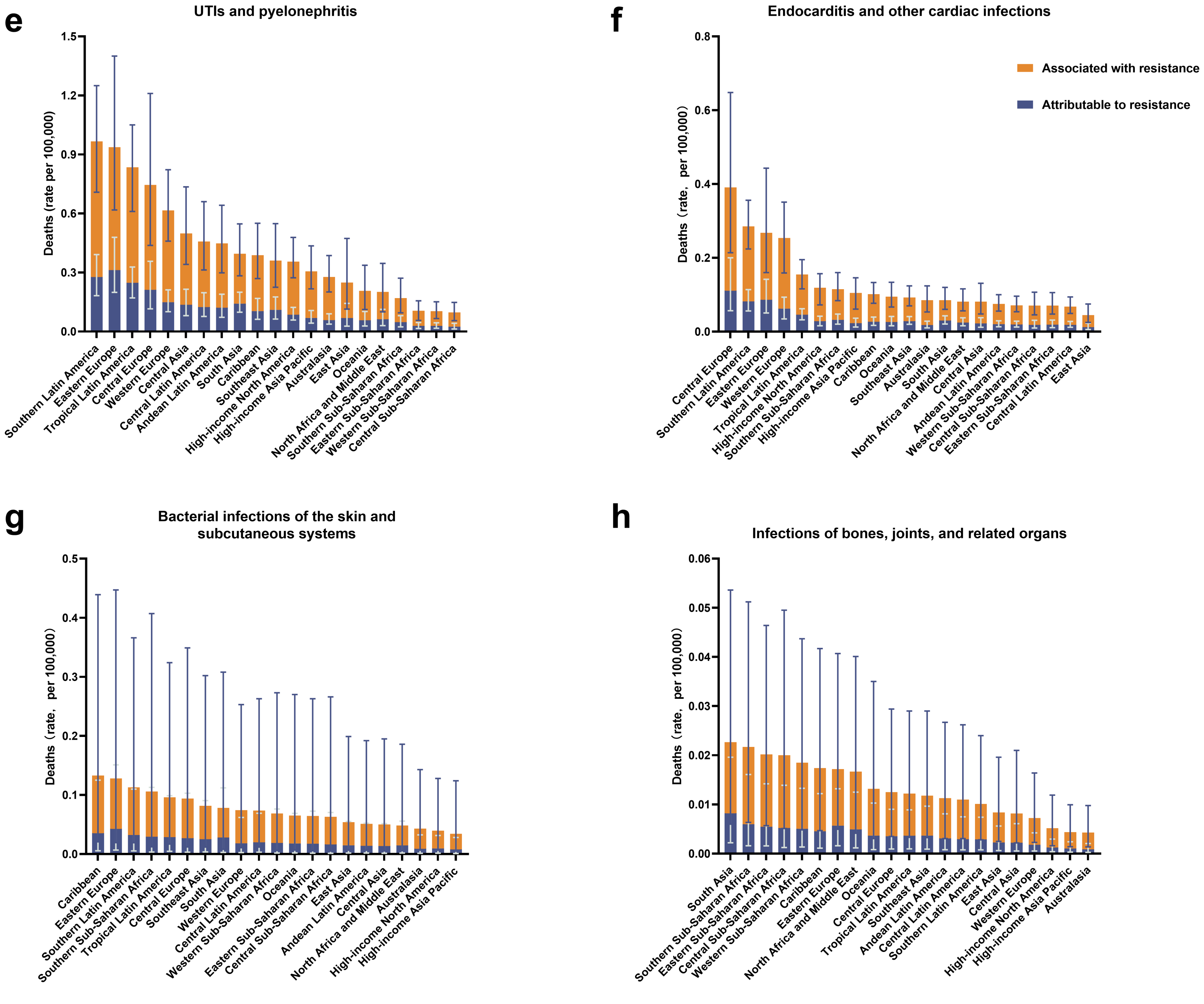

Table S1 Age-standardised mortality rates associated with KP infection in GBD regions, by infectious syndrome, 2019

	All 8 infectious syndromes	LRIs and all related infections in the thorax	Bloodstream infections	Peritoneal and intra-abdominal infections	UTIs and pyelonephritis	Meningitis and other bacterial CNS infections	Endocarditis and other cardiac infections	Bacterial infections of the skin and subcutaneous systems	Infections of bones, joints, and related organs
Global	10.60 (7.69-14.20)	3.85 (3.07-4.77)	3.54 (2.12-5.53)	1.99 (1.30-2.94)	0.51 (0.36-0.73)	0.46 (0.33-0.65)	0.15 (0.11-0.20)	0.09 (0.01-0.33)	0.02 (0.01-0.04)
East Asia	4.37 (2.82-6.63)	1.08 (0.77-1.50)	1.78 (0.98-2.99)	1.00 (0.59-1.63)	0.30 (0.15-0.55)	0.08 (0.05-0.14)	0.05 (0.03-0.09)	0.06 (0.01-0.23)	0.01 (0.00-0.02)
Southeast Asia	12.44 (8.82-17.06)	4.22 (3.28-5.43)	3.80 (2.17-6.17)	3.20 (2.10-4.61)	0.63 (0.39-0.96)	0.26 (0.18-0.39)	0.15 (0.12-0.21)	0.14 (0.02-0.53)	0.02 (0.01-0.05)
Oceania	21.16 (14.54-29.62)	10.46 (7.36-14.32)	6.88 (3.68-11.42)	2.08 (1.19-3.46)	0.69 (0.38-1.12)	0.56 (0.36-0.85)	0.26 (0.18-0.35)	0.20 (0.02-0.85)	0.04 (0.01-0.10)
Central Asia	12.30 (8.29-17.66)	3.43 (2.64-4.51)	4.66 (2.50-7.93)	3.02 (1.90-4.53)	0.83 (0.54-1.25)	0.12 (0.08-0.22)	0.13 (0.08-0.22)	0.09 (0.01-0.34)	0.01 (0.00-0.03)
Central Europe	6.19 (3.95-9.25)	1.21 (0.91-1.62)	2.43 (1.27-4.18)	1.71 (1.09-2.56)	0.45 (0.27-0.74)	0.07 (0.04-0.13)	0.25 (0.14-0.42)	0.06 (0.01-0.21)	0.01 (0.00-0.02)
Eastern Europe	7.47 (4.84-10.80)	1.40 (1.04-1.90)	2.92 (1.58-4.90)	2.14 (1.36-3.13)	0.61 (0.41-0.92)	0.11 (0.07-0.19)	0.19 (0.12-0.31)	0.09 (0.01-0.29)	0.01 (0.00-0.03)
High-income Asia Pacific	3.84 (2.78-5.22)	1.20 (1.00-1.39)	1.23 (0.70-1.96)	0.96 (0.60-1.46)	0.28 (0.20-0.40)	0.02 (0.01-0.04)	0.11 (0.06-0.15)	0.03 (0.01-0.12)	0.00 (0.00-0.01)
Australasia	3.48 (2.34-4.99)	0.65 (0.49-0.88)	1.32 (0.71-2.18)	0.92 (0.58-1.38)	0.37 (0.28-0.51)	0.04 (0.02-0.07)	0.12 (0.08-0.17)	0.06 (0.01-0.19)	0.01 (0.00-0.01)
Western Europe	4.36 (3.04-6.13)	0.87 (0.70-1.09)	1.52 (0.81-2.54)	1.22 (0.81-1.77)	0.44 (0.33-0.60)	0.04 (0.02-0.07)	0.20 (0.13-0.27)	0.06 (0.01-0.19)	0.01 (0.00-0.01)
Southern Latin America	10.78 (8.13-14.11)	4.00 (3.39-4.72)	2.99 (1.65-4.99)	2.27 (1.52-3.27)	0.99 (0.73-1.27)	0.11 (0.08-0.16)	0.30 (0.24-0.37)	0.12 (0.02-0.37)	0.01 (0.00-0.03)
High-income North America	5.16 (3.53-7.35)	1.13 (0.84-1.52)	1.82 (0.99-3.03)	1.38 (0.90-2.01)	0.53 (0.41-0.71)	0.05 (0.03-0.08)	0.19 (0.12-0.25)	0.06 (0.01-0.20)	0.01 (0.00-0.02)
Caribbean	11.10 (7.71-15.55)	3.84 (2.86-4.96)	3.77 (2.24-5.86)	2.16 (1.37-3.28)	0.44 (0.30-0.63)	0.62 (0.38-1.00)	0.11 (0.09-0.15)	0.15 (0.02-0.50)	0.02 (0.01-0.05)
Andean Latin America	12.67 (8.93-17.40)	5.09 (3.98-6.52)	3.92 (2.25-6.21)	2.82 (1.81-4.20)	0.58 (0.38-0.82)	0.10 (0.06-0.18)	0.09 (0.07-0.12)	0.07 (0.01-0.24)	0.01 (0.00-0.03)
Central Latin America	10.30 (6.97-14.47)	2.60 (1.89-3.47)	3.72 (2.07-5.98)	2.96 (1.94-4.25)	0.69 (0.48-0.99)	0.10 (0.07-0.17)	0.10 (0.07-0.14)	0.11 (0.02-0.38)	0.02 (0.00-0.04)
Tropical Latin America	11.62 (8.58-15.55)	4.06 (3.29-5.06)	3.76 (2.19-6.09)	2.26 (1.55-3.22)	1.08 (0.79-1.34)	0.13 (0.09-0.19)	0.19 (0.14-0.24)	0.12 (0.02-0.40)	0.02 (0.00-0.04)
North Africa and Middle East	10.34 (7.02-14.93)	3.14 (2.38-4.10)	3.73 (2.09-6.04)	2.54 (1.60-3.85)	0.40 (0.21-0.68)	0.26 (0.15-0.48)	0.14 (0.10-0.21)	0.09 (0.01-0.36)	0.03 (0.01-0.07)
South Asia	14.50 (10.17-20.04)	5.27 (3.97-6.88)	5.00 (2.81-8.20)	2.78 (1.77-4.21)	0.65 (0.46-0.91)	0.49 (0.35-0.72)	0.14 (0.10-0.19)	0.13 (0.01-0.54)	0.04 (0.01-0.09)
Central Sub-Saharan Africa	27.40 (20.22-36.7)	13.67 (10.34-17.61)	7.03 (4.05-11.38)	4.83 (2.98-7.45)	0.32 (0.19-0.49)	1.06 (0.72-1.53)	0.20 (0.13-0.31)	0.22 (0.02-1.00)	0.05 (0.01-0.13)
Eastern Sub-Saharan Africa	27.40 (21.07-34.97)	11.82 (9.63-14.37)	7.60 (4.61-11.91)	5.56 (3.65-8.05)	0.34 (0.18-0.49)	1.63 (1.17-2.19)	0.19 (0.12-0.29)	0.21 (0.02-0.97)	0.06 (0.02-0.13)
Southern Sub-Saharan Africa	21.18 (16.21-27.38)	9.75 (7.98-11.86)	7.24 (4.55-10.93)	2.74 (1.81-4.05)	0.32 (0.18-0.51)	0.69 (0.52-0.92)	0.20 (0.14-0.29)	0.20 (0.02-0.78)	0.04 (0.01-0.08)
Western Sub-Saharan Africa	27.95 (21.37-36.43)	12.33 (10.04-15.08)	7.71 (4.70-11.99)	4.72 (2.93-7.11)	0.31 (0.19-0.45)	2.50 (1.67-3.70)	0.14 (0.10-0.18)	0.21 (0.02-0.92)	0.05 (0.01-0.11)

KP=Klebsiella pnenmoniae. LRIs=Lower respiratory infections . UTIs=Urinary tract infections. CNS=central nervous system. GBD=Global Burden of Diseases, Injuries, and Risk Factors Study.

Table S2 Deaths for infectious syndromes associated with and attributable to KP-AMR, by pathogen-drug combination, 2019

	Aminoglycosides AWR	Aminoglycosides ATR	Beta Lactam/Beta-lactamase inhibitors AWR	Beta Lactam/Beta-lactamase inhibitors ATR	Carbapenems AWR	Carbapenems ATR	Fluoroquinolones AWR	Fluoroquinolones ATR	Third-generation cephalosporins AWR	Third-generation cephalosporins ATR	Trimethoprim-Sulfamethoxazole AWR	Trimethoprim-Sulfamethoxazole ATR
Global	345457 (253863-463161)	26343 (16213-38879)	544713 (396427-724808)	7933 (4586-12068)	234168 (164262-331603)	55666 (36284-81337)	425750 (311649-575889)	29023 (17450-44078)	526235 (383648-716646)	50141 (18971-94411)	487640 (356201-653339)	23485 (11522-39671)
East Asia	5362 (3091-8845)	594 (316-1021)	27209 (17008-42625)	291 (15-1088)	12425 (1901-37159)	3322 (660-10434)	25052 (15808-38881)	2000 (1113-3286)	40102 (16142-76536)	5639 (917-14554)	41344 (25596-63495)	2635 (1167-4925)
Southeast Asia	9764 (6542-14231)	908 (543-1392)	37355 (26079-51843)	320 (151-563)	18238 (12571-26187)	4679 (3052-7043)	31439 (22207-44413)	2463 (1465-3779)	44118 (30964-60645)	5173 (2017-9539)	31989 (22319-44585)	1698 (808-2886)
Oceania	596 (367-904)	47 (26-74)	1015 (710-1415)	14 (2-43)	150 (37-485)	39 (13-110)	932 (638-1304)	68 (39-108)	1233 (642-1881)	185 (49-374)	861 (588-1238)	42 (19-75)
Central Asia	2891 (1934-4313)	221 (130-351)	6220 (4256-8818)	87 (26-186)	943 (415-1904)	231 (108-453)	4227 (2896-6039)	289 (169-459)	6229 (4029-9275)	885 (312-1696)	6231 (4248-8948)	328 (156-572)
Central Europe	6215 (4052-9402)	470 (271-764)	9847 (6390-14679)	168 (88-275)	2846 (1750-4396)	624 (353-1033)	8125 (5264-11996)	552 (313-891)	8975 (5786-13599)	962 (347-1884)	8036 (5169-12000)	368 (172-674)
Eastern Europe	14822 (9517-22439)	1107 (622-1796)	21323 (13889-30695)	196 (79-389)	13378 (8522-19782)	2946 (1732-4587)	18297 (11952-26536)	1222 (695-1930)	19921 (12972-28766)	1142 (355-2449)	19139 (12473-27993)	848 (389-1531)
High-income Asia Pacific	1292 (909-1794)	129 (76-199)	5507 (3977-7535)	313 (185-487)	245 (109-499)	113 (53-200)	2025 (1461-2812)	173 (103-266)	3848 (2716-5425)	649 (265-1215)	3411 (2497-4539)	233 (114-390)
Australasia	124 (77-188)	13 (7-21)	434 (290-636)	26 (11-45)	78 (15-254)	25 (8-79)	145 (91-217)	13 (7-21)	227 (85-447)	33 (8-85)	514 (339-774)	40 (18-74)
Western Europe	8642 (5981-12111)	705 (424-1113)	19190 (13439-26570)	850 (484-1320)	3559 (2107-5744)	907 (498-1530)	13247 (9318-18330)	981 (576-1534)	13907 (9810-19650)	1660 (615-3297)	14407 (10261-20143)	814 (399-1411)
Southern Latin America	2839 (2061-3975)	225 (137-338)	5542 (4162-7318)	102 (56-164)	1779 (1146-2651)	392 (241-606)	4611 (3463-6227)	337 (206-498)	5635 (4207-7468)	665 (255-1250)	4475 (3369-5930)	223 (111-374)
High-income North America	3756 (2580-5407)	324 (193-520)	8611 (5933-12111)	447 (253-699)	1565 (722-2757)	434 (216-741)	4973 (3457-7116)	391 (229-626)	6261 (4143-9180)	795 (278-1615)	6354 (4347-9006)	410 (197-721)
Caribbean	1953 (1335-2767)	145 (85-226)	3809 (2612-5396)	84 (34-167)	348 (122-874)	92 (39-205)	2664 (1823-3741)	179 (102-282)	3450 (2235-5100)	503 (192-968)	3471 (2386-4857)	171 (83-296)
Andean Latin America	4492 (3125-6237)	332 (198-503)	5968 (4169-8252)	111 (37-223)	980 (355-1951)	231 (86-474)	4090 (2810-5643)	253 (145-393)	4975 (3227-7220)	570 (186-1162)	5756 (4039-7908)	262 (128-442)
Central Latin America	5194 (3324-7641)	443 (258-708)	14375 (9697-20165)	394 (146-783)	2829 (1475-5043)	722 (361-1328)	7541 (5004-10689)	567 (324-892)	13185 (7907-19998)	1914 (655-3793)	11561 (7859-16469)	635 (308-1114)
Tropical Latin America	10159 (7161-14428)	793 (467-1240)	17268 (12566-23115)	458 (213-807)	9074 (6020-12850)	2010 (1210-2997)	13920 (10220-19003)	1019 (619-1547)	13553 (7877-20182)	1011 (267-2310)	12663 (9268-17207)	611 (299-1029)
North Africa and Middle East	14816 (9902-21407)	1172 (698-1839)	32447 (21742-46484)	383 (174-738)	13257 (8231-19611)	3131 (1802-4870)	22212 (14580-31943)	1588 (920-2539)	32728 (21692-47324)	3680 (1385-6964)	27309 (18418-39132)	1362 (640-2389)
South Asia	123193 (86253-171695)	9481 (5740-14162)	156409 (110444-215304)	1472 (778-2359)	131869 (91777-186662)	31195 (20203-47306)	133907 (94073-186081)	9047 (5344-13929)	151832 (106293-212519)	5582 (1896-11434)	117779 (83320-163419)	5016 (2354-8698)
Central Sub-Saharan Africa	13308 (9725-17631)	946 (577-1419)	16803 (12699-22009)	270 (51-654)	490 (96-1675)	164 (59-403)	12731 (9548-16680)	772 (459-1186)	14973 (9179-21256)	1948 (677-3738)	16664 (12509-21764)	750 (363-1233)
Eastern Sub-Saharan Africa	46428 (35559-60302)	3361 (2133-4983)	58897 (45162-75681)	843 (427-1388)	6470 (4370-9280)	1411 (867-2141)	40382 (30785-52322)	2454 (1489-3689)	52482 (39872-68983)	6559 (2565-12110)	57109 (43926-73595)	2554 (1259-4147)
Southern Sub-Saharan Africa	1980 (1393-2760)	165 (99-245)	9026 (6874-11800)	89 (19-199)	1308 (973-1793)	353 (235-507)	4315 (3239-5710)	320 (198-478)	9444 (7066-12500)	1485 (595-2655)	9595 (7331-12380)	554 (281-899)
Western Sub-Saharan Africa	67603 (51099-87913)	4761 (2993-6931)	87475 (66654-112165)	1016 (532-1680)	12271 (3920-27160)	2631 (989-5789)	70921 (54060-91434)	4336 (2611-6604)	79184 (60561-103223)	9115 (3340-16778)	88982 (68183-114129)	3932 (1896-6399)

95% uncertainty intervals are shown in parentheses. AWR=Associated with resistance; ATR=Attributable to resistance; AMR: antimicrobial resistance; KP=Klebsiella pneumoniae.

Table S3 All-age mortality rates for infectious syndromes linked to KP-AMR, by pathogen-drug combination, 2019

	Aminoglycosides		Beta Lactam/Beta-lactamase inhibitors		Carbapenems		Fluoroquinolones		Third-generation cephalosporins		Trimethoprim-Sulfamethoxazole	
	AWR	ATR	AWR	ATR	AWR	ATR	AWR	ATR	AWR	ATR	AWR	ATR
Global	4.46 (3.28-5.99)	0.34 (0.21-0.50)	7.04 (5.12-9.37)	0.10 (0.06-0.16)	3.03 (2.12-4.29)	0.72 (0.47-1.05)	5.50 (4.03-7.44)	0.38 (0.23-0.57)	6.80 (4.96-9.26)	0.65 (0.25-1.22)	6.30 (4.60-8.44)	0.30 (0.15-0.51)
East Asia	0.36 (0.21-0.60)	0.04 (0.02-0.07)	1.85 (1.16-2.90)	0.02 (0.00-0.07)	0.84 (0.13-2.52)	0.23 (0.04-0.71)	1.70 (1.07-2.64)	0.14 (0.08-0.22)	2.72 (1.10-5.20)	0.38 (0.06-0.99)	2.81 (1.74-4.31)	0.18 (0.08-0.34)
Southeast Asia	1.45 (0.97-2.11)	0.14 (0.08-0.21)	5.54 (3.87-7.69)	0.05 (0.02-0.08)	2.71 (1.87-3.89)	0.69 (0.45-1.05)	4.67 (3.30-6.59)	0.37 (0.22-0.56)	6.55 (4.60-9.00)	0.77 (0.30-1.42)	4.75 (3.31-6.62)	0.25 (0.12-0.43)
Oceania	4.49 (2.76-6.81)	0.35 (0.20-0.56)	7.65 (5.35-10.66)	0.10 (0.01-0.32)	1.13 (0.28-3.65)	0.29 (0.10-0.83)	7.02 (4.81-9.82)	0.51 (0.30-0.81)	9.29 (4.84-14.16)	1.39 (0.37-2.82)	6.48 (4.43-9.32)	0.32 (0.15-0.57)
Central Asia	3.09 (2.07-4.61)	0.24 (0.14-0.38)	6.65 (4.55-9.43)	0.09 (0.03-0.20)	1.01 (0.44-2.04)	0.25 (0.12-0.48)	4.52 (3.10-6.46)	0.31 (0.18-0.49)	6.66 (4.31-9.92)	0.95 (0.33-1.81)	6.66 (4.54-9.57)	0.35 (0.17-0.61)
Central Europe	5.44 (3.55-8.23)	0.41 (0.24-0.67)	8.62 (5.59-12.85)	0.15 (0.08-0.24)	2.49 (1.53-3.85)	0.55 (0.31-0.90)	7.11 (4.61-10.50)	0.48 (0.27-0.78)	7.86 (5.07-11.91)	0.84 (0.30-1.65)	7.04 (4.53-10.51)	0.32 (0.15-0.59)
Eastern Europe	7.06 (4.53-10.69)	0.53 (0.30-0.86)	10.16 (6.61-14.62)	0.09 (0.04-0.19)	6.37 (4.06-9.42)	1.40 (0.83-2.18)	8.71 (5.69-12.64)	0.58 (0.33-0.92)	9.49 (6.18-13.70)	0.54 (0.17-1.17)	9.12 (5.94-13.33)	0.40 (0.19-0.73)
High-income Asia Pacific	0.69 (0.49-0.96)	0.07 (0.04-0.11)	2.94 (2.12-4.02)	0.17 (0.10-0.26)	0.13 (0.06-0.27)	0.06 (0.03-0.11)	1.08 (0.78-1.50)	0.09 (0.05-0.14)	2.05 (1.45-2.90)	0.35 (0.14-0.65)	1.82 (1.33-2.42)	0.12 (0.06-0.21)
Australasia	0.43 (0.26-0.65)	0.04 (0.02-0.07)	1.49 (1.00-2.19)	0.09 (0.04-0.15)	0.27 (0.05-0.87)	0.09 (0.03-0.27)	0.50 (0.31-0.75)	0.04 (0.02-0.07)	0.78 (0.29-1.54)	0.12 (0.03-0.29)	1.77 (1.16-2.66)	0.14 (0.06-0.25)
Western Europe	1.98 (1.37-2.78)	0.16 (0.10-0.26)	4.40 (3.08-6.09)	0.20 (0.11-0.30)	0.82 (0.48-1.32)	0.21 (0.11-0.35)	3.04 (2.14-4.20)	0.23 (0.13-0.35)	3.19 (2.25-4.50)	0.38 (0.14-0.76)	3.30 (2.35-4.62)	0.19 (0.09-0.32)
Southern Latin America	4.25 (3.09-5.95)	0.34 (0.21-0.51)	8.30 (6.24-10.96)	0.15 (0.08-0.25)	2.66 (1.72-3.97)	0.59 (0.36-0.91)	6.91 (5.19-9.33)	0.51 (0.31-0.75)	8.44 (6.30-11.19)	1.00 (0.38-1.87)	6.70 (5.05-8.88)	0.33 (0.17-0.56)
High-income North America	1.03 (0.71-1.48)	0.09 (0.05-0.14)	2.36 (1.63-3.32)	0.12 (0.07-0.19)	0.43 (0.20-0.76)	0.12 (0.06-0.20)	1.36 (0.95-1.95)	0.11 (0.06-0.17)	1.72 (1.14-2.52)	0.22 (0.08-0.44)	1.74 (1.19-2.47)	0.11 (0.05-0.20)
Caribbean	4.14 (2.83-5.87)	0.31 (0.18-0.48)	8.08 (5.54-11.44)	0.18 (0.07-0.35)	0.74 (0.26-1.85)	0.20 (0.08-0.43)	5.65 (3.87-7.93)	0.38 (0.22-0.60)	7.31 (4.74-10.81)	1.07 (0.41-2.05)	7.36 (5.06-10.30)	0.36 (0.18-0.63)
Andean Latin America	7.06 (4.91-9.81)	0.52 (0.31-0.79)	9.38 (6.56-12.98)	0.17 (0.06-0.35)	1.54 (0.56-3.07)	0.36 (0.14-0.75)	6.43 (4.42-8.87)	0.40 (0.23-0.62)	7.82 (5.07-11.35)	0.90 (0.29-1.83)	9.05 (6.35-12.44)	0.41 (0.20-0.69)
Central Latin America	2.08 (1.33-3.06)	0.18 (0.10-0.28)	5.75 (3.88-8.07)	0.16 (0.06-0.31)	1.13 (0.59-2.02)	0.29 (0.15-0.53)	3.02 (2.00-4.28)	0.23 (0.13-0.36)	5.27 (3.16-8.00)	0.77 (0.26-1.52)	4.62 (3.14-6.59)	0.25 (0.12-0.45)
Tropical Latin America	4.54 (3.20-6.45)	0.36 (0.21-0.55)	7.72 (5.62-10.34)	0.21 (0.10-0.36)	4.06 (2.69-5.75)	0.90 (0.54-1.34)	6.23 (4.57-8.50)	0.46 (0.28-0.69)	6.06 (3.52-9.03)	0.45 (0.12-1.03)	5.66 (4.15-7.70)	0.27 (0.13-0.46)
North Africa and Middle East	2.43 (1.63-3.52)	0.19 (0.12-0.30)	5.33 (3.57-7.64)	0.06 (0.03-0.12)	2.18 (1.35-3.22)	0.51 (0.30-0.80)	3.65 (2.40-5.25)	0.26 (0.15-0.42)	5.38 (3.56-7.77)	0.61 (0.23-1.14)	4.49 (3.03-6.43)	0.22 (0.11-0.39)
South Asia	6.82 (4.78-9.51)	0.53 (0.32-0.78)	8.66 (6.12-11.93)	0.08 (0.04-0.13)	7.30 (5.08-10.34)	1.73 (1.12-2.62)	7.42 (5.21-10.31)	0.50 (0.30-0.77)	8.41 (5.89-11.77)	0.31 (0.11-0.63)	6.52 (4.62-9.05)	0.28 (0.13-0.48)
Central Sub-Saharan Africa	10.12 (7.39-13.40)	0.72 (0.44-1.08)	12.77 (9.65-16.73)	0.21 (0.04-0.50)	0.37 (0.07-1.27)	0.12 (0.04-0.31)	9.68 (7.26-12.68)	0.59 (0.35-0.90)	11.38 (6.98-16.16)	1.48 (0.51-2.84)	12.67 (9.51-16.55)	0.57 (0.28-0.94)
Eastern Sub-Saharan Africa	11.28 (8.64-14.64)	0.82 (0.52-1.21)	14.30 (10.97-18.38)	0.21 (0.10-0.34)	1.57 (1.06-2.25)	0.34 (0.21-0.52)	9.81 (7.48-12.71)	0.60 (0.36-0.90)	12.75 (9.68-16.75)	1.59 (0.62-2.94)	13.87 (10.67-17.87)	0.62 (0.31-1.01)
Southern Sub-Saharan Africa	2.52 (1.77-3.51)	0.21 (0.13-0.31)	11.49 (8.75-15.02)	0.11 (0.02-0.25)	1.66 (1.24-2.28)	0.45 (0.30-0.65)	5.49 (4.12-7.27)	0.41 (0.25-0.61)	12.02 (8.99-15.91)	1.89 (0.76-3.38)	12.21 (9.33-15.76)	0.70 (0.36-1.14)
Western Sub-Saharan Africa	14.82 (11.2-19.27)	1.04 (0.66-1.52)	19.17 (14.61-24.58)	0.22 (0.12-0.37)	2.69 (0.86-5.95)	0.58 (0.22-1.27)	15.54 (11.85-20.04)	0.95 (0.57-1.45)	17.35 (13.27-22.62)	2.00 (0.73-3.68)	19.50 (14.94-25.01)	0.86 (0.42-1.40)

95% uncertainty intervals are shown in parentheses. AWR=Associated with resistance; ATR=Attributable to resistance; AMR: antimicrobial resistance; KP=Klebsiella pnenmoniae.
