# Supplementary material for: Global burden of Klebsiella pneumoniae infections and antimicrobial resistance in 2019
Source: BMC Infect Dis. 2025 Nov 21;25:1773. doi: 10.1186/s12879-025-12120-w (PMC12751905; doi:10.1186/s12879-025-12120-w)
Supplement: Supplementary file 2 — Supplementary Material 2 [file 12879_2025_12120_MOESM2_ESM.pdf]

Global and regional burden associated with *Klebsiella pneumoniae* infection and antimicrobial resistance in 2019

Contents of appendix 2

Section 1: List of abbreviations 3

Section 2: List of International Classification of Diseases (ICD) codes mapped to the Global Burden of Disease cause list for causes of death 4

Section 3: Supplementary data 11

Section 3.1: Figure 1 Global number of deaths and age-standardized mortality rates associated with *Klebsiella pneumoniae* infection in 204 countries and territories 11

Section 3.1.1: Figure 1A Deaths associated with *Klebsiella pneumoniae* infection in 204 countries and territories 11

Section 3.1.2: Figure 1B Global age-standardied mortality rates associated with *Klebsiella pneumoniae* infections in 204 countries and territories… 14

Section 3.2: Figure 2 Global and regional mortality associated with and attributable to antimicrobial- resistant *Klebsiella pneumoniae*, by infectious syndrome, 2019.. 17

Section 3.3: Figure 3 Global and regional mortality rates associated with and attributable to antimicrobial- resistant *Klebsiella pneumoniae*, all ages, by infectious syndrome, 2019 20

Section 3.4: Figure 4 Deaths (number and all-age rate) associated with and attributable to antimicrobial-resistant *Klebsiella pneumoniae* (KP-AMR) in 21 Global Burden of Disease regions, 2019 23

Section 3.4.1: Figure 4A Deaths linked to KP-AMR....................... 23

Section 3.4.2: Figure 4B All-age mortality rates linked to KP-AMR 24

Section 3.5: S.Figure 1 Deaths associated with and attributable to antimicrobial-resistant *K. pneumoniae* (KP-AMR) in 21 Global Burden of Disease regions caused by infectious syndromes in 2019.. 25
Section 3.6: S.Figure 2 Mortality rates at all ages linked to antimicrobial-resistant *K. pneumoniae* (KP-AMR) in 21 Global Burden of Disease regions, stratified by infectious syndrome in 2019 34

List of abbreviations

Abbreviation	Full phrase
ASMR	age-standardised mortality rates
AMR	antimicrobial resistance
ATR	attributable to resistance
AWR	associated with resistance
BSI	bloodstream infections
CNS	central nervous system
DALYs	disability-adjusted life-years
ESKAPE	Enterococcus faecium, Staphylococcus aureus, Klebsiella pneumoniae, Acinetobacter baumannii, Pseudomonas aeruginosa, and Enterobacter specie
GBD	Global Burden of Diseases, Injuries, and Risk Factors Study
IAI	intra-abdominal infection
ICD	International Classification of Diseases
KP	*Klebsiella pneumoniae*
LMICs	Low- and middle-income countries
LRI	lower respiratory infection
YLDs	years lived with disability
YLLs	years of life lost
UI	uncertainty interval
UTI	urinary tract infection
WHO	World Health Organization
3GCs	third-generation cephalosporins

List of International Classification of Diseases (ICD) codes mapped to the Global Burden of Disease cause list for causes of death

Cause	ICD10	ICD9
Communicable, maternal, neonatal, and nutritional diseases	A00-A00.9, A01.0-A14, A15-A28.9, A32-A39.9, A48.1-A48.2, A48.4-A48.5, A50-A58, A60-A60.9, A63-A63.8, A65-A65.0, A68-A70, A74, A74.8-A75.9, A77-A96.9, A98-A98.8, B00-B06.9, B10-B10.8, B15-B16.2, B17.0, B17.2, B19.1, B20-B27.9, B29.4, B33-B33.1, B33.3-B33.8, B47-B48.8, B50-B54.0, B55.0, B56-B57.5, B60-B60.8, B63, B65-B67.9, B69-B72.0, B74.3-B75, B77-B77.9, B83-B83.8, B90-B91, B94.1, B95-B95.5, B97.4-B97.6, C58-C58.0, D50.1-D50.8, D51-D52.0, D52.8-D53.9, D70.3, D89.3, E00-E02, E40-E46.9, E51-E61.9, E63-E64.0, E64.2-E64.9, F02.1, F02.4, F07.1, G00.0-G00.8, G03-G03.8, G04-G05.8, G14-G14.6, G21.3, H70-H70.9, I00, I02, I02.9, I98.0-I98.1, J00-J02.8, J03-J03.8, J04-J04.2, J05-J05.1, J06.0-J06.8, J09-J15.8, J16-J16.9, J20-J21.9, J36-J36.0, J91.0, K52.1, K67.0-K67.8, K75.3, K76.3, K77.0, K93.0-K93.1, M03.1, M12.1, M49.0-M49.1, M73.0-M73.1, M89.6, N74.1, N96, N98-N98.9, O00-O07.9, O09-O16.9, O20-O26.9, O28-O36.9, O40-O48.1, O60-O77.9, O80-O92.7, O96-O98.6, O98.8-P04.2, P04.5-P05.9, P07-P15.9, P19-P22.9, P23.0-P23.4, P24-P29.9, P35-P37.2, P37.5-P39.9, P50-P61.9, P70-P70.1, P70.3-P72.9, P74-P78.9, P80-P81.9, P83-P84, P90-P94.9, P96, P96.3-P96.4, P96.8, R19.7, U04-U04.9, U06-U06.9, U82-U89, Z16-Z16.3	001-001.9, 002.0-029, 032-034.9, 036-036.3, 036.5-037.9, 040, 040.1-041.0, 042-066.9, 070.0-070.2, 071-075.9, 078.3-078.7, 079-079.7, 080-083.9, 084.0-084.5, 084.7-084.9, 085.0, 086-088, 088.8-088.9, 090-101.6, 104-104.9, 120-124.9, 125.4-125.9, 127-127.1, 128-129.0, 136-136.2, 137-139.0, 181-181.9, 244.2, 260-263.9, 265-269.9, 281.0-281.9, 320.0-320.8, 321-323.9, 381-383.9, 390-390.9, 392, 392.9, 425.6, 460-464.4, 464.8-464.9, 465.0-465.8, 466-469, 470.0, 475-475.9, 476.9, 480-482.8, 483.0-483.9, 484.0-484.7, 487-489, 630-636.9, 638-638.9, 640-679.1, 716.0, 730.4-730.6, 760-760.6, 760.8-768, 768.2-770, 770.1-775.0, 775.4-779.3, 779.6-779.8, V09-V09.9
HIV/AIDS and sexually transmitted infections	A50-A58, A60-A60.9, A63-A63.8, B20-B24.9, B63, F02.4, I98.0, K67.0-K67.2, M03.1, M73.0-M73.1	042-044.9, 054.1, 090-099.9
HIV/AIDS	B20-B24.9, F02.4	042-044.9
HIV/AIDS–drug-susceptible tuberculosis	B20.0	
HIV/AIDS–multidrug-resistant tuberculosis without extensive drug resistance		
HIV/AIDS–extensively drug-resistant tuberculosis		
HIV/AIDS resulting in other diseases	B20, B20.1-B24.9, F02.4	042-044.9
Sexually transmitted infections excluding HIV	A50-A58, A60-A60.9, A63-A63.8, B63, I98.0, K67.0-K67.2, M03.1, M73.0-M73.1	054.1, 090-099.9
Syphilis	A50-A53.9, I98.0, K67.2, M03.1, M73.1	090-097.9
Chlamydial infection	A55-A56.8, K67.0	
Gonococcal infection	A54-A54.9, K67.1, M73.0	098-098.9
Other sexually transmitted infections	A57-A58, A63-A63.8, B63	099-099.9
Respiratory infections and tuberculosis	A10-A14, A15-A19.9, A48.1, A70, B90-B90.9, B97.4-B97.6, H70-H70.9, J00-J02.8, J03-J03.8, J04-J04.2, J05-J05.1, J06.0-J06.8, J09-J15.8, J16-J16.9, J20-J21.9, J36-J36.0, J91.0, K67.3, K93.0, M49.0, N74.1, P23.0-P23.4, P37.0, U04-U04.9, U84.3	010-019.9, 034.0, 079.6, 137-137.9, 138.0-138.9, 381-383.9, 460-464.4, 464.8-464.9, 465.0-465.8, 466-469, 470.0, 475-475.9, 476.9, 480-482.8, 483.0-483.9, 484.1-484.2, 484.6-484.7, 487-489, 730.4-730.6
Tuberculosis	A10-A14, A15-A19.9, B90-B90.9, K67.3, K93.0, M49.0, N74.1, P37.0, U84.3	010-019.9, 137-137.9, 138.0-138.9, 730.4-730.6
Drug-susceptible tuberculosis	A10-A14, A15-A19.9, B90-B90.9, K67.3, K93.0, M49.0, N74.1, P37.0	010-019.9, 137-137.9, 138.0-138.9, 730.4-730.6
Multidrug-resistant tuberculosis without extensive drug [illegible]	U84.3	
Extensively drug-resistant tuberculosis		
Lower respiratory infections	A48.1, A70, B97.4-B97.6, J09-J15.8, J16-J16.9, J20-J21.9, J91.0, P23.0-P23.4, U04-U04.9	079.6, 466-469, 470.0, 480-482.8, 483.0-483.9, 484.1-484.2, 484.6-484.7, 487-489
Upper respiratory infections	J00-J02.8, J03-J03.8, J04-J04.2, J05-J05.1, J06.0-J06.8, J36-J36.0	034.0, 460-464.4, 464.8-464.9, 465.0-465.8, 475-475.9, 476.9
Otitis media	H70-H70.9	381-383.9
Enteric infections	A00-A00.9, A01.0-A09.9, A80-A80.9, K52.1, R19.7	001-001.9, 002.0-009.9, 045-045.9, 138
Diarrheal diseases	A00-A00.9, A02-A02.0, A02.8-A07, A07.2-A07.4, A08-A09.9, K52.1, R19.7	001-001.9, 003.8-006.9, 007.4-007.8, 008.2-009.9
Typhoid and paratyphoid	A01.0-A01.4	002.0-002.9
Typhoid fever	A01.0	002.0
Paratyphoid fever	A01.1-A01.4	002.1-002.9
Invasive non-typhoidal Salmonella (iNTS)	A02.1-A02.2	003-003.7
Other intestinal infectious diseases	A07.0-A07.1, A07.8-A07.9, A80-A80.9	007-007.3, 007.9-008.1, 045-045.9, 138
Neglected tropical diseases and malaria	A68-A68.9, A69.2-A69.9, A75-A75.9, A77-A79.9, A82-A82.9, A90-A96.9, A98-A98.8, B33.0-B33.1, B50-B54.0, B55.0, B56-B57.5, B60-B60.8, B65-B67.9, B69-B72.0, B74.3-B75, B77-B77.9, B83-B83.8, K93.1, P37.1, U06-U06.9	060-061.8, 065-066.9, 071-071.9, 080-083.9, 084.0-084.5, 084.7-084.9, 085.0, 086-088, 088.8-088.9, 120-124.9, 125.4-125.9, 127-127.1, 128-129.0, 425.6
Malaria	B50-B54.0	084.0-084.5, 084.7-084.9
Leprosy	A30-A30.9	030-030.9
Chagas disease	B57-B57.5, K93.1	086-086.2, 086.9, 425.6
Leishmaniasis	B55.0	085.0
Visceral leishmaniasis	B55.0	085.0
African trypanosomiasis	B56-B56.9	086.3-086.5
Schistosomiasis	B65-B65.9	120-120.9
Cysticercosis	B69-B69.9	123.1
Cystic echinococcosis	B67-B67.4, B67.8-B67.9	122-122.4, 122.8-122.9
Dengue	A90-A91.9	061-061.8
Yellow fever	A95-A95.9	060-060.9
Rabies	A82-A82.9	071-071.9
Intestinal nematode infections	B77-B77.9	127.0
Ascariasis	B77-B77.9	127.0
Ebola virus disease	A98.4	
Zika virus disease	U06-U06.9	
Other neglected tropical diseases	A68-A68.9, A69.2-A69.9, A75-A75.9, A77-A79.9, A92-A94.0, A96-A96.9, A98-A98.3, A98.5-A98.8, B33.0-B33.1, B60-B60.8, B67.5-B67.7, B70-B71.9, B74.3-B75, B83-B83.8, P37.1	065-066.9, 080-083.9, 087-088, 088.8-088.9, 122.5-122.7, 123-123.0, 123.2-124.9, 125.4-125.6, 125.9, 127, 127.1, 128-129.0
Other infectious diseases	A20-A28.9, A32-A39.9, A48.2, A48.4-A48.5, A65-A65.0, A69-A69.1, A74, A74.8-A74.9, A81-A81.9, A83-A89.9, B00-B06.9, B10-B10.8, B15-B16.2, B17.0, B17.2, B19.1, B25-B27.9, B29.4, B33, B33.3-B33.8, B47-B48.8, B91, B94.1, B95-B95.5, D70.3, D89.3, F02.1, F07.1, G00.0-G00.8, G03-G03.8, G04-G05.8, G14-G14.6, G21.3, I00, I02, I02.9, I98.1, K67.8, K75.3, K76.3, K77.0, M49.1, M89.6, P35-P35.9, P37, P37.2, P37.5-P37.9, U82-U84, U85-U89, Z16-Z16.3	020-029, 032-034, 034.1-034.9, 036-036.3, 036.5-037.9, 040, 040.1-041.0, 046-054.0, 054.2-059.9, 062-064.9, 070.0-070.2, 072-075.9, 078.3-078.7, 079-079.5, 079.7, 100-101.6, 104-104.9, 136-136.2, 139-139.0, 320.0-320.8, 321-323.9, 390-390.9, 392, 392.9, 484.0, 484.3-484.5, 771.0-771.3, V09-V09.9
Meningitis	A39-A39.9, A87-A87.9, G00.0-G00.8, G03-G03.8	036-036.3, 036.5-036.9, 047-049.9, 320.0-320.8, 321-322.9
Encephalitis	A83-A86.4, B94.1, F07.1, G04-G05.8, G21.3	062-064.9, 139.0, 323, 323.4-323.9
Diphtheria	A36-A36.9	032-032.9
Whooping cough	A37-A37.9	033-033.9, 484.3
Tetanus	A33-A35.0	037-037.9, 771.3
Measles	B05-B05.9	055-055.9, 484.0

List of International Classification of Diseases (ICD) codes mapped to the Global Burden of Disease cause list for causes of death		
**Cause**	**ICD10**	**ICD9**
Varicella and herpes zoster	B01-B02.9, P35.8	052-053.9
Acute hepatitis	B15-B16.2, B17.0, B17.2, B19.1, P35.3	070.0-070.2
Acute hepatitis A	B15-B15.9	070.0-070.1
Acute hepatitis B	B16-B16.2, B17.0, B19.1, P35.3	070.2
Acute hepatitis C		
Acute hepatitis E	B17.2	
Other unspecified infectious diseases	A20-A28.9, A32-A32.9, A38-A38.9, A48.2, A48.4-A48.5, A65-A65.0, A69-A69.1, A74, A74.8-A74.9, A81-A81.9, A88-A89.9, B00-B00.9, B03-B04, B06-B06.9, B10-B10.8, B25-B27.9, B29.4, B33, B33.3-B33.8, B47-B48.8, B91, B95-B95.5, D70.3, D89.3, F02.1, G14-G14.6, I00, I02, I02.9, I98.1, K67.8, K75.3, K76.3, K77.0, M49.1, M89.6, P35-P35.2, P35.9, P37, P37.2, P37.5-P37.9, U82-U84, U85-U89, Z16-Z16.3	020-029, 034, 034.1-034.9, 040, 040.1-041.0, 046-046.9, 050-051.9, 054-054.0, 054.2-054.9, 056-059.9, 072-075.9, 078.3-078.7, 079-079.5, 079.7, 100-101.6, 104-104.9, 136-136.2, 139, 323.0-323.3, 390-390.9, 392, 392.9, 484.4-484.5, 771.0-771.2, V09-V09.9
Maternal and neonatal disorders	C58-C58.0, N96, N98-N98.9, O00-O07.9, O09-O16.9, O20-O26.9, O28-O36.9, O40-O48.1, O60-O77.9, O80-O92.7, O96-O98.6, O98.8-P04.2, P04.5-P05.9, P07-P15.9, P19-P22.9, P24-P29.9, P36-P36.9, P38-P39.9, P50-P61.9, P70-P70.1, P70.3-P72.9, P74-P78.9, P80-P81.9, P83-P84, P90-P94.9, P96, P96.3-P96.4, P96.8	181-181.9, 630-636.9, 638-638.9, 640-679.1, 760-760.6, 760.8-768, 768.2-770, 770.1-771, 771.4-775.0, 775.4-779.3, 779.6-779.8
Maternal disorders	C58-C58.0, N96, N98-N98.9, O00-O07.9, O09-O16.9, O20-O26.9, O28-O36.9, O40-O48.1, O60-O77.9, O80-O92.7, O96-O98.6, O98.8-O99.9	181-181.9, 630-636.9, 638-638.9, 640-679.1
Maternal haemorrhage	O20-O20.9, O43.2, O44-O46.9, O62-O62.9, O67-O67.9, O70, O72-O72.3	640-641.9, 661-661.9, 665, 666-666.9
Maternal sepsis and other maternal infections	O23-O23.9, O85-O86.8, O91-O91.2	659.3, 670-670.9
Maternal hypertensive disorders	O10-O16.9	642-642.9
Maternal obstructed labor and uterine rupture	O32-O33.9, O64-O66.9, O71-O71.9	652-653.9, 660-660.9, 665.0-665.3
Maternal abortion and miscarriage	N96, O01-O07.9	630-632.9, 634-636.9, 638-638.9, 646.3
Ectopic pregnancy	O00-O00.9	633-633.9
Indirect maternal deaths	O24-O25.3, O98-O98.6, O98.8-O99.9	646-646.2, 646.4-649.9
Late maternal deaths	O96-O97.9	
Maternal deaths aggravated by HIV/AIDS		
Other maternal disorders	C58-C58.0, N98-N98.9, O09-O09.9, O21-O22.9, O26-O26.9, O28-O31.8, O34-O36.9, O40-O43.1, O43.8-O43.9, O47-O48.1, O60-O61.9, O63-O63.9, O68-O69.9, O70.0-O70.9, O73-O77.9, O80-O84.9, O87-O90.9, O92-O92.7	181-181.9, 643-645.2, 650-651.9, 654-659.2, 659.4-659.9, 662-664.9, 665.4-665.9, 667-669.9, 671-679.1
Neonatal disorders	P00-P04.2, P04.5-P05.9, P07-P15.9, P19-P22.9, P24-P29.9, P36-P36.9, P38-P39.9, P50-P61.9, P70-P70.1, P70.3-P72.9, P74-P78.9, P80-P81.9, P83-P84, P90-P94.9, P96, P96.3-P96.4, P96.8	760-760.6, 760.8-768, 768.2-770, 770.1-771, 771.4-775.0, 775.4-779.3, 779.6-779.8
Neonatal preterm birth	P01.0-P01.1, P07-P07.3, P22-P22.9, P25-P28.9, P61.2, P77-P77.9	761.0-761.1, 765-765.9, 769-769.9, 770.2-770.9, 776.6, 777.5-777.6
Neonatal encephalopathy due to birth asphyxia and trauma	P01.7, P02-P03.9, P10-P15.9, P20-P21.9, P24-P24.9, P90-P91.9	761.7-763.9, 767-768, 768.2-768.9, 770.1, 772.1-772.9, 779.0-779.2
Neonatal sepsis and other neonatal infections	P36-P36.9, P38-P39.9	771.4-771.9
Hemolytic disease and other neonatal jaundice	P55-P59.9	773-774.9
Other neonatal disorders	P00-P01, P01.2-P01.6, P01.8-P01.9, P04-P04.2, P04.5-P05.9, P08-P09, P19-P19.9, P29-P29.9, P50-P54.9, P60-P61.1, P61.3-P61.9, P70-P70.1, P70.3-P72.9, P74-P76.9, P78-P78.9, P80-P81.9, P83-P84, P92-P94.9, P96, P96.3-P96.4, P96.8	760-760.6, 760.8-761, 761.2-761.6, 764-764.9, 766-766.9, 770, 771, 772-772.0, 775-775.0, 775.4-776.5, 776.7-777.4, 777.7-779, 779.3, 779.6-779.8
Nutritional deficiencies	D50.1-D50.8, D51-D52.0, D52.8-D53.9, E00-E02, E40-E46.9, E51-E61.9, E63-E64.0, E64.2-E64.9, M12.1	244.2, 260-263.9, 265-269.9, 281.0-281.9, 716.0
Protein-energy malnutrition	E40-E46.9, E64.0	260-263.9
Other nutritional deficiencies	D51-D52.0, D52.8-D53.9, E00-E02, E51-E61.9, E63-E64, E64.2-E64.9, M12.1	244.2, 265-269.9, 281.0-281.9, 716.0
Non-communicable diseases	A46-A46.0, A66-A67.9, B18-B18.9, B33.2, B86, C00-C13.9, C15-C22.8, C23-C25.9, C30-C34.9, C37-C38.8, C40-C41.9, C43-C45.9, C47-C54.9, C56-C57.8, C60-C63.8, C64-C67.9, C68.0-C68.8, C69.0-C69.8, C70-C73.9, C75-C75.8, C81-C86.6, C88-C91.0, C91.2-C91.3, C91.6, C92-C92.6, C93-C93.1, C93.3, C93.8, C94-C96.9, D00.1-D00.2, D01.0-D01.3, D02.0-D02.3, D03-D06.9, D07.0-D07.2, D07.4-D07.5, D09.0, D09.2-D09.3, D09.8, D10.0-D10.7, D11-D12.9, D13.0-D13.7, D14.0-D14.3, D15-D16.9, D22-D27.9, D28.0-D28.7, D29.0-D29.8, D30.0-D30.8, D31-D36, D36.1-D36.7, D37.1-D37.5, D38.0-D38.5, D39.1-D39.2, D39.8, D40.0-D40.8, D41.0-D41.8, D42-D43.9, D44.0-D44.8, D45-D47.9, D48.0-D48.6, D49.2-D49.4, D49.6, D52.1, D55-D58.9, D59.0-D59.3, D59.5-D59.6, D60-D61.9, D63.1, D64.0, D66-D67, D68.0-D69.8, D70-D70.2, D70.4-D75.8, D76-D78.8, D86-D86.9, D89-D89.2, E03-E07.1, E09-E11.9, E15.0, E16.0-E16.9, E20-E34, E34.1-E34.8, E36-E36.8, E65-E68, E70-E85.2, E88-E89.9, F00-F02.0, F02.2-F02.3, F02.8-F03.9, F10-F16.9, F18-F18.9, F24, F50.0-F50.5, G10-G13.8, G20-G20.9, G21.0-G21.1, G23-G26.0, G30-G31.9, G35-G37.9, G40-G41.9, G45-G46.8, G47.3, G61-G61.9, G62.1, G70-G73.7, G90-G90.9, G93.7, G95-G95.9, G97-G97.9, H05.0-H05.1, I01-I01.9, I02.0, I05-I09.9, I11-I13.9, I20-I25.9, I27.0-I27.2, I28-I28.9, I30-I31.1, I31.8-I37.8, I38-I41.9, I42.1-I42.8, I43-I43.9, I47-I48.9, I51.0-I51.4, I60-I63.9, I65-I66.9, I67.0-I67.3, I67.5-I67.7, I68.0-I68.2, I69.0-I69.3, I70.2-I70.8, I71-I73.9, I77-I89.9, I95.2-I95.3, I97-I98, I98.2, I98.9, J30-J35.9, J37-J39.9, J41-J46.9, J60-J63.8, J65-J68.9, J70-J70.9, J82, J84-J84.9, J91, J91.8-J92.9, J95-J95.9, K20-K20.9, K22-K22.6, K22.8-K29.9, K31-K31.8, K35-K38.9, K40-K46.9, K50-K52.0, K52.2-K52.9, K55-K62.9, K63.5, K64-K64.9, K66.8, K67, K68, K70-K70.3, K71.7, K73-K75, K75.1-K75.2, K75.4-K76.2, K76.4-K77, K77.8, K80-K83.9, K85-K86.9, K90-K91.9, K92.8, K93.8-K95.8, L00-L05.9, L08-L08.9, L10-L14.0, L51-L51.9, L88-L89.9, L93-L93.2, L97-L98.4, M00-M03.0, M03.2-M03.6, M05-M09.8, M30-M36.8, M40-M43.1, M65-M65.0, M71.0-M71.1, M72.5-M72.6, M80-M82.8, M86.3-M86.4, M87-M87.1, M88-M89.0, M89.5, M89.7-M89.9, N00-N08.8, N10-N12.9, N13.6, N14-N16.8, N18-N18.9, N20-N23.0, N25-N28.1, N29-N30.3, N30.8-N32.0, N32.3-N32.4, N34-N34.3, N36-N36.9, N39-N39.2, N41-N41.9, N44-N44.0, N45-N45.9, N49-N49.9, N60-N60.9, N65-N65.1, N72-N72.0, N75-N77.8, N80-N81.9, N83-N83.9, N84.0-N84.1, N87-N87.9, N99-N99.9, P04.3-P04.4, P70.2, P96.0-P96.2, P96.5, Q00-Q07.9, Q10.4-Q18.9, Q20-Q28.9, Q30-Q36, Q37-Q45.9, Q50-Q87.8, Q89-Q89.8, Q90-Q93.9, Q95-Q99.8, R50.2, R78.0-R78.5, R95-R95.9, X45-X45.9, X65-X65.9, Y15-Y15.9	035-035.9, 036.4, 102-103.9, 133-133.6, 135-135.9, 140-148.9, 150-155.1, 155.3-158.9, 160-164.9, 170-175.9, 180-180.9, 182-183.8, 184.0-184.4, 184.8, 185-186.9, 187.1-187.8, 188-188.9, 189.0-189.8, 190-190.8, 191-193.9, 194.1-194.8, 200-204.0, 204.2, 205-205.3, 206-206.1, 207-208.9, 209.0-209.1, 209.4-209.5, 210.0-210.9, 211.0-211.8, 212.0-212.8, 213-213.9, 217-220.9, 221.0-221.8, 222.0-222.8, 223.0-223.8, 224-228.9, 229.0, 229.8, 230.1-230.8, 231.0-231.2, 232-232.9, 233.0-233.2, 233.4-233.5, 233.7, 234.0-234.8, 235.0, 235.4, 235.6-235.8, 236.0-236.2, 236.4-236.5, 236.7, 237-237.3, 237.5-237.9, 238.0-238.9, 239.2-239.4, 239.6, 240-243.9, 244.0-244.1, 244.3-244.8, 245-246.9, 251-259.1, 259.3-259.9, 270-273.9, 275-276, 277-277.2, 277.4-277.9, 278.0-278.8, 282-284.9, 286-286.5, 286.7-289.0, 289.4-289.7, 290-292.9, 294.1-294.9, 303-303.9, 304.0-304.8, 305.0, 305.2-305.8, 307.1, 327.2-327.8, 330-331.2, 331.5-332.0, 333-337.9, 340-341.9, 345-345.9, 349, 349.2-349.8, 353.8-353.9, 356-356.9, 357.0-357.1, 357.3-357.7, 358-359.9, 376.0-376.1, 391-391.9, 392.0, 393-398.9, 402-404.9, 410-414.9, 416.0-416.1, 417-417.9, 420-423, 423.1-423.9, 424.0-424.3, 424.8, 425.0-425.3, 425.5, 425.7-425.8, 427.0-427.3, 427.6-427.8, 429.0, 430-435.9, 437.0-437.2, 437.4-437.8, 440.2, 440.4, 441-443.9, 446-457, 457.1-457.9, 459, 459.1-459.3, 470, 470.9-474.9, 476-476.1, 477-479, 491-493.9, 495-504.9, 506-506.9, 508-509, 515, 516-517.8, 518.6-518.7, 518.9, 519.0-519.4, 530-530.0, 530.2-530.6, 531-536.1, 536.4, 537-537.6, 537.8, 538-543.9, 550-553.6, 555-558.9, 560-560.3, 560.8-560.9, 562-562.1, 564-564.7, 565-566.9, 569.0-569.7, 571-571.9, 572.2-573.0, 573.4-577.9, 579-583.9, 585-585.9, 588-590.9, 592-593.8, 594-599.6, 599.8, 601-602.9, 604-604.9, 608.2, 610-610.9, 617-618.9, 620-620.9, 621.4-621.9, 622.1-622.7, 629-629.8, 680-689, 694-695.5, 707-707.9, 710-711.9, 714-714.3, 714.8-714.9, 730.1, 732-732.9, 733.0-733.1, 740-749.0, 749.2-758.9, 759.0-759.8, 760.7, 775.1-775.3, 779.4-779.5, 788.0, 790.3, 798-798.0, E850, E860
Neoplasms	C00-C13.9, C15-C22.8, C23-C25.9, C30-C34.9, C37-C38.8, C40-C41.9, C43-C45.9, C47-C54.9, C56-C57.8, C60-C63.8, C64-C67.9, C68.0-C68.8, C69.0-C69.8, C70-C73.9, C75-C75.8, C81-C86.6, C88-C91.0, C91.2-C91.3, C91.6, C92-C92.6, C93-C93.1, C93.3, C93.8, C94-C96.9, D00.1-D00.2, D01.0-D01.3, D02.0-D02.3, D03-D06.9, D07.0-D07.2, D07.4-D07.5, D09.0, D09.2-D09.3, D09.8, D10.0-D10.7, D11-D12.9, D13.0-D13.7, D14.0-D14.3, D15-D16.9, D22-D24.9, D26.0-D27.9, D28.0-D28.1, D28.7, D29.0-D29.8, D30.0-D30.8, D31-D36, D36.1-D36.7, D37.1-D37.5, D38.0-D38.5, D39.1-D39.2, D39.8, D40.0-D40.8, D41.0-D41.8, D42-D43.9, D44.0-D44.8, D45-D47.9, D48.0-D48.6, D49.2-D49.4, D49.6, K62.0-K62.1, K63.5, N60-N60.9, N84.0-N84.1, N87-N87.9	140-148.9, 150-155.1, 155.3-158.9, 160-164.9, 170-175.9, 180-180.9, 182-183.8, 184.0-184.4, 184.8, 185-186.9, 187.1-187.8, 188-188.9, 189.0-189.8, 190-190.8, 191-193.9, 194.1-194.8, 200-204.0, 204.2, 205-205.3, 206-206.1, 207-208.9, 209.0-209.1, 209.4-209.5, 210.0-210.9, 211.0-211.8, 212.0-212.8, 213-213.9, 217-217.8, 219.0, 220-220.9, 221.0-221.8, 222.0-222.8, 223.0-223.8, 224-228.9, 229.0, 229.8, 230.1-230.8, 231.0-231.2, 232-232.9, 233.0-233.2, 233.4-233.5, 233.7, 234.0-234.8, 235.0, 235.4, 235.6-235.8, 236.1-236.2, 236.4-236.5, 236.7, 237-237.3, 237.5-237.9, 238.0-238.9, 239.2-239.4, 239.6, 569.0, 610-610.9, 622.1-622.2, 622.7
Lip and oral cavity cancer	C00-C08.9, D10.0-D10.5, D11-D11.9	140-145.9, 210.0-210.6, 235.0
Nasopharynx cancer	C11-C11.9, D10.6	147-147.9, 210.7-210.9

List of International Classification of Diseases (ICD) codes mapped to the Global Burden of Disease cause list for causes of death		
**Cause**	**ICD10**	**ICD9**
Other pharynx cancer	C09-C10.9, C12-C13.9, D10.7	146-146.9, 148-148.9
Oesophageal cancer	C15-C15.9, D00.1, D13.0	150-150.9, 211.0, 230.1
Stomach cancer	C16-C16.9, D00.2, D13.1, D37.1	151-151.9, 211.1, 230.2
Colon and rectum cancer	C18-C21.9, D01.0-D01.3, D12-D12.9, D37.3-D37.5	153-154.9, 209.1, 209.5, 211.3-211.4, 230.3-230.6, 569.0
Liver cancer	C22-C22.8, D13.4	155-155.1, 155.3-155.9, 211.5
Liver cancer due to hepatitis B		
Liver cancer due to hepatitis C		
Liver cancer due to alcohol use		
Liver cancer due to NASH		
Hepatoblastoma	C22.2	
Liver cancer due to other causes (internal)		
Gallbladder and biliary tract cancer	C23-C24.9, D13.5	156-156.9
Pancreatic cancer	C25-C25.9, D13.6-D13.7	157-157.9, 211.6-211.7
Larynx cancer	C32-C32.9, D02.0, D14.1, D38.0	161-161.9, 212.1, 231.0, 235.6
Tracheal, bronchus, and lung cancer	C33-C34.9, D02.1-D02.3, D14.2-D14.3, D38.1	162-162.9, 212.2-212.3, 231.1-231.2, 235.7
Malignant skin melanoma	C43-C43.9, D03-D03.9, D22-D23.9, D48.5	172-172.9
Non-melanoma skin cancer	C44-C44.9, D04-D04.9, D49.2	173-173.9, 222.4, 232-232.9, 238.2
Non-melanoma skin cancer (squamous-cell carcinoma)	C44-C44.9, D04-D04.9, D49.2	173-173.9, 222.4, 232-232.9, 238.2
Soft tissue and other extraosseous sarcomas	C49-C49.9	171-171.9
Malignant neoplasm of bone and articular cartilage	C40-C41.9	170-170.9
Breast cancer	C50-C50.9, D05-D05.9, D24-D24.9, D48.6, D49.3	174-175.9, 217-217.8, 233.0, 238.3, 239.3, 610-610.9
Cervical cancer	C53-C53.9, D06-D06.9, D26.0	180-180.9, 219.0, 233.1, 622.1-622.2, 622.7
Uterine cancer	C54-C54.9, D07.0-D07.2, D26.1-D26.9	182-182.9, 233.2
Ovarian cancer	C56-C56.9, D27-D27.9, D39.1	183-183.0, 220-220.9, 236.2
Prostate cancer	C61-C61.9, D07.5, D29.1, D40.0	185-185.9, 222.2, 236.5
Testicular cancer	C62-C62.9, D29.2-D29.8, D40.1-D40.8	186-186.9, 222.0, 222.3, 236.4
Kidney cancer	C64-C65.9, D30.0-D30.1, D41.0-D41.1	189.0-189.1, 189.5-189.6, 223.0-223.1
Bladder cancer	C67-C67.9, D09.0, D30.3, D41.4-D41.8, D49.4	188-188.9, 223.3, 233.7, 236.7, 239.4
Brain and central nervous system cancer	C70-C72.9	191-192.9
Eye cancer	C69.0-C69.8	190-190.8
Retinoblastoma	C69.2	190.5
Other eye cancers	C69.0-C69.1, C69.3-C69.8	190-190.4, 190.6-190.8
Neuroblastoma and other peripheral nervous cell tumors	C47-C47.9	
Thyroid cancer	C73-C73.9, D09.3, D09.8, D34-D34.9, D44.0	193-193.9, 226-226.9
Mesothelioma	C45-C45.9	
Hodgkin lymphoma	C81-C81.9	201-201.9
Non-Hodgkin lymphoma	C82-C86.6, C96-C96.9	200-200.9, 202-202.9
Burkitt lymphoma	C83.7-C83.8	200.2
Other non-Hodgkin lymphoma	C82-C83.6, C83.9-C86.6, C96-C96.9	200-200.1, 200.3-200.9, 202-202.9
Multiple myeloma	C88-C90.9	203-203.9
Leukaemia	C91-C91.0, C91.2-C91.3, C91.6, C92-C92.6, C93-C93.1, C93.3, C93.8, C94-C95.9	204-204.0, 204.2, 205-205.3, 206-206.1, 207-208.9
Acute lymphoid leukaemia	C91.0, C91.2-C91.3, C91.6	204.0, 204.2
Chronic lymphoid leukaemia		
Acute myeloid leukaemia	C92.0, C92.3-C92.6, C93.0, C94.0, C94.2, C94.4-C94.5	205.0, 205.2-205.3, 206.0, 207.0, 207.2-207.8
Chronic myeloid leukaemia	C92.1-C92.2	205.1
Other leukaemia	C93.1, C93.3, C93.8, C94.1, C94.3, C94.6-C95.9	206.1, 207.1, 207.9-208.9
Other malignant neoplasms (internal)	C17-C17.9, C30-C31.9, C37-C38.8, C48-C48.9, C4A, C51-C52.9, C57-C57.8, C60-C60.9, C63-C63.8, C66-C66.9, C68.0-C68.8, C75-C75.8, D07.4, D09.2, D13.2-D13.3, D14.0, D15-D16.9, D28.0-D28.1, D28.7, D29.0, D30.2, D30.4-D30.8, D31-D31.9, D35-D35.2, D35.5-D36, D36.1-D36.7, D37.2, D38.2-D38.5, D39.2, D39.8, D41.2-D41.3, D44.1-D44.8, D48.0-D48.4	152-152.9, 158-158.9, 160-160.9, 163-164.9, 183.2-183.8, 184.0-184.4, 184.8, 187.1-187.8, 189.2-189.4, 189.8, 194.1-194.8, 209.0, 209.4, 211.2, 211.8, 212.0, 212.4-212.8, 213-213.9, 221.0-221.8, 222.1, 222.8, 223.2, 223.8, 224-224.9, 227-228.9, 229.0, 229.8, 230.7-230.8, 233.4-233.5, 234.0-234.8, 235.4, 235.8, 236.1, 238.0-238.1, 239.2
Other neoplasms	D32-D33.9, D35.3-D35.4, D42-D43.9, D45-D47.9, D49.6, K62.0-K62.1, K63.5, N60-N60.9, N84.0-N84.1, N87-N87.9	225-225.9, 237-237.3, 237.5-237.9, 238.4-238.9, 239.6
Myelodysplastic, myeloproliferative, and other haematopoietic neoplasms	D45-D47.9	238.4-238.9
Cardiovascular diseases	B33.2, G45-G46.8, I01-I01.9, I02.0, I05-I09.9, I11-I11.9, I20-I25.9, I27.0, I27.2, I28-I28.9, I30-I31.1, I31.8-I37.8, I38-I41.9, I42.1-I42.8, I43-I43.9, I47-I48.9, I51.0-I51.4, I60-I63.9, I65-I66.9, I67.0-I67.3, I67.5-I67.6, I68.0-I68.2, I69.0-I69.3, I70.2-I70.8, I71-I73.9, I77-I83.9, I86-I89.0, I89.9, I98, K75.1	036.4, 391-391.9, 392.0, 393-398.9, 402-402.9, 410-414.9, 416.0, 417-417.9, 420-423, 423.1-423.9, 424.0-424.3, 424.8, 425.0-425.3, 425.5, 425.7-425.8, 427.0-427.3, 427.6-427.8, 429.0, 430-435.9, 437.0-437.2, 437.5-437.8, 440.2, 440.4, 441-443.9, 447-454.9, 456, 456.3-457, 457.1, 457.8-457.9, 459, 459.1-459.3
Rheumatic heart disease	I01-I01.9, I02.0, I05-I09.9	391-391.9, 392.0, 393-398.9
Ischaemic heart disease	I20-I25.9	410-414.9
Stroke	G45-G46.8, I60-I63.9, I65-I66.9, I67.0-I67.3, I67.5-I67.6, I68.1-I68.2, I69.0-I69.3	430-435.9, 437.0-437.2, 437.5-437.8
Ischaemic stroke	G45-G46.8, I63-I63.9, I65-I66.9, I67.2-I67.3, I67.5-I67.6, I69.3	433-435.9, 437.0-437.1, 437.5-437.8
Intracerebral haemorrhage	I61-I62, I62.1-I62.9, I68.1-I68.2, I69.1-I69.2	431-432.9, 437.2
Subarachnoid hemorrhage	I60-I60.9, I62.0, I67.0-I67.1, I69.0	430-430.9
Hypertensive heart disease	I11-I11.9	402-402.9
Non-rheumatic valvular heart disease	I34-I37.8	424.0-424.3, 424.8
Non-rheumatic calcific aortic valvular heart disease	I35-I35.9	424.1
Non-rheumatic degenerative mitral valvular heart disease	I34-I34.9	424.0
Other non-rheumatic valvular heart diseases	I36-I37.8	424.2-424.3, 424.8
Cardiomyopathy and myocarditis	B33.2, I40-I41.9, I42.1-I42.8, I43-I43.9, I51.4	422-422.9, 425.0-425.3, 425.5, 425.7-425.8, 429.0
Myocarditis	B33.2, I40-I41.9, I51.4	422-422.9
Alcoholic cardiomyopathy	I42.6	425.5
Other cardiomyopathy	I42.1-I42.5, I42.7-I42.8, I43-I43.9	425.0-425.3, 425.7-425.8, 429.0
Pulmonary arterial hypertension	I27.0, I27.2	416.0

List of International Classification of Diseases (ICD) codes mapped to the Global Burden of Disease cause list for causes of death

Cause	ICD10	ICD9
Atrial fibrillation and flutter	I48-I48.9	427.3
Aortic aneurysm	I71-I71.9	441-441.9
Peripheral artery disease	I70.2-I70.8, I73-I73.9	440.2, 440.4, 443.0-443.9
Endocarditis	I33-I33.9, I38-I39.9	421-421.9
Other cardiovascular and circulatory diseases (internal)	I28-I28.9, I30-I31.1, I31.8-I32.8, I47-I47.9, I51.0-I51.3, I68.0, I72-I72.9, I77-I83.9, I86-I89.0, I89.9, I98, K75.1	036.4, 417-417.9, 420-420.9, 423, 423.1-423.9, 427.0-427.2, 427.6-427.8, 442-443, 447-454.9, 456, 456.3-457, 457.1, 457.8-457.9, 459, 459.1-459.3
Chronic respiratory diseases	D86-D86.2, D86.9, G47.3, J30-J35.9, J37-J39.9, J41-J46.9, J60-J63.8, J65-J68.9, J70, J70.8-J70.9, J82, J84-J84.9, J91, J91.8-J92.9	135-135.9, 327.2-327.8, 470, 470.9-474.9, 476-476.1, 477-479, 491-493.9, 495-504.9, 506-506.9, 508-509, 515, 516-517.8, 518.6, 518.9, 519.1-519.4
Chronic obstructive pulmonary disease	J41-J44.9	491-492.9, 496-499
Pneumoconiosis	J60-J63.8, J65-J65.0, J92.0	500-504.9
Silicosis	J62-J62.9	502-502.9, 503.0, 503.9
Asbestosis	J61-J61.0, J92.0	501
Coal workers pneumoconiosis	J60-J60.0	500-500.9, 501.0-501.9
Other pneumoconiosis	J63-J63.8, J65-J65.0	503, 503.1, 504-504.9
Asthma	J45-J46.9	493-493.9
Interstitial lung disease and pulmonary sarcoidosis	D86-D86.2, D86.9, J84-J84.9	135-135.9, 515, 516-516.9
Other chronic respiratory diseases	G47.3, J30-J35.9, J37-J39.9, J66-J68.9, J70, J70.8-J70.9, J82, J91, J91.8-J92, J92.9	327.2-327.8, 470, 470.9-474.9, 476-476.1, 477-479, 495-495.9, 506-506.9, 508-509, 517-517.8, 518.6, 518.9, 519.1-519.4
Digestive diseases	B18-B18.9, I84-I85.9, I98.2, K20-K20.9, K22-K22.6, K22.8-K29.9, K31-K31.8, K35-K38.9, K40-K42.9, K44-K46.9, K50-K52, K52.2-K52.9, K55-K62, K62.2-K62.6, K62.8-K62.9, K64-K64.9, K66.8, K67, K68, K70-K70.3, K71.7, K73-K75, K75.2, K75.4-K76.2, K76.4-K77, K77.8, K80-K83.9, K85-K86.9, K90-K90.9, K92.8, K93.8, M09.1	455-455.9, 456.0-456.2, 530-530.0, 530.2-530.6, 531-536.1, 537-537.6, 537.8, 538, 540-543.9, 550-551.1, 551.3-552.1, 552.3-553.6, 555-558.9, 560-560.3, 560.8-560.9, 562-562.1, 564-564.1, 564.5-564.7, 565-566.9, 569.1-569.5, 569.7, 571-571.9, 572.2-573.0, 573.4-577.9, 579-579.2, 579.4-579.9
Cirrhosis and other chronic liver diseases	B18-B18.9, I85-I85.9, I98.2, K70-K70.3, K71.7, K73-K75, K75.2, K75.4-K76.2, K76.4-K76.9, K77.8	456.0-456.2, 571-571.9, 572.2-573.0, 573.4-573.9
Cirrhosis and other chronic liver diseases due to hepatitis B		
Cirrhosis and other chronic liver diseases due to hepatitis C		
Cirrhosis and other chronic liver diseases due to alcohol use		
Cirrhosis and other chronic liver diseases due to NAFLD		
Cirrhosis and other chronic liver diseases due to other causes		
Upper digestive system diseases	K25-K29.9	531-535.9
Peptic ulcer disease	K25-K28.9	531-534.9
Gastritis and duodenitis	K29-K29.9	535-535.9
Appendicitis	K35-K37.9, K38.3-K38.9	540-542.9
Paralytic ileus and intestinal obstruction	K56-K56.9	560-560.3, 560.8-560.9
Inguinal, femoral, and abdominal hernia	K40-K42.9, K44-K46.9	550-551.1, 551.3-552.1, 552.3-553.0, 553.6
Inflammatory bowel disease	K50-K52, K52.8-K52.9, M09.1	555-556.9, 558-558.9, 569.5
Vascular intestinal disorders	K55-K55.9	557-557.9
Gallbladder and biliary diseases	K80-K83.9	574-576.9
Pancreatitis	K85-K86.9	577-577.9, 579.4
Other digestive diseases	I84-I84.9, K20-K20.9, K22-K22.6, K22.8-K24, K31-K31.8, K38-K38.2, K52.2-K52.3, K57-K62, K62.2-K62.6, K62.8-K62.9, K64-K64.9, K66.8, K67, K68, K77, K90-K90.9, K92.8, K93.8	455-455.9, 530-530.0, 530.2-530.6, 536-536.1, 537-537.6, 537.8, 538, 543-543.9, 553.1-553.3, 562-562.1, 564-564.1, 564.5-564.7, 565-566.9, 569.1-569.4, 569.7, 579-579.2, 579.8-579.9
Neurological disorders	F00-F02.0, F02.2-F02.3, F02.8-F03.9, G10-G13.8, G20-G20.9, G23-G24, G24.1-G25.0, G25.2-G25.3, G25.5, G25.8-G26.0, G30-G31.1, G31.8-G31.9, G35-G37.9, G40-G41.9, G61-G61.9, G70-G71.1, G71.3-G72, G72.2-G73.7, G90-G90.9, G95-G95.9, M33-M33.9	290-290.9, 294.1-294.9, 330-331.2, 331.5-332.0, 333-337.9, 340-341.9, 345-345.9, 349, 349.2-349.8, 353.8-353.9, 356-356.9, 357.0-357.1, 357.3-357.4, 357.7, 358-359.9, 775.2
Alzheimer's disease and other dementias	F00-F02.0, F02.8-F03.9, G30-G31.1, G31.8-G31.9	290-290.9, 294.1-294.9, 331-331.2
Parkinson's disease	F02.3, G20-G20.9	332-332.0
Idiopathic epilepsy	G40-G41.9	345-345.9
Multiple sclerosis	G35-G35.9	340-340.9
Motor neuron disease	G12.2-G12.9	335-335.2, 335.8-335.9
Other neurological disorders	F02.2, G10-G12.1, G13-G13.8, G23-G24, G24.1-G25.0, G25.2-G25.3, G25.5, G25.8-G26.0, G36-G37.9, G61-G61.9, G70-G71.1, G71.3-G72, G72.2-G73.7, G90-G90.9, G95-G95.9, M33-M33.9	330-330.9, 331.5-331.9, 333-334.9, 335.3, 336-337.9, 341-341.9, 349, 349.2-349.8, 353.8-353.9, 356-356.9, 357.0-357.1, 357.3-357.4, 357.7, 358-359.9, 775.2
Mental disorders	F24, F50.0-F50.5	307.1
Eating disorders	F50.0-F50.5	307.1
Anorexia nervosa	F50.0-F50.1	307.1
Bulimia nervosa	F50.2-F50.5	
Substance use disorders	E24.4, F10-F16.9, F18-F18.9, G31.2, G62.1, G72.1, P04.3-P04.4, P96.1, Q86.0, R78.0-R78.5, X45-X45.9, X65-X65.9, Y15-Y15.9	291-292.9, 303-303.9, 304.0-304.8, 305.0, 305.2-305.8, 357.5, 760.7, 790.3, E850, E860
Alcohol use disorders	E24.4, F10-F10.9, G31.2, G62.1, G72.1, P04.3, Q86.0, R78.0, X45-X45.9, X65-X65.9, Y15-Y15.9	291-291.9, 303-303.9, 305.0, 357.5, 790.3, E860
Drug use disorders	F11-F16.9, F18-F18.9, P04.4, P96.1, R78.1-R78.5	292-292.9, 304.0-304.8, 305.2-305.8, 760.7, E850
Opioid use disorders	F11-F11.9, P96.1, R78.1	304.0, 305.5
Cocaine use disorders	F14-F14.9, R78.2	304.2, 305.6
Amphetamine use disorders	F15-F15.9	304.4, 305.7
Other drug use disorders	F13-F13.9, F16-F16.9, F18-F18.9, P04.4, R78.3-R78.5	292-292.9, 304.1, 304.5-304.8, 305.3-305.4, 305.8, 760.7
Diabetes and kidney diseases	D63.1, E10-E11.9, I12-I13.9, N00-N08.8, N15.0, N18-N18.9, P70.2, Q61-Q62.8	403-404.9, 580-583.9, 585-585.9, 589-589.9, 753-753.3, 775.1
Diabetes mellitus	E10-E10.1, E10.3-E11.1, E11.3-E11.9, P70.2	775.1
Diabetes mellitus type 1	E10-E10.1, E10.3-E10.9, P70.2	775.1
Diabetes mellitus type 2	E11-E11.1, E11.3-E11.9	
Chronic kidney disease	D63.1, E10.2, E11.2, I12-I13.9, N02-N08.8, N15.0, N18-N18.9, Q61-Q62.8	403-404.9, 581-583.9, 585-585.9, 589-589.9, 753-753.3
Chronic kidney disease due to diabetes mellitus type 1	E10.2	
Chronic kidney disease due to diabetes mellitus type 2	E11.2	
Chronic kidney disease due to hypertension	I12-I13.9	403-404.9
Chronic kidney disease due to glomerulonephritis	N03-N06.9	581-583.9
Chronic kidney disease due to other and unspecified causes	N02-N02.9, N07-N08.8, N15.0, Q61-Q62.8	589-589.9, 753-753.3

List of International Classification of Diseases (ICD) codes mapped to the Global Burden of Disease cause list for causes of death

Cause	ICD10	ICD9
Acute glomerulonephritis	N00-N01.9	580-580.9
Skin and subcutaneous diseases	A46-A46.0, A66-A67.9, B86, D86.3, I89.1-I89.8, L00-L05.9, L08-L08.9, L10-L14.0, L51-L51.9, L88-L89.9, L97-L98.4, M72.5-M72.6	035-035.9, 102-103.9, 133-133.6, 457.2-457.3, 680-689, 694-695.3, 707-707.9
Bacterial skin diseases	A46-A46.0, A66-A67.9, I89.1-I89.8, L00-L05.9, L08-L08.9, L88, L97-L98.4, M72.5-M72.6	035-035.9, 102-103.9, 457.2-457.3, 680-689
Cellulitis	L03-L03.9, M72.5-M72.6	681-682.9
Pyoderma	A46-A46.0, A66-A67.9, I89.1-I89.8, L00-L02.9, L04-L05.9, L08-L08.9, L88, L97-L98.4	035-035.9, 102-103.9, 457.2-457.3, 680-680.9, 683-689
Decubitus ulcer	L89-L89.9	707-707.9
Other skin and subcutaneous diseases	D86.3, L10-L14.0, L51-L51.9	694-695.3
Musculoskeletal disorders	I27.1, I67.7, L93-L93.2, M00-M03.0, M03.2-M03.6, M05-M09.0, M09.2-M09.8, M30-M32.9, M34-M36.8, M40-M43.1, M65-M65.0, M71.0-M71.1, M80-M82.8, M86.3-M86.4, M87-M87.0, M88-M89.0, M89.5, M89.7-M89.9	416.1, 437.4, 446-446.9, 695.4-695.5, 710-711.9, 714-714.3, 714.8-714.9, 730.1, 732-732.9, 733.0-733.1
Rheumatoid arthritis	M05-M06.9, M08.0-M08.8	714-714.3, 714.8-714.9
Other musculoskeletal disorders	I27.1, I67.7, L93-L93.2, M00-M03.0, M03.2-M03.6, M07-M08, M08.9-M09.0, M09.2-M09.8, M30-M32.9, M34-M36.8, M40-M43.1, M65-M65.0, M71.0-M71.1, M80-M82.8, M86.3-M86.4, M87-M87.0, M88-M89.0, M89.5, M89.7-M89.9	416.1, 437.4, 446-446.9, 695.4-695.5, 710-711.9, 730.1, 732-732.9, 733.0-733.1
Other non-communicable diseases	D25-D26, D28.2, D52.1, D55-D58.9, D59.0-D59.3, D59.5-D59.6, D60-D61.9, D64.0, D66-D67, D68.0-D69.8, D70-D70.2, D70.4-D75.8, D76-D78.8, D86.8, D89-D89.2, E03-E07.1, E09-E09.9, E15.0, E16.0-E16.9, E20-E24.3, E24.8-E34, E34.1-E34.8, E36-E36.8, E65-E68, E70-E85.2, E88-E89.9, G21.0-G21.1, G24.0, G25.1, G25.4, G25.6-G25.7, G71.2, G72.0, G93.7, G97-G97.9, I95.2-I95.3, I97-I97.9, I98.9, J70.0-J70.5, J95-J95.9, K43-K43.9, K52.0, K62.7, K91-K91.9, K94-K95.8, M87.1, N10-N12.9, N13.6, N14-N15, N15.1-N16.8, N20-N23.0, N25-N28.1, N29-N30.3, N30.8-N32.0, N32.3-N32.4, N34-N34.3, N36-N36.9, N39-N39.2, N41-N41.9, N44-N44.0, N45-N45.9, N49-N49.9, N65-N65.1, N72-N72.0, N75-N77.8, N80-N81.9, N83-N83.9, N99-N99.9, P96.0, P96.2, P96.5, Q00-Q07.9, Q10.4-Q18.9, Q20-Q28.9, Q30-Q36, Q37-Q45.9, Q50-Q60.6, Q63-Q86, Q86.1-Q87.8, Q89-Q89.8, Q90-Q93.9, Q95-Q99.8, R50.2, R95-R95.9	218-219, 219.1-219.9, 236.0, 240-243.9, 244.0-244.1, 244.3-244.8, 245-246.9, 251-259.1, 259.3-259.9, 270-273.9, 275-276, 277-277.2, 277.4-277.9, 278.0-278.8, 282-284.9, 286-286.5, 286.7-289.0, 289.4-289.7, 357.6, 518.7, 519.0, 536.4, 539-539.9, 551.2, 552.2, 564.2-564.4, 569.6, 579.3, 588-588.9, 590-590.9, 592-593.8, 594-599.6, 599.8, 601-602.9, 604-604.9, 608.2, 617-618.9, 620-620.9, 621.4-621.9, 622.3-622.6, 629-629.8, 740-749.0, 749.2-752.9, 753.4-758.9, 759.0-759.8, 775.3, 779.4-779.5, 788.0, 798-798.0
Congenital birth defects	G71.2, P96.0, Q00-Q07.9, Q10.4-Q18.9, Q20-Q28.9, Q30-Q36, Q37-Q45.9, Q50-Q60.6, Q63-Q86, Q86.1-Q87.8, Q89-Q89.8, Q90-Q93.9, Q95-Q99.8	740-749.0, 749.2-752.9, 753.4-758.9, 759.0-759.8
Neural tube defects	Q00-Q01.9, Q05-Q05.9	740-741.9, 742.0
Congenital heart anomalies	Q20-Q28.9	745-747.9
Orofacial clefts	Q35-Q36, Q37-Q37.9	749-749.0, 749.2-749.9
Down syndrome	Q90-Q90.9	758.0
Other chromosomal abnormalities	Q87-Q87.8, Q91-Q93.9, Q95-Q95.9, Q97-Q97.9, Q99-Q99.8	758, 758.1-758.6, 758.8-758.9
Congenital musculoskeletal and limb anomalies	Q65-Q79, Q79.6-Q79.9	742.5, 754-756.5, 756.8-756.9
Urogenital congenital anomalies	P96.0, Q50-Q60.6, Q63-Q64.9	752-752.9, 753.4-753.9
Digestive congenital anomalies	Q38-Q45.9, Q79.0-Q79.5	750-751.9, 756.6-756.7
Other congenital birth defects	G71.2, Q02-Q04.9, Q06-Q07.9, Q10.4-Q18.9, Q30-Q34.9, Q80-Q86, Q86.1-Q86.8, Q89-Q89.8	742, 742.1-742.4, 742.8-744.9, 748-748.9, 757-757.9, 759.0-759.8
Urinary diseases and male infertility	N10-N12.9, N13.6, N15, N15.1-N16.8, N20-N23.0, N25-N28.1, N29-N30.3, N30.8-N32.0, N32.3-N32.4, N34-N34.3, N36-N36.9, N39-N39.2, N41-N41.9, N44-N44.0, N45-N45.9, N49-N49.9	588-588.9, 590-590.9, 592-593.8, 594-598.1, 598.8-599.6, 599.8, 601-602.9, 604-604.9, 608.2, 788.0
Urinary tract infection and interstitial nephritis	N10-N12.9, N13.6, N15, N15.1-N16.8, N30-N30.3, N30.8-N30.9, N34-N34.3, N39.0-N39.2	590-590.9, 595-595.9, 597-597.9, 599.0
Urolithiasis	N20-N23.0	592-592.9, 594-594.9, 788.0
Other urinary diseases	N25-N28.1, N29-N29.8, N31-N32.0, N32.3-N32.4, N36-N36.9, N39, N41-N41.9, N44-N44.0, N45-N45.9, N49-N49.9	588-588.9, 593-593.8, 596-596.9, 598-598.1, 598.8-599, 599.1-599.6, 599.8, 601-602.9, 604-604.9, 608.2
Gynaecological diseases	D25-D26, D28.2, E28.2, N72-N72.0, N75-N77.8, N80-N81.9, N83-N83.9	218-219, 219.1-219.9, 236.0, 256.4, 617-618.9, 620-620.9, 621.4-621.9, 622.3-622.6, 629-629.8
Uterine fibroids	D25-D26, D28.2	218-219, 219.1-219.9, 236.0
Endometriosis	N80-N80.9	617-617.9
Genital prolapse	N81-N81.9	618-618.9
Other gynaecological diseases	N72-N72.0, N75-N77.8, N83-N83.9	620-620.9, 621.4-621.9, 622.3-622.6, 629-629.8
Haemoglobinopathies and haemolytic anaemias	D55-D58.9, D59.1, D59.3, D59.5, D60-D61.9, D64.0	282-284.9
Thalassaemias	D56-D56.9	282.4-282.5
Sickle cell disorders	D57-D57.8	282.6
G6PD deficiency	D55-D55.2	282.2-282.3
Other haemoglobinopathies and haemolytic anaemias	D55.3-D55.9, D58-D58.9, D59.1, D59.3, D59.5, D60-D61.9, D64.0	282-282.1, 282.7-284.9
Endocrine, metabolic, blood, and immune disorders	D52.1, D59.0, D59.2, D59.6, D66-D67, D68.0-D69.8, D70-D70.2, D70.4-D75.8, D76-D78.8, D86.8, D89-D89.2, E03-E07.1, E09-E09.9, E15.0, E16.0-E16.9, E20-E24.3, E24.8-E28.1, E28.3-E34, E34.1-E34.8, E36-E36.8, E65-E68, E70-E85.2, E88-E89.9, G21.0-G21.1, G24.0, G25.1, G25.4, G25.6-G25.7, G72.0, G93.7, G97-G97.9, I95.2-I95.3, I97-I97.9, I98.9, J70.0-J70.5, J95-J95.9, K43-K43.9, K52.0, K62.7, K91-K91.9, K94-K95.8, M87.1, N14-N14.4, N65-N65.1, N99-N99.9, P96.2, P96.5, R50.2	240-243.9, 244.0-244.1, 244.3-244.8, 245-246.9, 251-256.3, 256.8-259.1, 259.3-259.9, 270-273.9, 275-276, 277-277.2, 277.4-277.9, 278.0-278.8, 286-286.5, 286.7-289.0, 289.4-289.7, 357.6, 518.7, 519.0, 536.4, 539-539.9, 551.2, 552.2, 564.2-564.4, 569.6, 579.3, 598.2, 775.3, 779.4-779.5
Sudden infant death syndrome	R95-R95.9	798-798.0
Injuries	L55-L55.9, L56.3, L56.8-L56.9, L58-L58.9, N30.4, U00-U03, V00-V86.9, V87.2-V87.3, V88.2-V88.3, V90-V98.8, W00-W46.2, W49-W62.9, W64-W70.9, W73-W75.9, W77-W81.9, W83-W94.9, W97.9, W99-X06.9, X08-X39.9, X47-X48.9, X50-X54.9, X57-X58.9, X60-X64.9, X66-X83.9, X85-Y08.9, Y35-Y84.9, Y87.0-Y87.1, Y88-Y88.3, Y89.0-Y89.1	349.0-349.1, 457.0, E800-E807, E830-E838, E840-E849, E856-E857, E861-E865, E867-E869, E870-E876, E878-E879, E880-E886, E888-E928, E930-E979, E990-E999
Transport injuries	V00-V86.9, V87.2-V87.3, V88.2-V88.3, V90-V98.8	E800-E807, E830-E838, E840-E849
Road injuries	V01-V04.9, V06-V80.9, V82-V82.9, V87.2-V87.3	
Pedestrian road injuries	V01-V04.9, V06-V09.9	
Cyclist road injuries	V10-V19.9	
Motorcyclist road injuries	V20-V29.9	
Motor vehicle road injuries	V30-V79.9, V87.2-V87.3	
Other road injuries	V80-V80.9, V82-V82.9	
Other transport injuries	V00-V00.8, V05-V05.9, V81-V81.9, V83-V86.9, V88.2-V88.3, V90-V98.8	E800-E807, E830-E838, E840-E849
Unintentional injuries	L55-L55.9, L56.3, L56.8-L56.9, L58-L58.9, N30.4, W00-W46.2, W49-W62.9, W64-W70.9, W73-W75.9, W77-W81.9, W83-W94.9, W97.9, W99-X06.9, X08-X39.9, X47-X48.9, X50-X54.9, X57-X58.9, Y40-Y84.9, Y88-Y88.3	349.0-349.1, 457.0, E856-E857, E861-E865, E867-E869, E870-E876, E878-E879, E880-E886, E888-E928, E930-E949
Falls	W00-W19.9	E880-E886, E888
Drowning	W65-W70.9, W73-W74.9	E910

List of International Classification of Diseases (ICD) codes mapped to the Global Burden of Disease cause list for causes of death		
**Cause**	**ICD10**	**ICD9**
Fire, heat, and hot substances	X00-X06.9, X08-X19.9	E890-E899, E924
Poisonings	X47-X48.9	E856-E857, E861-E865, E867-E869
Poisoning by carbon monoxide	X47-X47.9	E862, E868-E869
Poisoning by other means	X48-X48.9	E856-E857, E861, E863-E865, E867
Exposure to mechanical forces	W20-W38.9, W40-W43.9, W45.0-W45.2, W46-W46.2, W49-W52	E916-E922
Unintentional firearm injuries	W32-W34.9	E922
Other exposure to mechanical forces	W20-W31.9, W35-W38.9, W40-W43.9, W45.0-W45.2, W46-W46.2, W49-W52	E916-E921
Adverse effects of medical treatment	N30.4, Y40-Y84.9, Y88-Y88.3	349.0-349.1, 457.0, E870-E876, E878-E879, E930-E949
Animal contact	W52.0-W62.9, W64-W64.9, X20-X29.9	E905-E906
Venomous animal contact	X20-X29.9	E905
Non-venomous animal contact	W52.0-W62.9, W64-W64.9	E906
Foreign body	W44-W45, W45.3-W45.9, W75-W75.9, W78-W80.9, W83-W84.9	E911-E915
Pulmonary aspiration and foreign body in airway	W75-W75.9, W78-W80.9, W83-W84.9	E911-E913
Foreign body in other body part	W44-W45, W45.3-W45.9	E914-E915
Environmental heat and cold exposure	L55-L55.9, L56.3, L56.8-L56.9, L58-L58.9, W88-W94.9, W97.9, W99-W99.9, X30-X32.9, X39-X39.9	E900-E902, E926
Exposure to forces of nature	X33-X38.9	E907-E909
Still Born	P95-P95.9	768.0-768.1
Other unintentional injuries	W39-W39.9, W77-W77.9, W81-W81.9, W85-W87.9, X50-X54.9, X57-X58.9	E903-E904, E923, E925, E927-E928
Self-harm and interpersonal violence	U00-U03, X60-X64.9, X66-X83.9, X85-Y08.9, Y35-Y38.9, Y87.0-Y87.1, Y89.0-Y89.1	E950-E979, E990-E999
Self-harm	X60-X64.9, X66-X83.9, Y87.0	E950-E959
Self-harm by firearm	X72-X74.9	E955
Self-harm by other specified means	X60-X64.9, X66-X71.9, X75-X83.9, Y87.0	E950-E954, E956-E959
Interpersonal violence	X85-Y08.9, Y87.1	E960-E969
Physical violence by firearm	X93-X95.9	E965
Physical violence by sharp object	X99-X99.9	E966
Physical violence by other means	X85-X92.9, X96-X98.9, Y00-Y04.9, Y06-Y08.9, Y87.1	E961-E964, E967-E969
Conflict and terrorism	U00-U03, Y36-Y38.9, Y89.1	E979, E990-E999
Police conflict and executions	Y35-Y35.9, Y89.0	E970-E978
Garbage Code (GBD Level 1)	A40-A41.9, A48.0, A48.3, A49.0-A49.1, A59-A59.9, A71-A71.9, A74.0, B07-B07.9, B30-B30.9, B35-B36.9, B85-B85.4, B87-B88.9, B94.0, D50-D50.0, D50.9, D62-D63.0, D63.8-D64, D64.1-D65.9, D68, D69.9, E15, E16, E50-E50.9, E64.1, E85.3-E87.6, E87.8-E87.9, F06.2-F06.4, F07.2, F09-F09.9, F19-F23.9, F25-F49, F51-F99.0, G06-G08.0, G32-G32.8, G43-G44.2, G44.4-G44.8, G47-G47.2, G47.4-G47.9, G50-G60.9, G62-G62.0, G62.2-G65.2, G80-G83.9, G89-G89.4, G91-G91.2, G91.4-G93, G93.1-G93.2, G93.4-G93.6, G94.0-G94.8, G99-H05, H05.2-H69.9, H71-H99, I26-I26.9, I31.2-I31.4, I46-I46.9, I50.0-I50.4, I76, I95-I95.1, I95.8-I95.9, J69-J69.9, J80-J80.9, J81.0, J85-J85.3, J86-J86.9, J93-J93.1, J93.8-J93.9, J94.2, J96-J96.9, J98.1-J98.3, K00-K19, K30, K65-K66.1, K66.9, K68.1-K68.9, K71-K71.6, K71.8-K72.9, K75.0, L20-L30.9, L40-L50.9, L52-L54.8, L56-L56.2, L56.4-L56.5, L57-L57.9, L59-L68.9, L70-L76.8, L80-L87.9, L90-L92.9, L94-L96, L98.5-L99.8, M04, M10-M12.0, M12.2-M29, M37-M39, M43.2-M49, M49.2-M64, M65.1-M71, M71.2-M72.4, M72.8-M73, M73.8-M79.9, M83-M86.2, M86.5-M86.9, M87.2-M87.9, M89.1-M89.4, M90-M99.9, N17-N17.9, N19-N19.9, N32.1-N32.2, N32.8-N33.8, N35-N35.9, N37-N37.8, N39.3-N39.8, N42-N43.4, N44.1-N44.8, N46-N48.9, N50-N53.9, N61-N64.9, N82-N82.9, N91-N91.5, N95, N95.1-N95.9, N97-N97.9, R02-R02.9, R03.1, R07.0, R08-R09, R09.3, R11-R12.0, R14-R19.6, R19.8-R23, R23.1-R30.9, R32-R50.1, R50.8-R57.9, R58.0-R72.9, R74-R78, R78.6-R94.8, R96-R99.9, U05, U07-U81, U89.9-U99, X40-X44.9, X46-X46.9, X49-X49.9, Y10-Y14.9, Y16-Y19.9, Z00-Z15.8, Z17-unsp.	038-038.9, 040.0, 041.1, 076-078.2, 110-111.9, 125-125.3, 126-126.9, 127.2-127.9, 131-132.9, 133.8-134.9, 136.6, 139.1, 139.9, 247-248, 264-264.9, 274-274.9, 276.0-276.5, 276.7-276.9, 277.3, 280-281, 285-285.9, 286.6, 289.1-289.3, 293, 294-294.0, 295-302.9, 305, 305.9-307.0, 307.2-307.4, 307.6-319.9, 324-327.1, 328-329, 338-339.1, 339.3-339.8, 342-344.9, 346-346.9, 350-353.6, 354-355.9, 360-362, 362.1-376, 376.2-380.9, 384-389.9, 415-415.9, 423.0, 424, 424.4-424.5, 424.9, 427.5, 427.9-428.9, 437.3, 458-458.9, 459.0, 507-507.9, 510-510.9, 512-513.9, 518.1-518.2, 520-529.9, 536.3, 536.8-536.9, 537.7, 537.9, 564.8-564.9, 567-568.9, 570-570.9, 572-572.1, 573.1-573.3, 584-584.9, 586-587.9, 603-603.9, 605-608.1, 608.3-609, 611-612.1, 615-616.9, 619-619.9, 621-621.3, 622-622.0, 622.8-623.6, 623.8-624.5, 624.8-628.9, 629.9, 690-693.9, 695.8-706.9, 708-709.9, 712-713.8, 715-716, 716.2-721.6, 721.8-730.0, 730.2-730.3, 730.7-731.9, 733, 733.2-734.2, 737-738, 738.2-739.9, 780-782.4, 782.6-784.6, 784.9, 785.4-786, 786.6, 786.8, 787, 787.3-788, 788.3-789, 789.1-789.2, 789.5, 790-790.1, 790.4-796.1, 796.3-797.9, 798.1-799, 799.2-799.9, 999.0-999.9, E851-E855, E858, E866, E980-E982, V01-V08, V10-uns
Garbage Code (GBD Level 2)	A14.9, A29-A30.9, A45-A45.9, A47-A48, A48.8-A49, A49.3-A49.9, A61-A62, A72-A73, A76, A97, B08-B09, B11-B14, B28-B29, B31-B32.4, B34-B34.9, B61-B62, B68-B68.9, B73-B74.2, B76-B76.9, B78-B81.8, B84, B92-B94, B94.8-B94.9, B95.6-B97.3, B97.7-B99.9, D59, D59.4, D59.8-D59.9, F17-F17.9, G44.3, G91.3, G93.0, G93.3, I10-I10.9, I15-I15.9, I27, I27.8-I27.9, I50, I50.8-I50.9, I67.4, I70-I70.1, I70.9, I74-I75.8, J81, J81.1, J90-J90.0, J94-J94.1, J94.8-J94.9, K92.0-K92.2, N70-N71.9, N73-N74.0, N74.2-N74.8, R03-R03.0, R04-R06.9, R09.0-R09.2, R09.8-R10.9, R13-R13.9, R23.0, R58, S00-T98.3, W47-W48, W63, W71-W72, W76-W76.9, W82, W95-W97, W98, X07, X55-X56, X59-X59.9, Y20-Y34.9, Y86-Y87, Y87.2, Y89, Y89.9-Y99.9	000-000.9, 030-030.9, 041.2-041.9, 067-069, 078.8-078.9, 079.8-079.9, 089-089.9, 105-109.9, 119, 136.8-136.9, 139.8, 304, 304.9, 305.1, 339.2, 401-401.9, 405-405.9, 416, 416.2-416.9, 440-440.1, 440.3, 440.8-440.9, 444-445.8, 490-490.9, 494-494.9, 511-511.9, 514-514.9, 515.0-515.9, 518-518.0, 518.3-518.5, 518.8, 536.2, 578-578.9, 599.7, 613-614.9, 714.4, 716.1, 721.7, 735-736.9, 738.0-738.1, 784.7-784.8, 786.3, 787.0-787.2, 789.0, 789.3-789.4, 789.6-789.9, 796.2, 799.0-799.1, 800-999, E000-E80, E83, E839, E85, E859, E87, E877, E88, E887, E929, E983-E985, E988-E989
Garbage Code (GBD Level 3)	A01, A31-A31.9, A42-A44.9, A49.2, A64-A64.0, A99-A99.0, B17, B17.1, B17.8-B17.9, B19-B19.0, B19.2-B19.9, B37-B46.9, B49-B49.9, B55, B55.1-B55.9, B58-B59.9, B89, B94.2, C14-C14.9, C22.9, C26-C29, C35-C36, C39-C39.9, C42, C46-C46.9, C55-C55.9, C57.9, C59, C63.9, C68, C68.9, C74-C74.9, C75.9-C80.9, C87, C97-D00.0, D01, D01.4-D02, D02.4-D02.9, D07, D07.3, D07.6-D09, D09.1, D09.7, D09.9-D10, D10.9, D13, D13.9-D14, D14.4, D17-D21.9, D28, D28.9-D29, D29.9-D30, D30.9, D36.0, D36.9-D37.0, D37.6-D38, D38.6-D39.0, D39.7, D39.9-D40, D40.9-D41, D41.9, D44, D44.9, D48, D48.7-D49.1, D49.5, D49.7-D49.9, D54, D75.9, D79-D85, D87-D88, D89.8-D99, E07.8-E08.9, E17-E19, E34.0, E34.9-E35.8, E37-E39, E47-E49., E62, E69, E87.7, E90-E998, F04-F06.1, F06.5-F07.0, F07.8-F08, F50, F50.8-F50.9, G09-G09.9, G15-G19, G21, G21.2, G21.4-G22.0, G27-G29, G33-G34, G38-G39., G42, G48-G49, G66-G69, G74-G79, G84-G88, G93.8-G94, G96-G96.9, G98-G98.9, I00.0, I03-I04., I14-I14., I16-I19, I29-I29.9, I44-I45.9, I49-I49.9, I51, I51.6-I59, I90-I94, I96-I96.9, I98.4-I98.8, I99-ID5.9, J02.9, J03.9, J04.3, J06, J06.9, J40-J40.9, J47-J59, J71-J79, J81.9, J83, J85.9, J87-J89, J90.9, J93.6, J97-J98.0, J98.4-J99.8, K21-K21.9, K22.7, K31.9-K34, K39, K47-K49, K53-K54, K63-K63.4, K63.8-K63.9, K69, K70.4-K70.9, K78-K79, K84, K87-K89, K92, K92.9-K93, K96-K99, L06-L07, L09, L15-L19, L31-L39, L69, L77-L79, N09, N13-N13.5, N13.7-N13.9, N24, N28.8-N28.9, N38, N39.9-N40.9, N54-N59, N66-N69, N78-N79, N84, N84.2-N86, N88-N90.9, N92-N94.9, N95.0, O08-O08.9, O17-O19, O27, O37-O39, O49-O59, O78-O79, O93-O95.9, P06, P16-P18, P30-P34.2, P40-P49, P62-P69, P73, P79, P82, P85-P89, P96.9-P99.9, Q08-Q10.3, Q19, Q29-Q29., Q36.0-Q36.9, Q46-Q49, Q88, Q89.9, Q94, Q99.9-R01.2, R07, R07.1-R07.9, R31-R31.9	002, 031-031.9, 039-039.9, 070, 070.4-070.9, 085, 085.1-085.9, 088.0-088.7, 112-118.9, 130-130.9, 136.3-136.5, 149-149.9, 155.2, 159-159.9, 165-169, 176-179.9, 183.9-184, 184.5, 184.9, 187, 187.9, 189, 189.9, 190.9, 195-199.9, 209, 209.2-209.3, 209.6-210, 211, 211.9-212, 212.9, 214-216.9, 221, 221.9-222, 222.9-223, 223.9, 229, 229.1, 229.9-230.0, 230.9-231, 231.8-231.9, 233, 233.3, 233.6, 233.9-234, 234.9-235, 235.1-235.3, 235.5, 235.9-236, 236.3, 236.6, 236.9, 237.4, 239-239.1, 239.5, 239.7-239.9, 249-249.9, 259.2, 276.6, 278, 279-279.9, 293.0-293.9, 331.3-331.4, 332.1-332.9, 347-348.9, 349.9, 357, 357.8-357.9, 399-400.0, 406-409.4, 418-419.9, 426-427, 427.4, 429, 429.2-429.9, 459.5-459.9, 464.5, 465, 465.9, 505-505.9, 519, 519.8-519.9, 530.1, 530.7-530.9, 544-549, 553.8-553.9, 559-559.0, 560.4-560.7, 561, 562.2-563, 569, 569.8-569.9, 591-591.9, 593.9, 599.9-600.9, 623.7, 624.6, 637-637.9, 639-639.9, 749.1, 759, 759.9, 779.9, 782.5, 785-785.3, 786.0-786.2, 786.4-786.5, 786.7, 786.9, 788.1-788.2, E986-E987

List of International Classification of Diseases (ICD) codes mapped to the Global Burden of Disease cause list for causes of death		
**Cause**	**ICD10**	**ICD9**
Garbage Code (GBD Level 4)	B16.9, B64, B82-B82.9, B83.9, C69, C69.9, C91.1, C91.4-C91.5, C91.7-C91.9, C92.7-C92.9, C93.2, C93.5-C93.7, C93.9, E12-E14.9, G00, G00.9-G02.8, G03.9, I37.9, I42-I42.0, I42.9, I51.5, I64-I64.9, I67, I67.8-I68, I68.8-I69, I69.4-I69.9, J07-J08, J15.9, J17-J19.6, J22-J29, J64-J64.9, P23, P23.5-P23.9, P37.3-P37.4, R73-R73.9, V87-V87.1, V87.4-V88.1, V88.4-V89.9, V99-V99.0, X84-X84.9, Y09-Y09.9, Y85-Y85.9	070.3, 084, 084.6, 194-194.0, 194.9, 204.1, 204.5-204.9, 205.8-205.9, 206.2-206.9, 238, 244, 244.9, 250-250.9, 289.8-289.9, 307.5, 320, 320.9, 357.2, 362.0, 425, 425.4, 425.9, 429.1, 436-437, 437.9-439.6, 482.9-483, 484, 484.8-486.9, 770.0, 790.2, E808-E829

Figure 1A

measure	location	sex	age	cause	metric	infectious syndrome	pathogen	year	value	upper	lower
Deaths	India	All	All Ages	All causes	Number	All infectious syndromes	KP	2019	134418	185596	93816
Deaths	China	All	All Ages	All causes	Number	All infectious syndromes	KP	2019	71785	110930	45486
Deaths	Nigeria	All	All Ages	All causes	Number	All infectious syndromes	KP	2019	45782	59000	34092
Deaths	Pakistan	All	All Ages	All causes	Number	All infectious syndromes	KP	2019	36277	49879	25336
Deaths	United States of America	All	All Ages	All causes	Number	All infectious syndromes	KP	2019	30250	42871	20899
Deaths	Brazil	All	All Ages	All causes	Number	All infectious syndromes	KP	2019	25169	33857	18489
Deaths	Indonesia	All	All Ages	All causes	Number	All infectious syndromes	KP	2019	23793	33389	16158
Deaths	Russian Federation	All	All Ages	All causes	Number	All infectious syndromes	KP	2019	17292	25027	11421
Deaths	Ethiopia	All	All Ages	All causes	Number	All infectious syndromes	KP	2019	16224	21044	12404
Deaths	Japan	All	All Ages	All causes	Number	All infectious syndromes	KP	2019	16203	21508	11939
Deaths	Bangladesh	All	All Ages	All causes	Number	All infectious syndromes	KP	2019	15891	22707	10892
Deaths	Democratic Republic of the Congo	All	All Ages	All causes	Number	All infectious syndromes	KP	2019	13636	18036	10211
Deaths	Mexico	All	All Ages	All causes	Number	All infectious syndromes	KP	2019	12873	18541	8516
Deaths	Philippines	All	All Ages	All causes	Number	All infectious syndromes	KP	2019	12326	16585	9178
Deaths	United Republic of Tanzania	All	All Ages	All causes	Number	All infectious syndromes	KP	2019	10007	13212	7540
Deaths	Germany	All	All Ages	All causes	Number	All infectious syndromes	KP	2019	9390	13194	6485
Deaths	Myanmar	All	All Ages	All causes	Number	All infectious syndromes	KP	2019	8316	11935	5704
Deaths	Egypt	All	All Ages	All causes	Number	All infectious syndromes	KP	2019	8244	12324	5078
Deaths	United Kingdom	All	All Ages	All causes	Number	All infectious syndromes	KP	2019	7747	10261	5771
Deaths	South Africa	All	All Ages	All causes	Number	All infectious syndromes	KP	2019	7482	9732	5632
Deaths	Kenya	All	All Ages	All causes	Number	All infectious syndromes	KP	2019	6925	8926	5352
Deaths	Afghanistan	All	All Ages	All causes	Number	All infectious syndromes	KP	2019	6904	9284	5018
Deaths	Mali	All	All Ages	All causes	Number	All infectious syndromes	KP	2019	6800	9201	4960
Deaths	Argentina	All	All Ages	All causes	Number	All infectious syndromes	KP	2019	6717	8671	5159
Deaths	Burkina Faso	All	All Ages	All causes	Number	All infectious syndromes	KP	2019	6596	8732	4993
Deaths	Thailand	All	All Ages	All causes	Number	All infectious syndromes	KP	2019	6557	9568	4254
Deaths	Niger	All	All Ages	All causes	Number	All infectious syndromes	KP	2019	6471	8563	4734
Deaths	Uganda	All	All Ages	All causes	Number	All infectious syndromes	KP	2019	6464	8555	4846
Deaths	France	All	All Ages	All causes	Number	All infectious syndromes	KP	2019	6366	8832	4483
Deaths	Viet Nam	All	All Ages	All causes	Number	All infectious syndromes	KP	2019	6290	9346	4157
Deaths	Mozambique	All	All Ages	All causes	Number	All infectious syndromes	KP	2019	5807	7594	4438
Deaths	Turkey	All	All Ages	All causes	Number	All infectious syndromes	KP	2019	5447	8265	3552
Deaths	Italy	All	All Ages	All causes	Number	All infectious syndromes	KP	2019	5345	7741	3560
Deaths	Côte d'Ivoire	All	All Ages	All causes	Number	All infectious syndromes	KP	2019	4876	6439	3605
Deaths	Ghana	All	All Ages	All causes	Number	All infectious syndromes	KP	2019	4865	6392	3651
Deaths	Ukraine	All	All Ages	All causes	Number	All infectious syndromes	KP	2019	4765	7170	2876
Deaths	Spain	All	All Ages	All causes	Number	All infectious syndromes	KP	2019	4725	6429	3392
Deaths	Somalia	All	All Ages	All causes	Number	All infectious syndromes	KP	2019	4720	6404	3411
Deaths	Cameroon	All	All Ages	All causes	Number	All infectious syndromes	KP	2019	4655	6143	3451
Deaths	Madagascar	All	All Ages	All causes	Number	All infectious syndromes	KP	2019	4353	5658	3224
Deaths	Chad	All	All Ages	All causes	Number	All infectious syndromes	KP	2019	4226	5529	3164
Deaths	Angola	All	All Ages	All causes	Number	All infectious syndromes	KP	2019	4170	5554	3030
Deaths	Nepal	All	All Ages	All causes	Number	All infectious syndromes	KP	2019	3933	5558	2745
Deaths	Poland	All	All Ages	All causes	Number	All infectious syndromes	KP	2019	3893	5832	2518
Deaths	Sudan	All	All Ages	All causes	Number	All infectious syndromes	KP	2019	3820	5512	2449
Deaths	Republic of Korea	All	All Ages	All causes	Number	All infectious syndromes	KP	2019	3737	5141	2660
Deaths	Colombia	All	All Ages	All causes	Number	All infectious syndromes	KP	2019	3475	5146	2246
Deaths	Peru	All	All Ages	All causes	Number	All infectious syndromes	KP	2019	3311	4697	2274
Deaths	Yemen	All	All Ages	All causes	Number	All infectious syndromes	KP	2019	3211	4697	2026
Deaths	Zimbabwe	All	All Ages	All causes	Number	All infectious syndromes	KP	2019	3132	4041	2421
Deaths	Malawi	All	All Ages	All causes	Number	All infectious syndromes	KP	2019	3069	3984	2316
Deaths	Iran (Islamic Republic of)	All	All Ages	All causes	Number	All infectious syndromes	KP	2019	3020	4476	1992
Deaths	Morocco	All	All Ages	All causes	Number	All infectious syndromes	KP	2019	3020	4492	1903
Deaths	Uzbekistan	All	All Ages	All causes	Number	All infectious syndromes	KP	2019	2934	4210	2042
Deaths	Guinea	All	All Ages	All causes	Number	All infectious syndromes	KP	2019	2860	3817	2073
Deaths	Zambia	All	All Ages	All causes	Number	All infectious syndromes	KP	2019	2852	3799	2139
Deaths	Cambodia	All	All Ages	All causes	Number	All infectious syndromes	KP	2019	2816	3752	2094
Deaths	Benin	All	All Ages	All causes	Number	All infectious syndromes	KP	2019	2786	3772	2000
Deaths	Romania	All	All Ages	All causes	Number	All infectious syndromes	KP	2019	2676	4031	1713
Deaths	Taiwan (Province of China)	All	All Ages	All causes	Number	All infectious syndromes	KP	2019	2649	3760	1847
Deaths	Canada	All	All Ages	All causes	Number	All infectious syndromes	KP	2019	2623	3669	1822
Deaths	Malaysia	All	All Ages	All causes	Number	All infectious syndromes	KP	2019	2458	3363	1734
Deaths	Senegal	All	All Ages	All causes	Number	All infectious syndromes	KP	2019	2457	3257	1799
Deaths	Democratic People's Republic of Korea	All	All Ages	All causes	Number	All infectious syndromes	KP	2019	2455	3643	1577
Deaths	Algeria	All	All Ages	All causes	Number	All infectious syndromes	KP	2019	2449	3698	1563
Deaths	Burundi	All	All Ages	All causes	Number	All infectious syndromes	KP	2019	2158	2886	1543
Deaths	Guatemala	All	All Ages	All causes	Number	All infectious syndromes	KP	2019	2133	2976	1467
Deaths	Venezuela (Bolivarian Republic of)	All	All Ages	All causes	Number	All infectious syndromes	KP	2019	2042	3108	1258
Deaths	Sierra Leone	All	All Ages	All causes	Number	All infectious syndromes	KP	2019	2013	2733	1467
Deaths	Bolivia (Plurinational State of)	All	All Ages	All causes	Number	All infectious syndromes	KP	2019	1975	2690	1388
Deaths	Iraq	All	All Ages	All causes	Number	All infectious syndromes	KP	2019	1929	2926	1203
Deaths	South Sudan	All	All Ages	All causes	Number	All infectious syndromes	KP	2019	1899	2507	1420

Deaths	Haiti	All	All Ages	All causes	Number	All infectious syndromes	KP	2019	1876	2564	1331
Deaths	Rwanda	All	All Ages	All causes	Number	All infectious syndromes	KP	2019	1817	2412	1363
Deaths	Ecuador	All	All Ages	All causes	Number	All infectious syndromes	KP	2019	1731	2478	1172
Deaths	Kazakhstan	All	All Ages	All causes	Number	All infectious syndromes	KP	2019	1730	2555	1140
Deaths	Netherlands	All	All Ages	All causes	Number	All infectious syndromes	KP	2019	1698	2286	1230
Deaths	Chile	All	All Ages	All causes	Number	All infectious syndromes	KP	2019	1691	2307	1214
Deaths	Central African Republic	All	All Ages	All causes	Number	All infectious syndromes	KP	2019	1566	2109	1138
Deaths	Portugal	All	All Ages	All causes	Number	All infectious syndromes	KP	2019	1529	2016	1151
Deaths	Australia	All	All Ages	All causes	Number	All infectious syndromes	KP	2019	1524	2147	1029
Deaths	Papua New Guinea	All	All Ages	All causes	Number	All infectious syndromes	KP	2019	1490	2056	1050
Deaths	Saudi Arabia	All	All Ages	All causes	Number	All infectious syndromes	KP	2019	1342	1999	889
Deaths	Belgium	All	All Ages	All causes	Number	All infectious syndromes	KP	2019	1329	1775	975
Deaths	Sri Lanka	All	All Ages	All causes	Number	All infectious syndromes	KP	2019	1241	1888	772
Deaths	Togo	All	All Ages	All causes	Number	All infectious syndromes	KP	2019	1217	1624	892
Deaths	Hungary	All	All Ages	All causes	Number	All infectious syndromes	KP	2019	1191	1797	735
Deaths	Bulgaria	All	All Ages	All causes	Number	All infectious syndromes	KP	2019	1183	1805	710
Deaths	Greece	All	All Ages	All causes	Number	All infectious syndromes	KP	2019	1183	1658	836
Deaths	Eritrea	All	All Ages	All causes	Number	All infectious syndromes	KP	2019	1170	1658	811
Deaths	Dominican Republic	All	All Ages	All causes	Number	All infectious syndromes	KP	2019	1099	1611	693
Deaths	Honduras	All	All Ages	All causes	Number	All infectious syndromes	KP	2019	1093	1552	723
Deaths	Serbia	All	All Ages	All causes	Number	All infectious syndromes	KP	2019	1072	1629	652
Deaths	Cuba	All	All Ages	All causes	Number	All infectious syndromes	KP	2019	994	1420	696
Deaths	Lao People's Democratic Republic	All	All Ages	All causes	Number	All infectious syndromes	KP	2019	991	1375	699
Deaths	Azerbaijan	All	All Ages	All causes	Number	All infectious syndromes	KP	2019	964	1416	639
Deaths	Czechia	All	All Ages	All causes	Number	All infectious syndromes	KP	2019	897	1306	581
Deaths	Syrian Arab Republic	All	All Ages	All causes	Number	All infectious syndromes	KP	2019	877	1312	565
Deaths	Sweden	All	All Ages	All causes	Number	All infectious syndromes	KP	2019	786	1109	546
Deaths	Belarus	All	All Ages	All causes	Number	All infectious syndromes	KP	2019	781	1239	475
Deaths	Liberia	All	All Ages	All causes	Number	All infectious syndromes	KP	2019	712	950	526
Deaths	Congo	All	All Ages	All causes	Number	All infectious syndromes	KP	2019	677	927	486
Deaths	Tajikistan	All	All Ages	All causes	Number	All infectious syndromes	KP	2019	669	947	466
Deaths	Switzerland	All	All Ages	All causes	Number	All infectious syndromes	KP	2019	661	904	460
Deaths	Austria	All	All Ages	All causes	Number	All infectious syndromes	KP	2019	660	972	431
Deaths	El Salvador	All	All Ages	All causes	Number	All infectious syndromes	KP	2019	657	959	420
Deaths	Denmark	All	All Ages	All causes	Number	All infectious syndromes	KP	2019	596	825	423
Deaths	Israel	All	All Ages	All causes	Number	All infectious syndromes	KP	2019	596	816	428
Deaths	Paraguay	All	All Ages	All causes	Number	All infectious syndromes	KP	2019	583	856	377
Deaths	Slovakia	All	All Ages	All causes	Number	All infectious syndromes	KP	2019	561	828	358
Deaths	Lesotho	All	All Ages	All causes	Number	All infectious syndromes	KP	2019	533	730	384
Deaths	Uruguay	All	All Ages	All causes	Number	All infectious syndromes	KP	2019	531	718	388
Deaths	Georgia	All	All Ages	All causes	Number	All infectious syndromes	KP	2019	511	771	312
Deaths	Nicaragua	All	All Ages	All causes	Number	All infectious syndromes	KP	2019	508	737	337
Deaths	Mauritania	All	All Ages	All causes	Number	All infectious syndromes	KP	2019	506	692	354
Deaths	Tunisia	All	All Ages	All causes	Number	All infectious syndromes	KP	2019	495	773	299
Deaths	Republic of Moldova	All	All Ages	All causes	Number	All infectious syndromes	KP	2019	481	710	317
Deaths	Turkmenistan	All	All Ages	All causes	Number	All infectious syndromes	KP	2019	480	706	313
Deaths	Kyrgyzstan	All	All Ages	All causes	Number	All infectious syndromes	KP	2019	440	653	281
Deaths	Finland	All	All Ages	All causes	Number	All infectious syndromes	KP	2019	435	633	287
Deaths	Mongolia	All	All Ages	All causes	Number	All infectious syndromes	KP	2019	424	627	279
Deaths	Puerto Rico	All	All Ages	All causes	Number	All infectious syndromes	KP	2019	421	620	274
Deaths	Norway	All	All Ages	All causes	Number	All infectious syndromes	KP	2019	403	546	297
Deaths	Croatia	All	All Ages	All causes	Number	All infectious syndromes	KP	2019	391	578	245
Deaths	Gambia	All	All Ages	All causes	Number	All infectious syndromes	KP	2019	391	509	295
Deaths	Guinea-Bissau	All	All Ages	All causes	Number	All infectious syndromes	KP	2019	385	510	286
Deaths	Libya	All	All Ages	All causes	Number	All infectious syndromes	KP	2019	378	583	234
Deaths	Armenia	All	All Ages	All causes	Number	All infectious syndromes	KP	2019	361	516	237
Deaths	Lithuania	All	All Ages	All causes	Number	All infectious syndromes	KP	2019	357	538	227
Deaths	Namibia	All	All Ages	All causes	Number	All infectious syndromes	KP	2019	356	490	255
Deaths	Costa Rica	All	All Ages	All causes	Number	All infectious syndromes	KP	2019	354	525	224
Deaths	Singapore	All	All Ages	All causes	Number	All infectious syndromes	KP	2019	350	429	287
Deaths	Botswana	All	All Ages	All causes	Number	All infectious syndromes	KP	2019	341	477	247
Deaths	Bosnia and Herzegovina	All	All Ages	All causes	Number	All infectious syndromes	KP	2019	327	504	191
Deaths	Jordan	All	All Ages	All causes	Number	All infectious syndromes	KP	2019	316	465	209
Deaths	Ireland	All	All Ages	All causes	Number	All infectious syndromes	KP	2019	315	430	226
Deaths	New Zealand	All	All Ages	All causes	Number	All infectious syndromes	KP	2019	301	434	204
Deaths	Panama	All	All Ages	All causes	Number	All infectious syndromes	KP	2019	290	428	186
Deaths	Lebanon	All	All Ages	All causes	Number	All infectious syndromes	KP	2019	285	428	186
Deaths	United Arab Emirates	All	All Ages	All causes	Number	All infectious syndromes	KP	2019	284	460	168
Deaths	Palestine	All	All Ages	All causes	Number	All infectious syndromes	KP	2019	243	356	162
Deaths	Latvia	All	All Ages	All causes	Number	All infectious syndromes	KP	2019	237	354	152
Deaths	Albania	All	All Ages	All causes	Number	All infectious syndromes	KP	2019	214	336	125
Deaths	Jamaica	All	All Ages	All causes	Number	All infectious syndromes	KP	2019	213	316	134
Deaths	Eswatini	All	All Ages	All causes	Number	All infectious syndromes	KP	2019	201	276	144
Deaths	Slovenia	All	All Ages	All causes	Number	All infectious syndromes	KP	2019	197	303	125
Deaths	North Macedonia	All	All Ages	All causes	Number	All infectious syndromes	KP	2019	184	287	107
Deaths	Djibouti	All	All Ages	All causes	Number	All infectious syndromes	KP	2019	173	238	124

Deaths	Gabon	All	All Ages	All causes	Number	All infectious syndromes	KP	2019	170	237	122
Deaths	Trinidad and Tobago	All	All Ages	All causes	Number	All infectious syndromes	KP	2019	163	248	99
Deaths	Timor-Leste	All	All Ages	All causes	Number	All infectious syndromes	KP	2019	136	184	98
Deaths	Mauritius	All	All Ages	All causes	Number	All infectious syndromes	KP	2019	136	205	83
Deaths	Oman	All	All Ages	All causes	Number	All infectious syndromes	KP	2019	117	172	78
Deaths	Fiji	All	All Ages	All causes	Number	All infectious syndromes	KP	2019	115	172	72
Deaths	Guyana	All	All Ages	All causes	Number	All infectious syndromes	KP	2019	112	164	72
Deaths	Kuwait	All	All Ages	All causes	Number	All infectious syndromes	KP	2019	112	160	79
Deaths	Solomon Islands	All	All Ages	All causes	Number	All infectious syndromes	KP	2019	111	151	83
Deaths	Estonia	All	All Ages	All causes	Number	All infectious syndromes	KP	2019	108	165	67
Deaths	Equatorial Guinea	All	All Ages	All causes	Number	All infectious syndromes	KP	2019	100	144	67
Deaths	Comoros	All	All Ages	All causes	Number	All infectious syndromes	KP	2019	100	132	74
Deaths	Cyprus	All	All Ages	All causes	Number	All infectious syndromes	KP	2019	88	125	62
Deaths	Bhutan	All	All Ages	All causes	Number	All infectious syndromes	KP	2019	64	95	43
Deaths	Suriname	All	All Ages	All causes	Number	All infectious syndromes	KP	2019	61	87	41
Deaths	Barbados	All	All Ages	All causes	Number	All infectious syndromes	KP	2019	52	72	37
Deaths	Bahrain	All	All Ages	All causes	Number	All infectious syndromes	KP	2019	51	79	32
Deaths	Montenegro	All	All Ages	All causes	Number	All infectious syndromes	KP	2019	46	70	28
Deaths	Cabo Verde	All	All Ages	All causes	Number	All infectious syndromes	KP	2019	46	63	34
Deaths	Qatar	All	All Ages	All causes	Number	All infectious syndromes	KP	2019	43	69	25
Deaths	Luxembourg	All	All Ages	All causes	Number	All infectious syndromes	KP	2019	41	58	28
Deaths	Bahamas	All	All Ages	All causes	Number	All infectious syndromes	KP	2019	39	56	26
Deaths	Malta	All	All Ages	All causes	Number	All infectious syndromes	KP	2019	36	49	26
Deaths	Vanuatu	All	All Ages	All causes	Number	All infectious syndromes	KP	2019	31	45	21
Deaths	Sao Tome and Principe	All	All Ages	All causes	Number	All infectious syndromes	KP	2019	26	35	19
Deaths	Belize	All	All Ages	All causes	Number	All infectious syndromes	KP	2019	25	35	17
Deaths	Kiribati	All	All Ages	All causes	Number	All infectious syndromes	KP	2019	20	29	14
Deaths	Brunei Darussalam	All	All Ages	All causes	Number	All infectious syndromes	KP	2019	20	28	13
Deaths	Iceland	All	All Ages	All causes	Number	All infectious syndromes	KP	2019	18	25	13
Deaths	Saint Lucia	All	All Ages	All causes	Number	All infectious syndromes	KP	2019	17	24	11
Deaths	Samoa	All	All Ages	All causes	Number	All infectious syndromes	KP	2019	16	23	10
Deaths	United States Virgin Islands	All	All Ages	All causes	Number	All infectious syndromes	KP	2019	16	23	11
Deaths	Tonga	All	All Ages	All causes	Number	All infectious syndromes	KP	2019	14	19	9
Deaths	Maldives	All	All Ages	All causes	Number	All infectious syndromes	KP	2019	13	19	8
Deaths	Micronesia (Federated States of)	All	All Ages	All causes	Number	All infectious syndromes	KP	2019	13	20	9
Deaths	Saint Vincent and the Grenadines	All	All Ages	All causes	Number	All infectious syndromes	KP	2019	12	17	8
Deaths	Seychelles	All	All Ages	All causes	Number	All infectious syndromes	KP	2019	12	16	9
Deaths	Grenada	All	All Ages	All causes	Number	All infectious syndromes	KP	2019	11	15	7
Deaths	Guam	All	All Ages	All causes	Number	All infectious syndromes	KP	2019	11	16	7
Deaths	Dominica	All	All Ages	All causes	Number	All infectious syndromes	KP	2019	9	13	6
Deaths	Marshall Islands	All	All Ages	All causes	Number	All infectious syndromes	KP	2019	7	10	5
Deaths	Antigua and Barbuda	All	All Ages	All causes	Number	All infectious syndromes	KP	2019	7	10	5
Deaths	Greenland	All	All Ages	All causes	Number	All infectious syndromes	KP	2019	7	10	4
Deaths	Andorra	All	All Ages	All causes	Number	All infectious syndromes	KP	2019	6	9	4
Deaths	Saint Kitts and Nevis	All	All Ages	All causes	Number	All infectious syndromes	KP	2019	6	8	4
Deaths	American Samoa	All	All Ages	All causes	Number	All infectious syndromes	KP	2019	5	7	3
Deaths	Monaco	All	All Ages	All causes	Number	All infectious syndromes	KP	2019	5	7	3
Deaths	Bermuda	All	All Ages	All causes	Number	All infectious syndromes	KP	2019	4	6	3
Deaths	Northern Mariana Islands	All	All Ages	All causes	Number	All infectious syndromes	KP	2019	4	5	2
Deaths	Palau	All	All Ages	All causes	Number	All infectious syndromes	KP	2019	3	5	2
Deaths	San Marino	All	All Ages	All causes	Number	All infectious syndromes	KP	2019	3	4	2
Deaths	Cook Islands	All	All Ages	All causes	Number	All infectious syndromes	KP	2019	2	2	1
Deaths	Nauru	All	All Ages	All causes	Number	All infectious syndromes	KP	2019	1	1	1
Deaths	Tuvalu	All	All Ages	All causes	Number	All infectious syndromes	KP	2019	1	2	1
Deaths	Niue	All	All Ages	All causes	Number	All infectious syndromes	KP	2019	0	0	0
Deaths	Tokelau	All	All Ages	All causes	Number	All infectious syndromes	KP	2019	0	0	0

Figure 1B

measure	location	sex	age	cause	metric	infectious syndrome	pathogen	year	value (per 100,000)	upper (per100,000)	lower (per100,000)
Deaths	Central African Republic	All	Age-standardized	All causes	Rate	All infectious syndromes	KP	2019	41.01	55.90	29.24
Deaths	Lesotho	All	Age-standardized	All causes	Rate	All infectious syndromes	KP	2019	40.09	55.26	28.56
Deaths	Guinea-Bissau	All	Age-standardized	All causes	Rate	All infectious syndromes	KP	2019	36.35	48.22	27.25
Deaths	Eritrea	All	Age-standardized	All causes	Rate	All infectious syndromes	KP	2019	35.90	50.98	25.35
Deaths	Zimbabwe	All	Age-standardized	All causes	Rate	All infectious syndromes	KP	2019	35.23	46.03	26.76
Deaths	Burkina Faso	All	Age-standardized	All causes	Rate	All infectious syndromes	KP	2019	35.04	45.12	26.92
Deaths	Solomon Islands	All	Age-standardized	All causes	Rate	All infectious syndromes	KP	2019	33.46	45.93	24.72
Deaths	Sierra Leone	All	Age-standardized	All causes	Rate	All infectious syndromes	KP	2019	32.33	44.08	23.67
Deaths	Mali	All	Age-standardized	All causes	Rate	All infectious syndromes	KP	2019	32.02	43.60	22.96
Deaths	Afghanistan	All	Age-standardized	All causes	Rate	All infectious syndromes	KP	2019	31.98	44.18	22.27
Deaths	Niger	All	Age-standardized	All causes	Rate	All infectious syndromes	KP	2019	31.85	41.98	23.63
Deaths	Gambia	All	Age-standardized	All causes	Rate	All infectious syndromes	KP	2019	31.42	40.73	23.77
Deaths	Kiribati	All	Age-standardized	All causes	Rate	All infectious syndromes	KP	2019	31.38	45.20	21.18
Deaths	Mozambique	All	Age-standardized	All causes	Rate	All infectious syndromes	KP	2019	31.08	41.12	23.15
Deaths	Burundi	All	Age-standardized	All causes	Rate	All infectious syndromes	KP	2019	30.87	41.04	22.48
Deaths	Somalia	All	Age-standardized	All causes	Rate	All infectious syndromes	KP	2019	30.83	42.81	21.98
Deaths	Eswatini	All	Age-standardized	All causes	Rate	All infectious syndromes	KP	2019	30.79	43.09	21.36
Deaths	Benin	All	Age-standardized	All causes	Rate	All infectious syndromes	KP	2019	29.40	39.24	21.55
Deaths	Zambia	All	Age-standardized	All causes	Rate	All infectious syndromes	KP	2019	29.29	39.31	21.68
Deaths	Malawi	All	Age-standardized	All causes	Rate	All infectious syndromes	KP	2019	28.84	37.33	21.92
Deaths	Chad	All	Age-standardized	All causes	Rate	All infectious syndromes	KP	2019	28.75	37.96	21.87
Deaths	Madagascar	All	Age-standardized	All causes	Rate	All infectious syndromes	KP	2019	28.63	38.35	20.50
Deaths	Guinea	All	Age-standardized	All causes	Rate	All infectious syndromes	KP	2019	28.30	37.77	20.66
Deaths	Côte d'Ivoire	All	Age-standardized	All causes	Rate	All infectious syndromes	KP	2019	28.29	37.60	21.23
Deaths	Democratic Republic of the Congo	All	Age-standardized	All causes	Rate	All infectious syndromes	KP	2019	28.16	38.12	20.63
Deaths	Kenya	All	Age-standardized	All causes	Rate	All infectious syndromes	KP	2019	28.04	36.73	21.60
Deaths	Togo	All	Age-standardized	All causes	Rate	All infectious syndromes	KP	2019	26.76	35.81	20.11
Deaths	Cameroon	All	Age-standardized	All causes	Rate	All infectious syndromes	KP	2019	26.68	35.70	19.56
Deaths	Nigeria	All	Age-standardized	All causes	Rate	All infectious syndromes	KP	2019	26.53	35.25	19.66
Deaths	Ghana	All	Age-standardized	All causes	Rate	All infectious syndromes	KP	2019	26.03	34.21	20.05
Deaths	Liberia	All	Age-standardized	All causes	Rate	All infectious syndromes	KP	2019	25.92	35.05	19.13
Deaths	Cambodia	All	Age-standardized	All causes	Rate	All infectious syndromes	KP	2019	25.69	34.09	19.18
Deaths	Ethiopia	All	Age-standardized	All causes	Rate	All infectious syndromes	KP	2019	25.64	32.97	19.55
Deaths	Uganda	All	Age-standardized	All causes	Rate	All infectious syndromes	KP	2019	25.64	33.64	19.21
Deaths	United Republic of Tanzania	All	Age-standardized	All causes	Rate	All infectious syndromes	KP	2019	25.63	33.82	19.40
Deaths	Rwanda	All	Age-standardized	All causes	Rate	All infectious syndromes	KP	2019	25.61	33.64	19.31
Deaths	Sao Tome and Principe	All	Age-standardized	All causes	Rate	All infectious syndromes	KP	2019	25.20	33.05	18.33
Deaths	Congo	All	Age-standardized	All causes	Rate	All infectious syndromes	KP	2019	25.02	34.17	18.11
Deaths	Senegal	All	Age-standardized	All causes	Rate	All infectious syndromes	KP	2019	24.77	33.64	18.19
Deaths	Botswana	All	Age-standardized	All causes	Rate	All infectious syndromes	KP	2019	24.51	34.46	17.69
Deaths	South Sudan	All	Age-standardized	All causes	Rate	All infectious syndromes	KP	2019	24.43	32.66	18.19
Deaths	Djibouti	All	Age-standardized	All causes	Rate	All infectious syndromes	KP	2019	24.18	33.17	17.53
Deaths	Angola	All	Age-standardized	All causes	Rate	All infectious syndromes	KP	2019	24.01	32.24	17.63
Deaths	Namibia	All	Age-standardized	All causes	Rate	All infectious syndromes	KP	2019	23.46	32.04	16.76
Deaths	Pakistan	All	Age-standardized	All causes	Rate	All infectious syndromes	KP	2019	23.36	32.48	15.75
Deaths	Micronesia (Federated States of)	All	Age-standardized	All causes	Rate	All infectious syndromes	KP	2019	23.21	33.57	15.51
Deaths	Marshall Islands	All	Age-standardized	All causes	Rate	All infectious syndromes	KP	2019	23.00	33.88	15.33
Deaths	Bolivia (Plurinational State of)	All	Age-standardized	All causes	Rate	All infectious syndromes	KP	2019	22.56	30.51	15.76
Deaths	Haiti	All	Age-standardized	All causes	Rate	All infectious syndromes	KP	2019	21.87	30.87	15.12
Deaths	Lao People's Democratic Republic	All	Age-standardized	All causes	Rate	All infectious syndromes	KP	2019	21.80	30.26	15.64
Deaths	Papua New Guinea	All	Age-standardized	All causes	Rate	All infectious syndromes	KP	2019	21.74	30.86	14.56
Deaths	Mongolia	All	Age-standardized	All causes	Rate	All infectious syndromes	KP	2019	20.38	29.92	13.32
Deaths	Mauritania	All	Age-standardized	All causes	Rate	All infectious syndromes	KP	2019	20.26	27.54	14.54
Deaths	Guyana	All	Age-standardized	All causes	Rate	All infectious syndromes	KP	2019	19.75	28.44	12.88
Deaths	Myanmar	All	Age-standardized	All causes	Rate	All infectious syndromes	KP	2019	19.70	28.42	13.49
Deaths	Nepal	All	Age-standardized	All causes	Rate	All infectious syndromes	KP	2019	19.54	28.01	13.51
Deaths	Comoros	All	Age-standardized	All causes	Rate	All infectious syndromes	KP	2019	19.31	25.91	14.36
Deaths	Palau	All	Age-standardized	All causes	Rate	All infectious syndromes	KP	2019	19.25	27.22	13.77
Deaths	Guatemala	All	Age-standardized	All causes	Rate	All infectious syndromes	KP	2019	18.98	26.43	13.29
Deaths	Fiji	All	Age-standardized	All causes	Rate	All infectious syndromes	KP	2019	18.77	27.71	11.91
Deaths	Honduras	All	Age-standardized	All causes	Rate	All infectious syndromes	KP	2019	18.75	26.46	12.43
Deaths	Vanuatu	All	Age-standardized	All causes	Rate	All infectious syndromes	KP	2019	18.16	26.41	11.94
Deaths	South Africa	All	Age-standardized	All causes	Rate	All infectious syndromes	KP	2019	17.76	23.28	13.27
Deaths	Philippines	All	Age-standardized	All causes	Rate	All infectious syndromes	KP	2019	17.02	22.92	12.68
Deaths	Yemen	All	Age-standardized	All causes	Rate	All infectious syndromes	KP	2019	16.93	25.11	10.97
Deaths	Tonga	All	Age-standardized	All causes	Rate	All infectious syndromes	KP	2019	16.75	23.38	11.40
Deaths	Nauru	All	Age-standardized	All causes	Rate	All infectious syndromes	KP	2019	16.72	24.39	11.48
Deaths	Gabon	All	Age-standardized	All causes	Rate	All infectious syndromes	KP	2019	16.45	22.63	11.91
Deaths	Equatorial Guinea	All	Age-standardized	All causes	Rate	All infectious syndromes	KP	2019	15.73	22.45	10.76
Deaths	Egypt	All	Age-standardized	All causes	Rate	All infectious syndromes	KP	2019	15.64	23.13	9.62
Deaths	Timor-Leste	All	Age-standardized	All causes	Rate	All infectious syndromes	KP	2019	15.47	21.53	11.00
Deaths	Uzbekistan	All	Age-standardized	All causes	Rate	All infectious syndromes	KP	2019	15.41	22.68	10.25
Deaths	Sudan	All	Age-standardized	All causes	Rate	All infectious syndromes	KP	2019	14.81	21.36	9.51
Deaths	Tuvalu	All	Age-standardized	All causes	Rate	All infectious syndromes	KP	2019	14.14	21.18	9.12
Deaths	Indonesia	All	Age-standardized	All causes	Rate	All infectious syndromes	KP	2019	14.03	19.63	9.57

Deaths	Azerbaijan	All	Age-standardized	All causes	Rate	All infectious syndromes	KP	2019	13.68	20.10	9.00
Deaths	Bangladesh	All	Age-standardized	All causes	Rate	All infectious syndromes	KP	2019	13.47	19.44	9.22
Deaths	Niue	All	Age-standardized	All causes	Rate	All infectious syndromes	KP	2019	13.43	19.27	8.93
Deaths	India	All	Age-standardized	All causes	Rate	All infectious syndromes	KP	2019	13.17	18.31	9.15
Deaths	Seychelles	All	Age-standardized	All causes	Rate	All infectious syndromes	KP	2019	12.77	16.81	9.61
Deaths	Tajikistan	All	Age-standardized	All causes	Rate	All infectious syndromes	KP	2019	12.62	18.50	8.47
Deaths	Ecuador	All	Age-standardized	All causes	Rate	All infectious syndromes	KP	2019	12.46	17.87	8.48
Deaths	Argentina	All	Age-standardized	All causes	Rate	All infectious syndromes	KP	2019	12.44	16.10	9.54
Deaths	United Arab Emirates	All	Age-standardized	All causes	Rate	All infectious syndromes	KP	2019	12.27	17.50	8.55
Deaths	Nicaragua	All	Age-standardized	All causes	Rate	All infectious syndromes	KP	2019	12.17	17.75	8.03
Deaths	Bhutan	All	Age-standardized	All causes	Rate	All infectious syndromes	KP	2019	12.15	17.96	8.16
Deaths	Dominican Republic	All	Age-standardized	All causes	Rate	All infectious syndromes	KP	2019	11.81	17.38	7.44
Deaths	Samoa	All	Age-standardized	All causes	Rate	All infectious syndromes	KP	2019	11.76	17.01	7.75
Deaths	Turkmenistan	All	Age-standardized	All causes	Rate	All infectious syndromes	KP	2019	11.71	17.59	7.54
Deaths	Brazil	All	Age-standardized	All causes	Rate	All infectious syndromes	KP	2019	11.65	15.57	8.62
Deaths	Mexico	All	Age-standardized	All causes	Rate	All infectious syndromes	KP	2019	11.64	16.73	7.76
Deaths	Greenland	All	Age-standardized	All causes	Rate	All infectious syndromes	KP	2019	11.63	16.63	7.56
Deaths	Morocco	All	Age-standardized	All causes	Rate	All infectious syndromes	KP	2019	11.62	17.11	7.37
Deaths	Barbados	All	Age-standardized	All causes	Rate	All infectious syndromes	KP	2019	11.43	15.77	7.96
Deaths	Suriname	All	Age-standardized	All causes	Rate	All infectious syndromes	KP	2019	11.33	16.00	7.61
Deaths	Bahamas	All	Age-standardized	All causes	Rate	All infectious syndromes	KP	2019	11.26	16.28	7.68
Deaths	Dominica	All	Age-standardized	All causes	Rate	All infectious syndromes	KP	2019	11.04	15.90	7.17
Deaths	American Samoa	All	Age-standardized	All causes	Rate	All infectious syndromes	KP	2019	11.01	15.84	7.29
Deaths	Grenada	All	Age-standardized	All causes	Rate	All infectious syndromes	KP	2019	10.96	15.12	7.74
Deaths	Kazakhstan	All	Age-standardized	All causes	Rate	All infectious syndromes	KP	2019	10.91	16.24	7.08
Deaths	Palestine	All	Age-standardized	All causes	Rate	All infectious syndromes	KP	2019	10.85	16.05	7.24
Deaths	Malaysia	All	Age-standardized	All causes	Rate	All infectious syndromes	KP	2019	10.76	14.54	7.66
Deaths	Cabo Verde	All	Age-standardized	All causes	Rate	All infectious syndromes	KP	2019	10.75	14.65	7.81
Deaths	Paraguay	All	Age-standardized	All causes	Rate	All infectious syndromes	KP	2019	10.71	15.64	6.96
Deaths	El Salvador	All	Age-standardized	All causes	Rate	All infectious syndromes	KP	2019	10.59	15.54	6.81
Deaths	Peru	All	Age-standardized	All causes	Rate	All infectious syndromes	KP	2019	10.22	14.50	7.00
Deaths	Saint Vincent and the Grenadines	All	Age-standardized	All causes	Rate	All infectious syndromes	KP	2019	10.18	14.47	6.85
Deaths	Saint Kitts and Nevis	All	Age-standardized	All causes	Rate	All infectious syndromes	KP	2019	10.00	13.99	6.95
Deaths	Brunei Darussalam	All	Age-standardized	All causes	Rate	All infectious syndromes	KP	2019	9.95	13.53	7.19
Deaths	Tokelau	All	Age-standardized	All causes	Rate	All infectious syndromes	KP	2019	9.91	14.61	6.40
Deaths	Trinidad and Tobago	All	Age-standardized	All causes	Rate	All infectious syndromes	KP	2019	9.81	14.96	5.96
Deaths	Bahrain	All	Age-standardized	All causes	Rate	All infectious syndromes	KP	2019	9.63	14.43	6.33
Deaths	Qatar	All	Age-standardized	All causes	Rate	All infectious syndromes	KP	2019	9.61	14.67	6.13
Deaths	Saudi Arabia	All	Age-standardized	All causes	Rate	All infectious syndromes	KP	2019	9.61	13.79	6.66
Deaths	Armenia	All	Age-standardized	All causes	Rate	All infectious syndromes	KP	2019	9.57	13.59	6.37
Deaths	United States Virgin Islands	All	Age-standardized	All causes	Rate	All infectious syndromes	KP	2019	9.54	13.69	6.37
Deaths	Kyrgyzstan	All	Age-standardized	All causes	Rate	All infectious syndromes	KP	2019	9.46	14.26	5.97
Deaths	Belize	All	Age-standardized	All causes	Rate	All infectious syndromes	KP	2019	9.37	13.05	6.51
Deaths	Oman	All	Age-standardized	All causes	Rate	All infectious syndromes	KP	2019	9.25	13.35	6.47
Deaths	Republic of Moldova	All	Age-standardized	All causes	Rate	All infectious syndromes	KP	2019	9.16	13.35	6.09
Deaths	Uruguay	All	Age-standardized	All causes	Rate	All infectious syndromes	KP	2019	9.05	12.29	6.54
Deaths	Northern Mariana Islands	All	Age-standardized	All causes	Rate	All infectious syndromes	KP	2019	8.98	12.91	6.05
Deaths	Syrian Arab Republic	All	Age-standardized	All causes	Rate	All infectious syndromes	KP	2019	8.95	13.26	5.78
Deaths	Mauritius	All	Age-standardized	All causes	Rate	All infectious syndromes	KP	2019	8.86	13.35	5.51
Deaths	Georgia	All	Age-standardized	All causes	Rate	All infectious syndromes	KP	2019	8.82	13.20	5.39
Deaths	Algeria	All	Age-standardized	All causes	Rate	All infectious syndromes	KP	2019	8.71	13.06	5.67
Deaths	Bulgaria	All	Age-standardized	All causes	Rate	All infectious syndromes	KP	2019	8.68	13.23	5.19
Deaths	Democratic People's Republic of Korea	All	Age-standardized	All causes	Rate	All infectious syndromes	KP	2019	8.67	12.79	5.64
Deaths	Saint Lucia	All	Age-standardized	All causes	Rate	All infectious syndromes	KP	2019	8.60	12.37	5.67
Deaths	Iraq	All	Age-standardized	All causes	Rate	All infectious syndromes	KP	2019	8.34	12.70	5.14
Deaths	Libya	All	Age-standardized	All causes	Rate	All infectious syndromes	KP	2019	8.26	12.56	5.16
Deaths	Viet Nam	All	Age-standardized	All causes	Rate	All infectious syndromes	KP	2019	7.86	11.55	5.29
Deaths	Russian Federation	All	Age-standardized	All causes	Rate	All infectious syndromes	KP	2019	7.85	11.38	5.19
Deaths	Antigua and Barbuda	All	Age-standardized	All causes	Rate	All infectious syndromes	KP	2019	7.85	11.03	5.39
Deaths	Romania	All	Age-standardized	All causes	Rate	All infectious syndromes	KP	2019	7.63	11.44	4.89
Deaths	Venezuela (Bolivarian Republic of)	All	Age-standardized	All causes	Rate	All infectious syndromes	KP	2019	7.49	11.37	4.65
Deaths	Chile	All	Age-standardized	All causes	Rate	All infectious syndromes	KP	2019	7.27	9.92	5.22
Deaths	Jamaica	All	Age-standardized	All causes	Rate	All infectious syndromes	KP	2019	7.17	10.67	4.46
Deaths	Serbia	All	Age-standardized	All causes	Rate	All infectious syndromes	KP	2019	7.15	10.95	4.31
Deaths	Costa Rica	All	Age-standardized	All causes	Rate	All infectious syndromes	KP	2019	7.01	10.39	4.42
Deaths	Panama	All	Age-standardized	All causes	Rate	All infectious syndromes	KP	2019	6.95	10.25	4.46
Deaths	Ukraine	All	Age-standardized	All causes	Rate	All infectious syndromes	KP	2019	6.94	10.40	4.20
Deaths	Turkey	All	Age-standardized	All causes	Rate	All infectious syndromes	KP	2019	6.94	10.46	4.53
Deaths	Thailand	All	Age-standardized	All causes	Rate	All infectious syndromes	KP	2019	6.87	9.90	4.50
Deaths	Taiwan (Province of China)	All	Age-standardized	All causes	Rate	All infectious syndromes	KP	2019	6.76	9.70	4.69
Deaths	North Macedonia	All	Age-standardized	All causes	Rate	All infectious syndromes	KP	2019	6.73	10.52	3.92
Deaths	Colombia	All	Age-standardized	All causes	Rate	All infectious syndromes	KP	2019	6.68	9.85	4.31
Deaths	Cook Islands	All	Age-standardized	All causes	Rate	All infectious syndromes	KP	2019	6.60	9.36	4.46
Deaths	Slovakia	All	Age-standardized	All causes	Rate	All infectious syndromes	KP	2019	6.44	9.53	4.08
Deaths	Lithuania	All	Age-standardized	All causes	Rate	All infectious syndromes	KP	2019	6.35	9.59	4.03
Deaths	Hungary	All	Age-standardized	All causes	Rate	All infectious syndromes	KP	2019	6.32	9.57	3.86
Deaths	Guam	All	Age-standardized	All causes	Rate	All infectious syndromes	KP	2019	6.10	8.77	3.97
Deaths	Latvia	All	Age-standardized	All causes	Rate	All infectious syndromes	KP	2019	6.06	9.10	3.86
Deaths	Bosnia and Herzegovina	All	Age-standardized	All causes	Rate	All infectious syndromes	KP	2019	5.91	9.23	3.44
Deaths	Puerto Rico	All	Age-standardized	All causes	Rate	All infectious syndromes	KP	2019	5.88	8.79	3.75

Deaths	United Kingdom	All	Age-standardized	All causes	Rate	All infectious syndromes	KP	2019	5.72	7.69	4.20
Deaths	Portugal	All	Age-standardized	All causes	Rate	All infectious syndromes	KP	2019	5.67	7.58	4.20
Deaths	Poland	All	Age-standardized	All causes	Rate	All infectious syndromes	KP	2019	5.66	8.56	3.62
Deaths	Sri Lanka	All	Age-standardized	All causes	Rate	All infectious syndromes	KP	2019	5.62	8.57	3.52
Deaths	Lebanon	All	Age-standardized	All causes	Rate	All infectious syndromes	KP	2019	5.57	8.42	3.64
Deaths	Albania	All	Age-standardized	All causes	Rate	All infectious syndromes	KP	2019	5.53	8.59	3.30
Deaths	Jordan	All	Age-standardized	All causes	Rate	All infectious syndromes	KP	2019	5.40	7.94	3.61
Deaths	United States of America	All	Age-standardized	All causes	Rate	All infectious syndromes	KP	2019	5.34	7.61	3.65
Deaths	Kuwait	All	Age-standardized	All causes	Rate	All infectious syndromes	KP	2019	5.32	7.46	3.83
Deaths	Cyprus	All	Age-standardized	All causes	Rate	All infectious syndromes	KP	2019	5.24	7.41	3.70
Deaths	Cuba	All	Age-standardized	All causes	Rate	All infectious syndromes	KP	2019	5.20	7.43	3.61
Deaths	Belarus	All	Age-standardized	All causes	Rate	All infectious syndromes	KP	2019	5.12	8.12	3.12
Deaths	Belgium	All	Age-standardized	All causes	Rate	All infectious syndromes	KP	2019	5.08	6.91	3.64
Deaths	Montenegro	All	Age-standardized	All causes	Rate	All infectious syndromes	KP	2019	4.97	7.65	3.00
Deaths	Denmark	All	Age-standardized	All causes	Rate	All infectious syndromes	KP	2019	4.94	6.91	3.46
Deaths	Singapore	All	Age-standardized	All causes	Rate	All infectious syndromes	KP	2019	4.93	6.03	4.05
Deaths	Israel	All	Age-standardized	All causes	Rate	All infectious syndromes	KP	2019	4.81	6.62	3.42
Deaths	Monaco	All	Age-standardized	All causes	Rate	All infectious syndromes	KP	2019	4.74	6.93	3.13
Deaths	Netherlands	All	Age-standardized	All causes	Rate	All infectious syndromes	KP	2019	4.73	6.44	3.39
Deaths	Iran (Islamic Republic of)	All	Age-standardized	All causes	Rate	All infectious syndromes	KP	2019	4.66	6.91	3.08
Deaths	Maldives	All	Age-standardized	All causes	Rate	All infectious syndromes	KP	2019	4.65	6.90	3.05
Deaths	Germany	All	Age-standardized	All causes	Rate	All infectious syndromes	KP	2019	4.58	6.54	3.11
Deaths	Republic of Korea	All	Age-standardized	All causes	Rate	All infectious syndromes	KP	2019	4.54	6.26	3.21
Deaths	Tunisia	All	Age-standardized	All causes	Rate	All infectious syndromes	KP	2019	4.50	6.94	2.71
Deaths	Croatia	All	Age-standardized	All causes	Rate	All infectious syndromes	KP	2019	4.40	6.59	2.73
Deaths	Czechia	All	Age-standardized	All causes	Rate	All infectious syndromes	KP	2019	4.31	6.30	2.78
Deaths	Slovenia	All	Age-standardized	All causes	Rate	All infectious syndromes	KP	2019	4.30	6.72	2.70
Deaths	Greece	All	Age-standardized	All causes	Rate	All infectious syndromes	KP	2019	4.30	6.15	2.95
Deaths	Spain	All	Age-standardized	All causes	Rate	All infectious syndromes	KP	2019	4.23	5.83	3.00
Deaths	China	All	Age-standardized	All causes	Rate	All infectious syndromes	KP	2019	4.22	6.48	2.71
Deaths	Andorra	All	Age-standardized	All causes	Rate	All infectious syndromes	KP	2019	4.22	6.41	2.74
Deaths	Ireland	All	Age-standardized	All causes	Rate	All infectious syndromes	KP	2019	4.15	5.71	2.96
Deaths	Estonia	All	Age-standardized	All causes	Rate	All infectious syndromes	KP	2019	4.14	6.38	2.57
Deaths	France	All	Age-standardized	All causes	Rate	All infectious syndromes	KP	2019	4.04	5.70	2.77
Deaths	Luxembourg	All	Age-standardized	All causes	Rate	All infectious syndromes	KP	2019	3.84	5.48	2.57
Deaths	San Marino	All	Age-standardized	All causes	Rate	All infectious syndromes	KP	2019	3.79	6.27	2.09
Deaths	Malta	All	Age-standardized	All causes	Rate	All infectious syndromes	KP	2019	3.75	5.20	2.66
Deaths	Norway	All	Age-standardized	All causes	Rate	All infectious syndromes	KP	2019	3.73	5.15	2.68
Deaths	New Zealand	All	Age-standardized	All causes	Rate	All infectious syndromes	KP	2019	3.70	5.39	2.48
Deaths	Canada	All	Age-standardized	All causes	Rate	All infectious syndromes	KP	2019	3.67	5.19	2.52
Deaths	Japan	All	Age-standardized	All causes	Rate	All infectious syndromes	KP	2019	3.63	4.90	2.63
Deaths	Austria	All	Age-standardized	All causes	Rate	All infectious syndromes	KP	2019	3.50	5.18	2.26
Deaths	Australia	All	Age-standardized	All causes	Rate	All infectious syndromes	KP	2019	3.44	4.91	2.31
Deaths	Finland	All	Age-standardized	All causes	Rate	All infectious syndromes	KP	2019	3.44	5.05	2.23
Deaths	Sweden	All	Age-standardized	All causes	Rate	All infectious syndromes	KP	2019	3.36	4.79	2.29
Deaths	Italy	All	Age-standardized	All causes	Rate	All infectious syndromes	KP	2019	3.35	4.92	2.20
Deaths	Switzerland	All	Age-standardized	All causes	Rate	All infectious syndromes	KP	2019	3.34	4.65	2.30
Deaths	Bermuda	All	Age-standardized	All causes	Rate	All infectious syndromes	KP	2019	3.16	4.68	2.04
Deaths	Iceland	All	Age-standardized	All causes	Rate	All infectious syndromes	KP	2019	2.97	4.15	2.04

Figure 2

measure	location	sex	age	cause	year	metric	infectious syndrome	pathogen	counterfactual	value	upper	lower
Deaths	Global	Both	All Ages	All causes	2019	Number	Bacterial infections ofthe skin and subcutaneous systems	KP	Drug-susceptible infection	1614	5952	229
Deaths	Global	Both	All Ages	All causes	2019	Number	Bacterial infections ofthe skin and subcutaneous systems	KP	No infection	5343	19802	773
Deaths	Global	Both	All Ages	All causes	2019	Number	Bloodstream infections	KP	Drug-susceptible infection	64308	104487	36134
Deaths	Global	Both	All Ages	All causes	2019	Number	Bloodstream infections	KP	No infection	214528	335332	128753
Deaths	Global	Both	All Ages	All causes	2019	Number	Endocarditis and other cardiac infections	KP	Drug-susceptible infection	2271	3316	1525
Deaths	Global	Both	All Ages	All causes	2019	Number	Endocarditis and other cardiac infections	KP	No infection	7777	10805	5707
Deaths	Global	Both	All Ages	All causes	2019	Number	Infections of bones, joints, and related organs	KP	Drug-susceptible infection	349	861	93
Deaths	Global	Both	All Ages	All causes	2019	Number	Infections of bones, joints, and related organs	KP	No infection	1121	2664	312
Deaths	Global	Both	All Ages	All causes	2019	Number	LRIs and all related infections in the thorax	KP	Drug-susceptible infection	69295	93725	50546
Deaths	Global	Both	All Ages	All causes	2019	Number	LRIs and all related infections in the thorax	KP	No infection	231776	288677	184551
Deaths	Global	Both	All Ages	All causes	2019	Number	Meningitis and other bacterial CNS infections	KP	Drug-susceptible infection	8974	13636	5847
Deaths	Global	Both	All Ages	All causes	2019	Number	Meningitis and other bacterial CNS infections	KP	No infection	30318	42755	21378
Deaths	Global	Both	All Ages	All causes	2019	Number	Peritoneal and intra-abdominal infections	KP	Drug-susceptible infection	37447	57239	23574
Deaths	Global	Both	All Ages	All causes	2019	Number	Peritoneal and intra-abdominal infections	KP	No infection	123158	182226	80452
Deaths	Global	Both	All Ages	All causes	2019	Number	Urinary tract infections and pyelonephritis	KP	Drug-susceptible infection	8334	12475	5477
Deaths	Global	Both	All Ages	All causes	2019	Number	Urinary tract infections and pyelonephritis	KP	No infection	27682	40307	19015
Deaths	Southeast Asia, East Asia, and Oceania	Both	All Ages	All causes	2019	Number	Bacterial infections ofthe skin and subcutaneous systems	KP	Drug-susceptible infection	390	1380	53
Deaths	Southeast Asia, East Asia, and Oceania	Both	All Ages	All causes	2019	Number	Bacterial infections ofthe skin and subcutaneous systems	KP	No infection	1353	4801	184
Deaths	Southeast Asia, East Asia, and Oceania	Both	All Ages	All causes	2019	Number	Bloodstream infections	KP	Drug-susceptible infection	10968	18921	5578
Deaths	Southeast Asia, East Asia, and Oceania	Both	All Ages	All causes	2019	Number	Bloodstream infections	KP	No infection	38012	62435	21412
Deaths	Southeast Asia, East Asia, and Oceania	Both	All Ages	All causes	2019	Number	Endocarditis and other cardiac infections	KP	Drug-susceptible infection	376	601	233
Deaths	Southeast Asia, East Asia, and Oceania	Both	All Ages	All causes	2019	Number	Endocarditis and other cardiac infections	KP	No infection	1292	1943	871
Deaths	Southeast Asia, East Asia, and Oceania	Both	All Ages	All causes	2019	Number	Infections of bones, joints, and related organs	KP	Drug-susceptible infection	59	149	16
Deaths	Southeast Asia, East Asia, and Oceania	Both	All Ages	All causes	2019	Number	Infections of bones, joints, and related organs	KP	No infection	204	484	60
Deaths	Southeast Asia, East Asia, and Oceania	Both	All Ages	All causes	2019	Number	LRIs and all related infections in the thorax	KP	Drug-susceptible infection	8237	12035	5386
Deaths	Southeast Asia, East Asia, and Oceania	Both	All Ages	All causes	2019	Number	LRIs and all related infections in the thorax	KP	No infection	28066	36996	20873
Deaths	Southeast Asia, East Asia, and Oceania	Both	All Ages	All causes	2019	Number	Meningitis and other bacterial CNS infections	KP	Drug-susceptible infection	621	1070	376
Deaths	Southeast Asia, East Asia, and Oceania	Both	All Ages	All causes	2019	Number	Meningitis and other bacterial CNS infections	KP	No infection	2126	3497	1411
Deaths	Southeast Asia, East Asia, and Oceania	Both	All Ages	All causes	2019	Number	Peritoneal and intra-abdominal infections	KP	Drug-susceptible infection	7687	12676	4376
Deaths	Southeast Asia, East Asia, and Oceania	Both	All Ages	All causes	2019	Number	Peritoneal and intra-abdominal infections	KP	No infection	26498	40532	16635
Deaths	Southeast Asia, East Asia, and Oceania	Both	All Ages	All causes	2019	Number	Urinary tract infections and pyelonephritis	KP	Drug-susceptible infection	1756	3187	879
Deaths	Southeast Asia, East Asia, and Oceania	Both	All Ages	All causes	2019	Number	Urinary tract infections and pyelonephritis	KP	No infection	6118	10565	3246
Deaths	Central Europe, Eastern Europe, and Central Asia	Both	All Ages	All causes	2019	Number	Bacterial infections ofthe skin and subcutaneous systems	KP	Drug-susceptible infection	133	474	20
Deaths	Central Europe, Eastern Europe, and Central Asia	Both	All Ages	All causes	2019	Number	Bacterial infections ofthe skin and subcutaneous systems	KP	No infection	424	1493	61
Deaths	Central Europe, Eastern Europe, and Central Asia	Both	All Ages	All causes	2019	Number	Bloodstream infections	KP	Drug-susceptible infection	4890	8473	2541
Deaths	Central Europe, Eastern Europe, and Central Asia	Both	All Ages	All causes	2019	Number	Bloodstream infections	KP	No infection	15789	26737	8492
Deaths	Central Europe, Eastern Europe, and Central Asia	Both	All Ages	All causes	2019	Number	Endocarditis and other cardiac infections	KP	Drug-susceptible infection	329	558	185
Deaths	Central Europe, Eastern Europe, and Central Asia	Both	All Ages	All causes	2019	Number	Endocarditis and other cardiac infections	KP	No infection	1086	1795	627
Deaths	Central Europe, Eastern Europe, and Central Asia	Both	All Ages	All causes	2019	Number	Infections of bones, joints, and related organs	KP	Drug-susceptible infection	18	44	5
Deaths	Central Europe, Eastern Europe, and Central Asia	Both	All Ages	All causes	2019	Number	Infections of bones, joints, and related organs	KP	No infection	58	139	17
Deaths	Central Europe, Eastern Europe, and Central Asia	Both	All Ages	All causes	2019	Number	LRIs and all related infections in the thorax	KP	Drug-susceptible infection	2580	3643	1804
Deaths	Central Europe, Eastern Europe, and Central Asia	Both	All Ages	All causes	2019	Number	LRIs and all related infections in the thorax	KP	No infection	8403	11258	6382
Deaths	Central Europe, Eastern Europe, and Central Asia	Both	All Ages	All causes	2019	Number	Meningitis and other bacterial CNS infections	KP	Drug-susceptible infection	134	239	82
Deaths	Central Europe, Eastern Europe, and Central Asia	Both	All Ages	All causes	2019	Number	Meningitis and other bacterial CNS infections	KP	No infection	436	774	279
Deaths	Central Europe, Eastern Europe, and Central Asia	Both	All Ages	All causes	2019	Number	Peritoneal and intra-abdominal infections	KP	Drug-susceptible infection	3534	5371	2231
Deaths	Central Europe, Eastern Europe, and Central Asia	Both	All Ages	All causes	2019	Number	Peritoneal and intra-abdominal infections	KP	No infection	11457	16709	7413
Deaths	Central Europe, Eastern Europe, and Central Asia	Both	All Ages	All causes	2019	Number	Urinary tract infections and pyelonephritis	KP	Drug-susceptible infection	1025	1597	639
Deaths	Central Europe, Eastern Europe, and Central Asia	Both	All Ages	All causes	2019	Number	Urinary tract infections and pyelonephritis	KP	No infection	3285	4988	2137
Deaths	High-income	Both	All Ages	All causes	2019	Number	Bacterial infections ofthe skin and subcutaneous systems	KP	Drug-susceptible infection	154	508	24
Deaths	High-income	Both	All Ages	All causes	2019	Number	Bacterial infections ofthe skin and subcutaneous systems	KP	No infection	620	2042	98
Deaths	High-income	Both	All Ages	All causes	2019	Number	Bloodstream infections	KP	Drug-susceptible infection	3960	6863	2042
Deaths	High-income	Both	All Ages	All causes	2019	Number	Bloodstream infections	KP	No infection	15879	26778	8609
Deaths	High-income	Both	All Ages	All causes	2019	Number	Endocarditis and other cardiac infections	KP	Drug-susceptible infection	481	706	279
Deaths	High-income	Both	All Ages	All causes	2019	Number	Endocarditis and other cardiac infections	KP	No infection	1952	2644	1255
Deaths	High-income	Both	All Ages	All causes	2019	Number	Infections of bones, joints, and related organs	KP	Drug-susceptible infection	17	39	5
Deaths	High-income	Both	All Ages	All causes	2019	Number	Infections of bones, joints, and related organs	KP	No infection	67	152	21

Deaths	High-income	Both	All Ages	All causes	2019	Number	LRIs and all related infections in the thorax	KP	Drug-susceptible infection	3209	4409	2314
Deaths	High-income	Both	All Ages	All causes	2019	Number	LRIs and all related infections in the thorax	KP	No infection	12914	15845	10529
Deaths	High-income	Both	All Ages	All causes	2019	Number	Meningitis and other bacterial CNS infections	KP	Drug-susceptible infection	76	136	46
Deaths	High-income	Both	All Ages	All causes	2019	Number	Meningitis and other bacterial CNS infections	KP	No infection	301	533	198
Deaths	High-income	Both	All Ages	All causes	2019	Number	Peritoneal and intra-abdominal infections	KP	Drug-susceptible infection	3230	4908	1933
Deaths	High-income	Both	All Ages	All causes	2019	Number	Peritoneal and intra-abdominal infections	KP	No infection	13047	19071	8642
Deaths	High-income	Both	All Ages	All causes	2019	Number	Urinary tract infections and pyelonephritis	KP	Drug-susceptible infection	1296	1837	885
Deaths	High-income	Both	All Ages	All causes	2019	Number	Urinary tract infections and pyelonephritis	KP	No infection	5279	7032	3993
Deaths	Latin America and Caribbean	Both	All Ages	All causes	2019	Number	Bacterial infections ofthe skin and subcutaneous systems	KP	Drug-susceptible infection	139	475	22
Deaths	Latin America and Caribbean	Both	All Ages	All causes	2019	Number	Bacterial infections ofthe skin and subcutaneous systems	KP	No infection	494	1645	81
Deaths	Latin America and Caribbean	Both	All Ages	All causes	2019	Number	Bloodstream infections	KP	Drug-susceptible infection	4479	7457	2411
Deaths	Latin America and Caribbean	Both	All Ages	All causes	2019	Number	Bloodstream infections	KP	No infection	16009	25541	9217
Deaths	Latin America and Caribbean	Both	All Ages	All causes	2019	Number	Endocarditis and other cardiac infections	KP	Drug-susceptible infection	175	242	122
Deaths	Latin America and Caribbean	Both	All Ages	All causes	2019	Number	Endocarditis and other cardiac infections	KP	No infection	612	785	468
Deaths	Latin America and Caribbean	Both	All Ages	All causes	2019	Number	Infections of bones, joints, and related organs	KP	Drug-susceptible infection	20	49	5
Deaths	Latin America and Caribbean	Both	All Ages	All causes	2019	Number	Infections of bones, joints, and related organs	KP	No infection	70	165	19
Deaths	Latin America and Caribbean	Both	All Ages	All causes	2019	Number	LRIs and all related infections in the thorax	KP	Drug-susceptible infection	4316	5808	3071
Deaths	Latin America and Caribbean	Both	All Ages	All causes	2019	Number	LRIs and all related infections in the thorax	KP	No infection	15351	19522	11992
Deaths	Latin America and Caribbean	Both	All Ages	All causes	2019	Number	Meningitis and other bacterial CNS infections	KP	Drug-susceptible infection	186	298	116
Deaths	Latin America and Caribbean	Both	All Ages	All causes	2019	Number	Meningitis and other bacterial CNS infections	KP	No infection	673	1064	451
Deaths	Latin America and Caribbean	Both	All Ages	All causes	2019	Number	Peritoneal and intra-abdominal infections	KP	Drug-susceptible infection	3205	4828	2002
Deaths	Latin America and Caribbean	Both	All Ages	All causes	2019	Number	Peritoneal and intra-abdominal infections	KP	No infection	11477	16541	7674
Deaths	Latin America and Caribbean	Both	All Ages	All causes	2019	Number	Urinary tract infections and pyelonephritis	KP	Drug-susceptible infection	992	1395	679
Deaths	Latin America and Caribbean	Both	All Ages	All causes	2019	Number	Urinary tract infections and pyelonephritis	KP	No infection	3481	4638	2612
Deaths	North Africa and Middle East	Both	All Ages	All causes	2019	Number	Bacterial infections ofthe skin and subcutaneous systems	KP	Drug-susceptible infection	90	340	11
Deaths	North Africa and Middle East	Both	All Ages	All causes	2019	Number	Bacterial infections ofthe skin and subcutaneous systems	KP	No infection	295	1132	35
Deaths	North Africa and Middle East	Both	All Ages	All causes	2019	Number	Bloodstream infections	KP	Drug-susceptible infection	4016	6768	2150
Deaths	North Africa and Middle East	Both	All Ages	All causes	2019	Number	Bloodstream infections	KP	No infection	13387	21301	7627
Deaths	North Africa and Middle East	Both	All Ages	All causes	2019	Number	Endocarditis and other cardiac infections	KP	Drug-susceptible infection	151	228	98
Deaths	North Africa and Middle East	Both	All Ages	All causes	2019	Number	Endocarditis and other cardiac infections	KP	No infection	495	708	343
Deaths	North Africa and Middle East	Both	All Ages	All causes	2019	Number	Infections of bones, joints, and related organs	KP	Drug-susceptible infection	30	76	7
Deaths	North Africa and Middle East	Both	All Ages	All causes	2019	Number	Infections of bones, joints, and related organs	KP	No infection	101	244	25
Deaths	North Africa and Middle East	Both	All Ages	All causes	2019	Number	LRIs and all related infections in the thorax	KP	Drug-susceptible infection	3525	4927	2364
Deaths	North Africa and Middle East	Both	All Ages	All causes	2019	Number	LRIs and all related infections in the thorax	KP	No infection	11900	15420	8994
Deaths	North Africa and Middle East	Both	All Ages	All causes	2019	Number	Meningitis and other bacterial CNS infections	KP	Drug-susceptible infection	361	675	194
Deaths	North Africa and Middle East	Both	All Ages	All causes	2019	Number	Meningitis and other bacterial CNS infections	KP	No infection	1228	2242	710
Deaths	North Africa and Middle East	Both	All Ages	All causes	2019	Number	Peritoneal and intra-abdominal infections	KP	Drug-susceptible infection	2765	4316	1631
Deaths	North Africa and Middle East	Both	All Ages	All causes	2019	Number	Peritoneal and intra-abdominal infections	KP	No infection	8533	12979	5218
Deaths	North Africa and Middle East	Both	All Ages	All causes	2019	Number	Urinary tract infections and pyelonephritis	KP	Drug-susceptible infection	379	686	184
Deaths	North Africa and Middle East	Both	All Ages	All causes	2019	Number	Urinary tract infections and pyelonephritis	KP	No infection	1223	2107	611
Deaths	South Asia	Both	All Ages	All causes	2019	Number	Bacterial infections ofthe skin and subcutaneous systems	KP	Drug-susceptible infection	506	2026	53
Deaths	South Asia	Both	All Ages	All causes	2019	Number	Bacterial infections ofthe skin and subcutaneous systems	KP	No infection	1413	5552	148
Deaths	South Asia	Both	All Ages	All causes	2019	Number	Bloodstream infections	KP	Drug-susceptible infection	20796	33393	11683
Deaths	South Asia	Both	All Ages	All causes	2019	Number	Bloodstream infections	KP	No infection	59573	95764	34167
Deaths	South Asia	Both	All Ages	All causes	2019	Number	Endocarditis and other cardiac infections	KP	Drug-susceptible infection	544	775	372
Deaths	South Asia	Both	All Ages	All causes	2019	Number	Endocarditis and other cardiac infections	KP	No infection	1539	2164	1088
Deaths	South Asia	Both	All Ages	All causes	2019	Number	Infections of bones, joints, and related organs	KP	Drug-susceptible infection	148	354	40
Deaths	South Asia	Both	All Ages	All causes	2019	Number	Infections of bones, joints, and related organs	KP	No infection	409	968	108
Deaths	South Asia	Both	All Ages	All causes	2019	Number	LRIs and all related infections in the thorax	KP	Drug-susceptible infection	22914	30145	17042
Deaths	South Asia	Both	All Ages	All causes	2019	Number	LRIs and all related infections in the thorax	KP	No infection	64980	84021	49662
Deaths	South Asia	Both	All Ages	All causes	2019	Number	Meningitis and other bacterial CNS infections	KP	Drug-susceptible infection	2520	3666	1746
Deaths	South Asia	Both	All Ages	All causes	2019	Number	Meningitis and other bacterial CNS infections	KP	No infection	7165	10283	5130
Deaths	South Asia	Both	All Ages	All causes	2019	Number	Peritoneal and intra-abdominal infections	KP	Drug-susceptible infection	11797	18456	7262
Deaths	South Asia	Both	All Ages	All causes	2019	Number	Peritoneal and intra-abdominal infections	KP	No infection	33011	50014	20782
Deaths	South Asia	Both	All Ages	All causes	2019	Number	Urinary tract infections and pyelonephritis	KP	Drug-susceptible infection	2568	3604	1777
Deaths	South Asia	Both	All Ages	All causes	2019	Number	Urinary tract infections and pyelonephritis	KP	No infection	7129	9867	5106
Deaths	Sub-Saharan Africa	Both	All Ages	All causes	2019	Number	Bacterial infections ofthe skin and subcutaneous systems	KP	Drug-susceptible infection	202	835	19
Deaths	Sub-Saharan Africa	Both	All Ages	All causes	2019	Number	Bacterial infections ofthe skin and subcutaneous systems	KP	No infection	745	3010	70
Deaths	Sub-Saharan Africa	Both	All Ages	All causes	2019	Number	Bloodstream infections	KP	Drug-susceptible infection	15192	23377	9088
Deaths	Sub-Saharan Africa	Both	All Ages	All causes	2019	Number	Bloodstream infections	KP	No infection	55863	80164	37077

Deaths	Sub-Saharan Africa	Both	All Ages	All causes	2019	Number	Endocarditis and other cardiac infections	KP	Drug-susceptible infection	215	318	136
Deaths	Sub-Saharan Africa	Both	All Ages	All causes	2019	Number	Endocarditis and other cardiac infections	KP	No infection	800	1079	595
Deaths	Sub-Saharan Africa	Both	All Ages	All causes	2019	Number	Infections of bones, joints, and related organs	KP	Drug-susceptible infection	57	148	15
Deaths	Sub-Saharan Africa	Both	All Ages	All causes	2019	Number	Infections of bones, joints, and related organs	KP	No infection	211	488	57
Deaths	Sub-Saharan Africa	Both	All Ages	All causes	2019	Number	LRIs and all related infections in the thorax	KP	Drug-susceptible infection	24504	34021	16556
Deaths	Sub-Saharan Africa	Both	All Ages	All causes	2019	Number	LRIs and all related infections in the thorax	KP	No infection	90135	112430	71663
Deaths	Sub-Saharan Africa	Both	All Ages	All causes	2019	Number	Meningitis and other bacterial CNS infections	KP	Drug-susceptible infection	5074	8100	2991
Deaths	Sub-Saharan Africa	Both	All Ages	All causes	2019	Number	Meningitis and other bacterial CNS infections	KP	No infection	18381	26567	12533
Deaths	Sub-Saharan Africa	Both	All Ages	All causes	2019	Number	Peritoneal and intra-abdominal infections	KP	Drug-susceptible infection	5227	8624	2864
Deaths	Sub-Saharan Africa	Both	All Ages	All causes	2019	Number	Peritoneal and intra-abdominal infections	KP	No infection	19135	29341	12214
Deaths	Sub-Saharan Africa	Both	All Ages	All causes	2019	Number	Urinary tract infections and pyelonephritis	KP	Drug-susceptible infection	318	504	182
Deaths	Sub-Saharan Africa	Both	All Ages	All causes	2019	Number	Urinary tract infections and pyelonephritis	KP	No infection	1171	1687	703

Figure 3

measure	location	sex	age	cause	year	metric	infectious syndrome	pathogen	counterfactual	value (per 100,000)	upper (per 100,000)	lower (per 100,000)
Deaths	Global	Both	All Ages	All causes	2019	Rate	Bacterial infections ofthe skin and subcutaneous systems	KP	Drug-susceptible infection	0.021	0.077	0.003
Deaths	Global	Both	All Ages	All causes	2019	Rate	Bacterial infections ofthe skin and subcutaneous systems	KP	No infection	0.069	0.256	0.010
Deaths	Global	Both	All Ages	All causes	2019	Rate	Bloodstream infections	KP	Drug-susceptible infection	0.831	1.350	0.467
Deaths	Global	Both	All Ages	All causes	2019	Rate	Bloodstream infections	KP	No infection	2.770	4.330	1.660
Deaths	Global	Both	All Ages	All causes	2019	Rate	Endocarditis and other cardiac infections	KP	Drug-susceptible infection	0.029	0.043	0.020
Deaths	Global	Both	All Ages	All causes	2019	Rate	Endocarditis and other cardiac infections	KP	No infection	0.101	0.140	0.074
Deaths	Global	Both	All Ages	All causes	2019	Rate	Infections of bones, joints, and related organs	KP	Drug-susceptible infection	0.005	0.011	0.001
Deaths	Global	Both	All Ages	All causes	2019	Rate	Infections of bones, joints, and related organs	KP	No infection	0.015	0.034	0.004
Deaths	Global	Both	All Ages	All causes	2019	Rate	LRIs and all related infections in the thorax	KP	Drug-susceptible infection	0.896	1.210	0.653
Deaths	Global	Both	All Ages	All causes	2019	Rate	LRIs and all related infections in the thorax	KP	No infection	3.000	3.730	2.390
Deaths	Global	Both	All Ages	All causes	2019	Rate	Meningitis and other bacterial CNS infections	KP	Drug-susceptible infection	0.116	0.176	0.076
Deaths	Global	Both	All Ages	All causes	2019	Rate	Meningitis and other bacterial CNS infections	KP	No infection	0.392	0.553	0.276
Deaths	Global	Both	All Ages	All causes	2019	Rate	Peritoneal and intra-abdominal infections	KP	Drug-susceptible infection	0.484	0.740	0.305
Deaths	Global	Both	All Ages	All causes	2019	Rate	Peritoneal and intra-abdominal infections	KP	No infection	1.590	2.360	1.040
Deaths	Global	Both	All Ages	All causes	2019	Rate	Urinary tract infections and pyelonephritis	KP	Drug-susceptible infection	0.108	0.161	0.071
Deaths	Global	Both	All Ages	All causes	2019	Rate	Urinary tract infections and pyelonephritis	KP	No infection	0.358	0.521	0.246
Deaths	Southeast Asia, East Asia, and Oceania	Both	All Ages	All causes	2019	Rate	Bacterial infections ofthe skin and subcutaneous systems	KP	Drug-susceptible infection	0.018	0.064	0.002
Deaths	Southeast Asia, East Asia, and Oceania	Both	All Ages	All causes	2019	Rate	Bacterial infections ofthe skin and subcutaneous systems	KP	No infection	0.063	0.222	0.009
Deaths	Southeast Asia, East Asia, and Oceania	Both	All Ages	All causes	2019	Rate	Bloodstream infections	KP	Drug-susceptible infection	0.508	0.876	0.258
Deaths	Southeast Asia, East Asia, and Oceania	Both	All Ages	All causes	2019	Rate	Bloodstream infections	KP	No infection	1.760	2.890	0.992
Deaths	Southeast Asia, East Asia, and Oceania	Both	All Ages	All causes	2019	Rate	Endocarditis and other cardiac infections	KP	Drug-susceptible infection	0.017	0.028	0.011
Deaths	Southeast Asia, East Asia, and Oceania	Both	All Ages	All causes	2019	Rate	Endocarditis and other cardiac infections	KP	No infection	0.060	0.090	0.040
Deaths	Southeast Asia, East Asia, and Oceania	Both	All Ages	All causes	2019	Rate	Infections of bones, joints, and related organs	KP	Drug-susceptible infection	0.003	0.007	0.001
Deaths	Southeast Asia, East Asia, and Oceania	Both	All Ages	All causes	2019	Rate	Infections of bones, joints, and related organs	KP	No infection	0.009	0.022	0.003
Deaths	Southeast Asia, East Asia, and Oceania	Both	All Ages	All causes	2019	Rate	LRIs and all related infections in the thorax	KP	Drug-susceptible infection	0.381	0.557	0.249
Deaths	Southeast Asia, East Asia, and Oceania	Both	All Ages	All causes	2019	Rate	LRIs and all related infections in the thorax	KP	No infection	1.300	1.710	0.967
Deaths	Southeast Asia, East Asia, and Oceania	Both	All Ages	All causes	2019	Rate	Meningitis and other bacterial CNS infections	KP	Drug-susceptible infection	0.029	0.050	0.017
Deaths	Southeast Asia, East Asia, and Oceania	Both	All Ages	All causes	2019	Rate	Meningitis and other bacterial CNS infections	KP	No infection	0.098	0.162	0.065
Deaths	Southeast Asia, East Asia, and Oceania	Both	All Ages	All causes	2019	Rate	Peritoneal and intra-abdominal infections	KP	Drug-susceptible infection	0.356	0.587	0.203
Deaths	Southeast Asia, East Asia, and Oceania	Both	All Ages	All causes	2019	Rate	Peritoneal and intra-abdominal infections	KP	No infection	1.230	1.880	0.770
Deaths	Southeast Asia, East Asia, and Oceania	Both	All Ages	All causes	2019	Rate	Urinary tract infections and pyelonephritis	KP	Drug-susceptible infection	0.081	0.148	0.041
Deaths	Southeast Asia, East Asia, and Oceania	Both	All Ages	All causes	2019	Rate	Urinary tract infections and pyelonephritis	KP	No infection	0.283	0.489	0.150
Deaths	Central Europe, Eastern Europe, and Central Asia	Both	All Ages	All causes	2019	Rate	Bacterial infections ofthe skin and subcutaneous systems	KP	Drug-susceptible infection	0.032	0.114	0.005
Deaths	Central Europe, Eastern Europe, and Central Asia	Both	All Ages	All causes	2019	Rate	Bacterial infections ofthe skin and subcutaneous systems	KP	No infection	0.102	0.357	0.015
Deaths	Central Europe, Eastern Europe, and Central Asia	Both	All Ages	All causes	2019	Rate	Bloodstream infections	KP	Drug-susceptible infection	1.170	2.030	0.608
Deaths	Central Europe, Eastern Europe, and Central Asia	Both	All Ages	All causes	2019	Rate	Bloodstream infections	KP	No infection	3.780	6.400	2.030
Deaths	Central Europe, Eastern Europe, and Central Asia	Both	All Ages	All causes	2019	Rate	Endocarditis and other cardiac infections	KP	Drug-susceptible infection	0.079	0.134	0.044
Deaths	Central Europe, Eastern Europe, and Central Asia	Both	All Ages	All causes	2019	Rate	Endocarditis and other cardiac infections	KP	No infection	0.260	0.430	0.150
Deaths	Central Europe, Eastern Europe, and Central Asia	Both	All Ages	All causes	2019	Rate	Infections of bones, joints, and related organs	KP	Drug-susceptible infection	0.004	0.010	0.001
Deaths	Central Europe, Eastern Europe, and Central Asia	Both	All Ages	All causes	2019	Rate	Infections of bones, joints, and related organs	KP	No infection	0.014	0.033	0.004
Deaths	Central Europe, Eastern Europe, and Central Asia	Both	All Ages	All causes	2019	Rate	LRIs and all related infections in the thorax	KP	Drug-susceptible infection	0.618	0.872	0.432
Deaths	Central Europe, Eastern Europe, and Central Asia	Both	All Ages	All causes	2019	Rate	LRIs and all related infections in the thorax	KP	No infection	2.010	2.700	1.530
Deaths	Central Europe, Eastern Europe, and Central Asia	Both	All Ages	All causes	2019	Rate	Meningitis and other bacterial CNS infections	KP	Drug-susceptible infection	0.032	0.057	0.020
Deaths	Central Europe, Eastern Europe, and Central Asia	Both	All Ages	All causes	2019	Rate	Meningitis and other bacterial CNS infections	KP	No infection	0.104	0.185	0.067
Deaths	Central Europe, Eastern Europe, and Central Asia	Both	All Ages	All causes	2019	Rate	Peritoneal and intra-abdominal infections	KP	Drug-susceptible infection	0.846	1.290	0.534
Deaths	Central Europe, Eastern Europe, and Central Asia	Both	All Ages	All causes	2019	Rate	Peritoneal and intra-abdominal infections	KP	No infection	2.740	4.000	1.770
Deaths	Central Europe, Eastern Europe, and Central Asia	Both	All Ages	All causes	2019	Rate	Urinary tract infections and pyelonephritis	KP	Drug-susceptible infection	0.245	0.382	0.153
Deaths	Central Europe, Eastern Europe, and Central Asia	Both	All Ages	All causes	2019	Rate	Urinary tract infections and pyelonephritis	KP	No infection	0.786	1.190	0.512
Deaths	High-income	Both	All Ages	All causes	2019	Rate	Bacterial infections ofthe skin and subcutaneous systems	KP	Drug-susceptible infection	0.014	0.047	0.002
Deaths	High-income	Both	All Ages	All causes	2019	Rate	Bacterial infections ofthe skin and subcutaneous systems	KP	No infection	0.057	0.188	0.009

Deaths	High-income	Both	All Ages	All causes	2019	Rate	Bloodstream infections	KP	Drug-susceptible infection	0.365	0.633	0.188
Deaths	High-income	Both	All Ages	All causes	2019	Rate	Bloodstream infections	KP	No infection	1.460	2.470	0.794
Deaths	High-income	Both	All Ages	All causes	2019	Rate	Endocarditis and other cardiac infections	KP	Drug-susceptible infection	0.044	0.065	0.026
Deaths	High-income	Both	All Ages	All causes	2019	Rate	Endocarditis and other cardiac infections	KP	No infection	0.180	0.244	0.116
Deaths	High-income	Both	All Ages	All causes	2019	Rate	Infections of bones, joints, and related organs	KP	Drug-susceptible infection	0.002	0.004	0.000
Deaths	High-income	Both	All Ages	All causes	2019	Rate	Infections of bones, joints, and related organs	KP	No infection	0.006	0.014	0.002
Deaths	High-income	Both	All Ages	All causes	2019	Rate	LRIs and all related infections in the thorax	KP	Drug-susceptible infection	0.296	0.407	0.213
Deaths	High-income	Both	All Ages	All causes	2019	Rate	LRIs and all related infections in the thorax	KP	No infection	1.190	1.460	0.971
Deaths	High-income	Both	All Ages	All causes	2019	Rate	Meningitis and other bacterial CNS infections	KP	Drug-susceptible infection	0.007	0.013	0.004
Deaths	High-income	Both	All Ages	All causes	2019	Rate	Meningitis and other bacterial CNS infections	KP	No infection	0.028	0.049	0.018
Deaths	High-income	Both	All Ages	All causes	2019	Rate	Peritoneal and intra-abdominal infections	KP	Drug-susceptible infection	0.298	0.453	0.178
Deaths	High-income	Both	All Ages	All causes	2019	Rate	Peritoneal and intra-abdominal infections	KP	No infection	1.200	1.760	0.797
Deaths	High-income	Both	All Ages	All causes	2019	Rate	Urinary tract infections and pyelonephritis	KP	Drug-susceptible infection	0.120	0.169	0.082
Deaths	High-income	Both	All Ages	All causes	2019	Rate	Urinary tract infections and pyelonephritis	KP	No infection	0.487	0.649	0.368
Deaths	Latin America and Caribbean	Both	All Ages	All causes	2019	Rate	Bacterial infections ofthe skin and subcutaneous systems	KP	Drug-susceptible infection	0.024	0.081	0.004
Deaths	Latin America and Caribbean	Both	All Ages	All causes	2019	Rate	Bacterial infections ofthe skin and subcutaneous systems	KP	No infection	0.085	0.281	0.014
Deaths	Latin America and Caribbean	Both	All Ages	All causes	2019	Rate	Bloodstream infections	KP	Drug-susceptible infection	0.767	1.280	0.413
Deaths	Latin America and Caribbean	Both	All Ages	All causes	2019	Rate	Bloodstream infections	KP	No infection	2.740	4.370	1.580
Deaths	Latin America and Caribbean	Both	All Ages	All causes	2019	Rate	Endocarditis and other cardiac infections	KP	Drug-susceptible infection	0.030	0.041	0.021
Deaths	Latin America and Caribbean	Both	All Ages	All causes	2019	Rate	Endocarditis and other cardiac infections	KP	No infection	0.105	0.134	0.080
Deaths	Latin America and Caribbean	Both	All Ages	All causes	2019	Rate	Infections of bones, joints, and related organs	KP	Drug-susceptible infection	0.003	0.008	0.001
Deaths	Latin America and Caribbean	Both	All Ages	All causes	2019	Rate	Infections of bones, joints, and related organs	KP	No infection	0.012	0.028	0.003
Deaths	Latin America and Caribbean	Both	All Ages	All causes	2019	Rate	LRIs and all related infections in the thorax	KP	Drug-susceptible infection	0.738	0.994	0.525
Deaths	Latin America and Caribbean	Both	All Ages	All causes	2019	Rate	LRIs and all related infections in the thorax	KP	No infection	2.630	3.340	2.050
Deaths	Latin America and Caribbean	Both	All Ages	All causes	2019	Rate	Meningitis and other bacterial CNS infections	KP	Drug-susceptible infection	0.032	0.051	0.020
Deaths	Latin America and Caribbean	Both	All Ages	All causes	2019	Rate	Meningitis and other bacterial CNS infections	KP	No infection	0.115	0.182	0.077
Deaths	Latin America and Caribbean	Both	All Ages	All causes	2019	Rate	Peritoneal and intra-abdominal infections	KP	Drug-susceptible infection	0.549	0.826	0.343
Deaths	Latin America and Caribbean	Both	All Ages	All causes	2019	Rate	Peritoneal and intra-abdominal infections	KP	No infection	1.960	2.830	1.310
Deaths	Latin America and Caribbean	Both	All Ages	All causes	2019	Rate	Urinary tract infections and pyelonephritis	KP	Drug-susceptible infection	0.170	0.239	0.116
Deaths	Latin America and Caribbean	Both	All Ages	All causes	2019	Rate	Urinary tract infections and pyelonephritis	KP	No infection	0.596	0.794	0.447
Deaths	North Africa and Middle East	Both	All Ages	All causes	2019	Rate	Bacterial infections ofthe skin and subcutaneous systems	KP	Drug-susceptible infection	0.015	0.056	0.002
Deaths	North Africa and Middle East	Both	All Ages	All causes	2019	Rate	Bacterial infections ofthe skin and subcutaneous systems	KP	No infection	0.048	0.186	0.006
Deaths	North Africa and Middle East	Both	All Ages	All causes	2019	Rate	Bloodstream infections	KP	Drug-susceptible infection	0.660	1.110	0.353
Deaths	North Africa and Middle East	Both	All Ages	All causes	2019	Rate	Bloodstream infections	KP	No infection	2.200	3.500	1.250
Deaths	North Africa and Middle East	Both	All Ages	All causes	2019	Rate	Endocarditis and other cardiac infections	KP	Drug-susceptible infection	0.025	0.037	0.016
Deaths	North Africa and Middle East	Both	All Ages	All causes	2019	Rate	Endocarditis and other cardiac infections	KP	No infection	0.081	0.116	0.056
Deaths	North Africa and Middle East	Both	All Ages	All causes	2019	Rate	Infections of bones, joints, and related organs	KP	Drug-susceptible infection	0.005	0.013	0.001
Deaths	North Africa and Middle East	Both	All Ages	All causes	2019	Rate	Infections of bones, joints, and related organs	KP	No infection	0.017	0.040	0.004
Deaths	North Africa and Middle East	Both	All Ages	All causes	2019	Rate	LRIs and all related infections in the thorax	KP	Drug-susceptible infection	0.579	0.809	0.388
Deaths	North Africa and Middle East	Both	All Ages	All causes	2019	Rate	LRIs and all related infections in the thorax	KP	No infection	1.950	2.530	1.480
Deaths	North Africa and Middle East	Both	All Ages	All causes	2019	Rate	Meningitis and other bacterial CNS infections	KP	Drug-susceptible infection	0.059	0.111	0.032
Deaths	North Africa and Middle East	Both	All Ages	All causes	2019	Rate	Meningitis and other bacterial CNS infections	KP	No infection	0.202	0.368	0.117
Deaths	North Africa and Middle East	Both	All Ages	All causes	2019	Rate	Peritoneal and intra-abdominal infections	KP	Drug-susceptible infection	0.454	0.709	0.268
Deaths	North Africa and Middle East	Both	All Ages	All causes	2019	Rate	Peritoneal and intra-abdominal infections	KP	No infection	1.400	2.130	0.857
Deaths	North Africa and Middle East	Both	All Ages	All causes	2019	Rate	Urinary tract infections and pyelonephritis	KP	Drug-susceptible infection	0.062	0.113	0.030
Deaths	North Africa and Middle East	Both	All Ages	All causes	2019	Rate	Urinary tract infections and pyelonephritis	KP	No infection	0.201	0.346	0.100
Deaths	South Asia	Both	All Ages	All causes	2019	Rate	Bacterial infections ofthe skin and subcutaneous systems	KP	Drug-susceptible infection	0.028	0.112	0.003
Deaths	South Asia	Both	All Ages	All causes	2019	Rate	Bacterial infections ofthe skin and subcutaneous systems	KP	No infection	0.078	0.308	0.008
Deaths	South Asia	Both	All Ages	All causes	2019	Rate	Bloodstream infections	KP	Drug-susceptible infection	1.150	1.850	0.647
Deaths	South Asia	Both	All Ages	All causes	2019	Rate	Bloodstream infections	KP	No infection	3.300	5.300	1.890
Deaths	South Asia	Both	All Ages	All causes	2019	Rate	Endocarditis and other cardiac infections	KP	Drug-susceptible infection	0.030	0.043	0.021
Deaths	South Asia	Both	All Ages	All causes	2019	Rate	Endocarditis and other cardiac infections	KP	No infection	0.085	0.120	0.060
Deaths	South Asia	Both	All Ages	All causes	2019	Rate	Infections of bones, joints, and related organs	KP	Drug-susceptible infection	0.008	0.020	0.002
Deaths	South Asia	Both	All Ages	All causes	2019	Rate	Infections of bones, joints, and related organs	KP	No infection	0.023	0.054	0.006

Deaths	South Asia	Both	All Ages	All causes	2019	Rate	LRIs and all related infections in the thorax	KP	Drug-susceptible infection	1.270	1.670	0.944
Deaths	South Asia	Both	All Ages	All causes	2019	Rate	LRIs and all related infections in the thorax	KP	No infection	3.600	4.650	2.750
Deaths	South Asia	Both	All Ages	All causes	2019	Rate	Meningitis and other bacterial CNS infections	KP	Drug-susceptible infection	0.140	0.203	0.097
Deaths	South Asia	Both	All Ages	All causes	2019	Rate	Meningitis and other bacterial CNS infections	KP	No infection	0.397	0.570	0.284
Deaths	South Asia	Both	All Ages	All causes	2019	Rate	Peritoneal and intra-abdominal infections	KP	Drug-susceptible infection	0.654	1.020	0.402
Deaths	South Asia	Both	All Ages	All causes	2019	Rate	Peritoneal and intra-abdominal infections	KP	No infection	1.830	2.770	1.150
Deaths	South Asia	Both	All Ages	All causes	2019	Rate	Urinary tract infections and pyelonephritis	KP	Drug-susceptible infection	0.142	0.200	0.098
Deaths	South Asia	Both	All Ages	All causes	2019	Rate	Urinary tract infections and pyelonephritis	KP	No infection	0.395	0.547	0.283
Deaths	Sub-Saharan Africa	Both	All Ages	All causes	2019	Rate	Bacterial infections ofthe skin and subcutaneous systems	KP	Drug-susceptible infection	0.019	0.078	0.002
Deaths	Sub-Saharan Africa	Both	All Ages	All causes	2019	Rate	Bacterial infections ofthe skin and subcutaneous systems	KP	No infection	0.069	0.279	0.006
Deaths	Sub-Saharan Africa	Both	All Ages	All causes	2019	Rate	Bloodstream infections	KP	Drug-susceptible infection	1.410	2.170	0.843
Deaths	Sub-Saharan Africa	Both	All Ages	All causes	2019	Rate	Bloodstream infections	KP	No infection	5.180	7.430	3.440
Deaths	Sub-Saharan Africa	Both	All Ages	All causes	2019	Rate	Endocarditis and other cardiac infections	KP	Drug-susceptible infection	0.020	0.030	0.013
Deaths	Sub-Saharan Africa	Both	All Ages	All causes	2019	Rate	Endocarditis and other cardiac infections	KP	No infection	0.074	0.100	0.055
Deaths	Sub-Saharan Africa	Both	All Ages	All causes	2019	Rate	Infections of bones, joints, and related organs	KP	Drug-susceptible infection	0.005	0.014	0.001
Deaths	Sub-Saharan Africa	Both	All Ages	All causes	2019	Rate	Infections of bones, joints, and related organs	KP	No infection	0.020	0.045	0.005
Deaths	Sub-Saharan Africa	Both	All Ages	All causes	2019	Rate	LRIs and all related infections in the thorax	KP	Drug-susceptible infection	2.270	3.160	1.540
Deaths	Sub-Saharan Africa	Both	All Ages	All causes	2019	Rate	LRIs and all related infections in the thorax	KP	No infection	8.360	10.427	6.650
Deaths	Sub-Saharan Africa	Both	All Ages	All causes	2019	Rate	Meningitis and other bacterial CNS infections	KP	Drug-susceptible infection	0.471	0.751	0.277
Deaths	Sub-Saharan Africa	Both	All Ages	All causes	2019	Rate	Meningitis and other bacterial CNS infections	KP	No infection	1.700	2.460	1.160
Deaths	Sub-Saharan Africa	Both	All Ages	All causes	2019	Rate	Peritoneal and intra-abdominal infections	KP	Drug-susceptible infection	0.485	0.800	0.266
Deaths	Sub-Saharan Africa	Both	All Ages	All causes	2019	Rate	Peritoneal and intra-abdominal infections	KP	No infection	1.770	2.720	1.130
Deaths	Sub-Saharan Africa	Both	All Ages	All causes	2019	Rate	Urinary tract infections and pyelonephritis	KP	Drug-susceptible infection	0.030	0.047	0.017
Deaths	Sub-Saharan Africa	Both	All Ages	All causes	2019	Rate	Urinary tract infections and pyelonephritis	KP	No infection	0.109	0.156	0.065

Figure 4A

measure	location	sex	age	cause	year	metric	infectious syndrome	pathogen	associated with resistance			attributable to resistance		
									value	upper	lower	value	upper	lower
Deaths	South Asia	Both	All Ages	All causes	2019	Number	All infectious syndromes	KP	175218	241051	124250	61793	87875	42813
Deaths	Western Sub-Saharan Africa	Both	All Ages	All causes	2019	Number	All infectious syndromes	KP	93498	120066	71731	25792	36862	17289
Deaths	Eastern Sub-Saharan Africa	Both	All Ages	All causes	2019	Number	All infectious syndromes	KP	63495	81661	48820	17182	24557	11250
Deaths	East Asia	Both	All Ages	All causes	2019	Number	All infectious syndromes	KP	52680	81774	31657	14482	25390	7323
Deaths	Southeast Asia	Both	All Ages	All causes	2019	Number	All infectious syndromes	KP	49646	68918	34664	15241	22239	9814
Deaths	North Africa and Middle East	Both	All Ages	All causes	2019	Number	All infectious syndromes	KP	37162	53370	25053	11317	16947	7133
Deaths	Western Europe	Both	All Ages	All causes	2019	Number	All infectious syndromes	KP	23899	33107	16885	5917	8798	3855
Deaths	Eastern Europe	Both	All Ages	All causes	2019	Number	All infectious syndromes	KP	22459	32449	14632	7460	11076	4788
Deaths	Tropical Latin America	Both	All Ages	All causes	2019	Number	All infectious syndromes	KP	19817	26427	14541	5902	8241	4110
Deaths	Central Sub-Saharan Africa	Both	All Ages	All causes	2019	Number	All infectious syndromes	KP	18649	24503	14121	4850	7137	3029
Deaths	Central Latin America	Both	All Ages	All causes	2019	Number	All infectious syndromes	KP	17397	24593	11801	4676	7181	2827
Deaths	High-income North America	Both	All Ages	All causes	2019	Number	All infectious syndromes	KP	11644	16558	8083	2801	4332	1786
Deaths	Central Europe	Both	All Ages	All causes	2019	Number	All infectious syndromes	KP	10955	16441	7044	3145	4895	1889
Deaths	Southern Sub-Saharan Africa	Both	All Ages	All causes	2019	Number	All infectious syndromes	KP	10793	13960	8254	2966	4351	1888
Deaths	Central Asia	Both	All Ages	All causes	2019	Number	All infectious syndromes	KP	7525	10733	5130	2039	3193	1200
Deaths	High-income Asia Pacific	Both	All Ages	All causes	2019	Number	All infectious syndromes	KP	7043	9524	5182	1610	2430	998
Deaths	Southern Latin America	Both	All Ages	All causes	2019	Number	All infectious syndromes	KP	6762	8897	5095	1944	2800	1315
Deaths	Andean Latin America	Both	All Ages	All causes	2019	Number	All infectious syndromes	KP	6479	8868	4531	1758	2585	1132
Deaths	Caribbean	Both	All Ages	All causes	2019	Number	All infectious syndromes	KP	4478	6331	3092	1174	1827	703
Deaths	Oceania	Both	All Ages	All causes	2019	Number	All infectious syndromes	KP	1425	1981	989	394	611	223
Deaths	Australasia	Both	All Ages	All causes	2019	Number	All infectious syndromes	KP	712	1030	475	149	233	92

Figure 4B

measure	location	sex	age	cause	year	metric	infectious syndrome	pathogen	associated with resistance (per 100,000)			attributable to resistance (per 100,000)		
									value	upper	lower	value	upper	lower
Deaths	Western Sub-Saharan Africa	Both	All Ages	All causes	2019	Rate	All infectious syndromes	KP	20.49	26.31	15.72	5.65	8.08	3.79
Deaths	Eastern Sub-Saharan Africa	Both	All Ages	All causes	2019	Rate	All infectious syndromes	KP	15.42	19.83	11.86	4.17	5.96	2.73
Deaths	Central Sub-Saharan Africa	Both	All Ages	All causes	2019	Rate	All infectious syndromes	KP	14.18	18.63	10.73	3.69	5.43	2.30
Deaths	Southern Sub-Saharan Africa	Both	All Ages	All causes	2019	Rate	All infectious syndromes	KP	13.74	17.77	10.50	3.77	5.54	2.40
Deaths	Oceania	Both	All Ages	All causes	2019	Rate	All infectious syndromes	KP	10.74	14.92	7.45	2.97	4.60	1.68
Deaths	Eastern Europe	Both	All Ages	All causes	2019	Rate	All infectious syndromes	KP	10.70	15.45	6.97	3.55	5.27	2.28
Deaths	Andean Latin America	Both	All Ages	All causes	2019	Rate	All infectious syndromes	KP	10.19	13.95	7.12	2.76	4.07	1.78
Deaths	Southern Latin America	Both	All Ages	All causes	2019	Rate	All infectious syndromes	KP	10.13	13.33	7.63	2.91	4.19	1.97
Deaths	South Asia	Both	All Ages	All causes	2019	Rate	All infectious syndromes	KP	9.71	13.35	6.88	3.42	4.87	2.37
Deaths	Central Europe	Both	All Ages	All causes	2019	Rate	All infectious syndromes	KP	9.59	14.39	6.17	2.75	4.29	1.65
Deaths	Caribbean	Both	All Ages	All causes	2019	Rate	All infectious syndromes	KP	9.49	13.42	6.56	2.49	3.87	1.49
Deaths	Tropical Latin America	Both	All Ages	All causes	2019	Rate	All infectious syndromes	KP	8.86	11.82	6.50	2.64	3.69	1.84
Deaths	Central Asia	Both	All Ages	All causes	2019	Rate	All infectious syndromes	KP	8.04	11.48	5.48	2.18	3.41	1.28
Deaths	Southeast Asia	Both	All Ages	All causes	2019	Rate	All infectious syndromes	KP	7.37	10.23	5.14	2.26	3.30	1.46
Deaths	Central Latin America	Both	All Ages	All causes	2019	Rate	All infectious syndromes	KP	6.96	9.84	4.72	1.87	2.87	1.13
Deaths	North Africa and Middle East	Both	All Ages	All causes	2019	Rate	All infectious syndromes	KP	6.10	8.77	4.12	1.86	2.78	1.17
Deaths	Western Europe	Both	All Ages	All causes	2019	Rate	All infectious syndromes	KP	5.48	7.59	3.87	1.36	2.02	0.88
Deaths	High-income Asia Pacific	Both	All Ages	All causes	2019	Rate	All infectious syndromes	KP	3.76	5.08	2.77	0.86	1.30	0.53
Deaths	East Asia	Both	All Ages	All causes	2019	Rate	All infectious syndromes	KP	3.58	5.55	2.15	0.98	1.72	0.50
Deaths	High-income North America	Both	All Ages	All causes	2019	Rate	All infectious syndromes	KP	3.19	4.54	2.22	0.77	1.19	0.49
Deaths	Australasia	Both	All Ages	All causes	2019	Rate	All infectious syndromes	KP	2.45	3.54	1.63	0.51	0.80	0.32

S.Figure 1

measure	location	sex	age	cause	year	metric	infectious syndrome	pathogen	counterfactual	value	upper	lower
Deaths	Global	Both	All Ages	All causes	2019	Number	All infectious syndromes	KP	Drug-susceptible infection	192591	272017	130181
Deaths	Global	Both	All Ages	All causes	2019	Number	All infectious syndromes	KP	No infection	641703	863302	465322
Deaths	East Asia	Both	All Ages	All causes	2019	Number	All infectious syndromes	KP	Drug-susceptible infection	14482	25390	7323
Deaths	East Asia	Both	All Ages	All causes	2019	Number	All infectious syndromes	KP	No infection	52680	81774	31657
Deaths	Southeast Asia	Both	All Ages	All causes	2019	Number	All infectious syndromes	KP	Drug-susceptible infection	15241	22239	9814
Deaths	Southeast Asia	Both	All Ages	All causes	2019	Number	All infectious syndromes	KP	No infection	49646	68918	34664
Deaths	Oceania	Both	All Ages	All causes	2019	Number	All infectious syndromes	KP	Drug-susceptible infection	394	611	223
Deaths	Oceania	Both	All Ages	All causes	2019	Number	All infectious syndromes	KP	No infection	1425	1981	989
Deaths	Central Asia	Both	All Ages	All causes	2019	Number	All infectious syndromes	KP	Drug-susceptible infection	2039	3193	1200
Deaths	Central Asia	Both	All Ages	All causes	2019	Number	All infectious syndromes	KP	No infection	7525	10733	5130
Deaths	Central Europe	Both	All Ages	All causes	2019	Number	All infectious syndromes	KP	Drug-susceptible infection	3145	4895	1889
Deaths	Central Europe	Both	All Ages	All causes	2019	Number	All infectious syndromes	KP	No infection	10955	16441	7044
Deaths	Eastern Europe	Both	All Ages	All causes	2019	Number	All infectious syndromes	KP	Drug-susceptible infection	7460	11076	4788
Deaths	Eastern Europe	Both	All Ages	All causes	2019	Number	All infectious syndromes	KP	No infection	22459	32449	14632
Deaths	High-income Asia Pacific	Both	All Ages	All causes	2019	Number	All infectious syndromes	KP	Drug-susceptible infection	1610	2430	998
Deaths	High-income Asia Pacific	Both	All Ages	All causes	2019	Number	All infectious syndromes	KP	No infection	7043	9524	5182
Deaths	Australasia	Both	All Ages	All causes	2019	Number	All infectious syndromes	KP	Drug-susceptible infection	149	233	92
Deaths	Australasia	Both	All Ages	All causes	2019	Number	All infectious syndromes	KP	No infection	712	1030	475
Deaths	Western Europe	Both	All Ages	All causes	2019	Number	All infectious syndromes	KP	Drug-susceptible infection	5917	8798	3855
Deaths	Western Europe	Both	All Ages	All causes	2019	Number	All infectious syndromes	KP	No infection	23899	33107	16885
Deaths	Southern Latin America	Both	All Ages	All causes	2019	Number	All infectious syndromes	KP	Drug-susceptible infection	1944	2800	1315
Deaths	Southern Latin America	Both	All Ages	All causes	2019	Number	All infectious syndromes	KP	No infection	6762	8897	5095
Deaths	High-income North America	Both	All Ages	All causes	2019	Number	All infectious syndromes	KP	Drug-susceptible infection	2801	4332	1786
Deaths	High-income North America	Both	All Ages	All causes	2019	Number	All infectious syndromes	KP	No infection	11644	16558	8083
Deaths	Caribbean	Both	All Ages	All causes	2019	Number	All infectious syndromes	KP	Drug-susceptible infection	1174	1827	703
Deaths	Caribbean	Both	All Ages	All causes	2019	Number	All infectious syndromes	KP	No infection	4478	6331	3092
Deaths	Andean Latin America	Both	All Ages	All causes	2019	Number	All infectious syndromes	KP	Drug-susceptible infection	1758	2585	1132
Deaths	Andean Latin America	Both	All Ages	All causes	2019	Number	All infectious syndromes	KP	No infection	6479	8868	4531
Deaths	Central Latin America	Both	All Ages	All causes	2019	Number	All infectious syndromes	KP	Drug-susceptible infection	4676	7181	2827
Deaths	Central Latin America	Both	All Ages	All causes	2019	Number	All infectious syndromes	KP	No infection	17397	24593	11801
Deaths	Tropical Latin America	Both	All Ages	All causes	2019	Number	All infectious syndromes	KP	Drug-susceptible infection	5902	8241	4110
Deaths	Tropical Latin America	Both	All Ages	All causes	2019	Number	All infectious syndromes	KP	No infection	19817	26427	14541
Deaths	North Africa and Middle East	Both	All Ages	All causes	2019	Number	All infectious syndromes	KP	Drug-susceptible infection	11317	16947	7133
Deaths	North Africa and Middle East	Both	All Ages	All causes	2019	Number	All infectious syndromes	KP	No infection	37162	53370	25053
Deaths	South Asia	Both	All Ages	All causes	2019	Number	All infectious syndromes	KP	Drug-susceptible infection	61793	87875	42813
Deaths	South Asia	Both	All Ages	All causes	2019	Number	All infectious syndromes	KP	No infection	175218	241051	124250
Deaths	Central Sub-Saharan Africa	Both	All Ages	All causes	2019	Number	All infectious syndromes	KP	Drug-susceptible infection	4850	7137	3029
Deaths	Central Sub-Saharan Africa	Both	All Ages	All causes	2019	Number	All infectious syndromes	KP	No infection	18649	24503	14121
Deaths	Eastern Sub-Saharan Africa	Both	All Ages	All causes	2019	Number	All infectious syndromes	KP	Drug-susceptible infection	17182	24557	11250
Deaths	Eastern Sub-Saharan Africa	Both	All Ages	All causes	2019	Number	All infectious syndromes	KP	No infection	63495	81661	48820
Deaths	Southern Sub-Saharan Africa	Both	All Ages	All causes	2019	Number	All infectious syndromes	KP	Drug-susceptible infection	2966	4351	1888
Deaths	Southern Sub-Saharan Africa	Both	All Ages	All causes	2019	Number	All infectious syndromes	KP	No infection	10793	13960	8254
Deaths	Western Sub-Saharan Africa	Both	All Ages	All causes	2019	Number	All infectious syndromes	KP	Drug-susceptible infection	25792	36862	17289
Deaths	Western Sub-Saharan Africa	Both	All Ages	All causes	2019	Number	All infectious syndromes	KP	No infection	93498	120066	71731

Deaths	Global	Both	All Ages	All causes	2019	Number	Bacterial infections ofthe skin and subcutaneous systems	KP	Drug-susceptible infection	1614	5952	229
Deaths	Global	Both	All Ages	All causes	2019	Number	Bacterial infections ofthe skin and subcutaneous systems	KP	No infection	5343	19802	773
Deaths	East Asia	Both	All Ages	All causes	2019	Number	Bacterial infections ofthe skin and subcutaneous systems	KP	Drug-susceptible infection	218	812	25
Deaths	East Asia	Both	All Ages	All causes	2019	Number	Bacterial infections ofthe skin and subcutaneous systems	KP	No infection	793	2925	98
Deaths	Southeast Asia	Both	All Ages	All causes	2019	Number	Bacterial infections ofthe skin and subcutaneous systems	KP	Drug-susceptible infection	170	610	25
Deaths	Southeast Asia	Both	All Ages	All causes	2019	Number	Bacterial infections ofthe skin and subcutaneous systems	KP	No infection	551	2033	80
Deaths	Oceania	Both	All Ages	All causes	2019	Number	Bacterial infections ofthe skin and subcutaneous systems	KP	Drug-susceptible infection	2	9	0
Deaths	Oceania	Both	All Ages	All causes	2019	Number	Bacterial infections ofthe skin and subcutaneous systems	KP	No infection	9	36	1
Deaths	Central Asia	Both	All Ages	All causes	2019	Number	Bacterial infections ofthe skin and subcutaneous systems	KP	Drug-susceptible infection	13	48	1
Deaths	Central Asia	Both	All Ages	All causes	2019	Number	Bacterial infections ofthe skin and subcutaneous systems	KP	No infection	47	182	5
Deaths	Central Europe	Both	All Ages	All causes	2019	Number	Bacterial infections ofthe skin and subcutaneous systems	KP	Drug-susceptible infection	31	118	4
Deaths	Central Europe	Both	All Ages	All causes	2019	Number	Bacterial infections ofthe skin and subcutaneous systems	KP	No infection	108	398	14
Deaths	Eastern Europe	Both	All Ages	All causes	2019	Number	Bacterial infections ofthe skin and subcutaneous systems	KP	Drug-susceptible infection	90	317	14
Deaths	Eastern Europe	Both	All Ages	All causes	2019	Number	Bacterial infections ofthe skin and subcutaneous systems	KP	No infection	269	938	41
Deaths	High-income Asia Pacific	Both	All Ages	All causes	2019	Number	Bacterial infections ofthe skin and subcutaneous systems	KP	Drug-susceptible infection	15	52	2
Deaths	High-income Asia Pacific	Both	All Ages	All causes	2019	Number	Bacterial infections ofthe skin and subcutaneous systems	KP	No infection	65	232	9
Deaths	Australasia	Both	All Ages	All causes	2019	Number	Bacterial infections ofthe skin and subcutaneous systems	KP	Drug-susceptible infection	3	9	0
Deaths	Australasia	Both	All Ages	All causes	2019	Number	Bacterial infections ofthe skin and subcutaneous systems	KP	No infection	13	42	2
Deaths	Western Europe	Both	All Ages	All causes	2019	Number	Bacterial infections ofthe skin and subcutaneous systems	KP	Drug-susceptible infection	80	270	12
Deaths	Western Europe	Both	All Ages	All causes	2019	Number	Bacterial infections ofthe skin and subcutaneous systems	KP	No infection	324	1106	48
Deaths	Southern Latin America	Both	All Ages	All causes	2019	Number	Bacterial infections ofthe skin and subcutaneous systems	KP	Drug-susceptible infection	22	73	4
Deaths	Southern Latin America	Both	All Ages	All causes	2019	Number	Bacterial infections ofthe skin and subcutaneous systems	KP	No infection	75	244	13
Deaths	High-income North America	Both	All Ages	All causes	2019	Number	Bacterial infections ofthe skin and subcutaneous systems	KP	Drug-susceptible infection	35	114	5
Deaths	High-income North America	Both	All Ages	All causes	2019	Number	Bacterial infections ofthe skin and subcutaneous systems	KP	No infection	144	468	24
Deaths	Caribbean	Both	All Ages	All causes	2019	Number	Bacterial infections ofthe skin and subcutaneous systems	KP	Drug-susceptible infection	17	59	3
Deaths	Caribbean	Both	All Ages	All causes	2019	Number	Bacterial infections ofthe skin and subcutaneous systems	KP	No infection	63	207	10
Deaths	Andean Latin America	Both	All Ages	All causes	2019	Number	Bacterial infections ofthe skin and subcutaneous systems	KP	Drug-susceptible infection	9	33	1
Deaths	Andean Latin America	Both	All Ages	All causes	2019	Number	Bacterial infections ofthe skin and subcutaneous systems	KP	No infection	33	122	4
Deaths	Central Latin America	Both	All Ages	All causes	2019	Number	Bacterial infections ofthe skin and subcutaneous systems	KP	Drug-susceptible infection	50	173	8
Deaths	Central Latin America	Both	All Ages	All causes	2019	Number	Bacterial infections ofthe skin and subcutaneous systems	KP	No infection	184	657	30
Deaths	Tropical Latin America	Both	All Ages	All causes	2019	Number	Bacterial infections ofthe skin and subcutaneous systems	KP	Drug-susceptible infection	64	221	10
Deaths	Tropical Latin America	Both	All Ages	All causes	2019	Number	Bacterial infections ofthe skin and subcutaneous systems	KP	No infection	215	724	38
Deaths	North Africa and Middle East	Both	All Ages	All causes	2019	Number	Bacterial infections ofthe skin and subcutaneous systems	KP	Drug-susceptible infection	90	340	11
Deaths	North Africa and Middle East	Both	All Ages	All causes	2019	Number	Bacterial infections ofthe skin and subcutaneous systems	KP	No infection	295	1132	35
Deaths	South Asia	Both	All Ages	All causes	2019	Number	Bacterial infections ofthe skin and subcutaneous systems	KP	Drug-susceptible infection	506	2026	53
Deaths	South Asia	Both	All Ages	All causes	2019	Number	Bacterial infections ofthe skin and subcutaneous systems	KP	No infection	1413	5552	148
Deaths	Central Sub-Saharan Africa	Both	All Ages	All causes	2019	Number	Bacterial infections ofthe skin and subcutaneous systems	KP	Drug-susceptible infection	22	92	2
Deaths	Central Sub-Saharan Africa	Both	All Ages	All causes	2019	Number	Bacterial infections ofthe skin and subcutaneous systems	KP	No infection	83	350	7
Deaths	Eastern Sub-Saharan Africa	Both	All Ages	All causes	2019	Number	Bacterial infections ofthe skin and subcutaneous systems	KP	Drug-susceptible infection	72	299	7
Deaths	Eastern Sub-Saharan Africa	Both	All Ages	All causes	2019	Number	Bacterial infections ofthe skin and subcutaneous systems	KP	No infection	266	1081	24
Deaths	Southern Sub-Saharan Africa	Both	All Ages	All causes	2019	Number	Bacterial infections ofthe skin and subcutaneous systems	KP	Drug-susceptible infection	23	89	3
Deaths	Southern Sub-Saharan Africa	Both	All Ages	All causes	2019	Number	Bacterial infections ofthe skin and subcutaneous systems	KP	No infection	83	319	10
Deaths	Western Sub-Saharan Africa	Both	All Ages	All causes	2019	Number	Bacterial infections ofthe skin and subcutaneous systems	KP	Drug-susceptible infection	86	348	8
Deaths	Western Sub-Saharan Africa	Both	All Ages	All causes	2019	Number	Bacterial infections ofthe skin and subcutaneous systems	KP	No infection	313	1244	29
Deaths	Global	Both	All Ages	All causes	2019	Number	Bloodstream infections	KP	Drug-susceptible infection	64308	104487	36134
Deaths	Global	Both	All Ages	All causes	2019	Number	Bloodstream infections	KP	No infection	214528	335332	128753
Deaths	East Asia	Both	All Ages	All causes	2019	Number	Bloodstream infections	KP	Drug-susceptible infection	6104	11728	2763
Deaths	East Asia	Both	All Ages	All causes	2019	Number	Bloodstream infections	KP	No infection	22229	37953	11979

Deaths	Southeast Asia	Both	All Ages	All causes	2019	Number	Bloodstream infections	KP	Drug-susceptible infection	4754	8021	2562
Deaths	Southeast Asia	Both	All Ages	All causes	2019	Number	Bloodstream infections	KP	No infection	15385	24924	8868
Deaths	Oceania	Both	All Ages	All causes	2019	Number	Bloodstream infections	KP	Drug-susceptible infection	116	204	58
Deaths	Oceania	Both	All Ages	All causes	2019	Number	Bloodstream infections	KP	No infection	420	659	243
Deaths	Central Asia	Both	All Ages	All causes	2019	Number	Bloodstream infections	KP	Drug-susceptible infection	735	1316	374
Deaths	Central Asia	Both	All Ages	All causes	2019	Number	Bloodstream infections	KP	No infection	2712	4548	1516
Deaths	Central Europe	Both	All Ages	All causes	2019	Number	Bloodstream infections	KP	Drug-susceptible infection	1245	2216	600
Deaths	Central Europe	Both	All Ages	All causes	2019	Number	Bloodstream infections	KP	No infection	4319	7453	2288
Deaths	Eastern Europe	Both	All Ages	All causes	2019	Number	Bloodstream infections	KP	Drug-susceptible infection	2910	5084	1527
Deaths	Eastern Europe	Both	All Ages	All causes	2019	Number	Bloodstream infections	KP	No infection	8758	14765	4640
Deaths	High-income Asia Pacific	Both	All Ages	All causes	2019	Number	Bloodstream infections	KP	Drug-susceptible infection	476	826	246
Deaths	High-income Asia Pacific	Both	All Ages	All causes	2019	Number	Bloodstream infections	KP	No infection	2046	3357	1135
Deaths	Australasia	Both	All Ages	All causes	2019	Number	Bloodstream infections	KP	Drug-susceptible infection	55	99	28
Deaths	Australasia	Both	All Ages	All causes	2019	Number	Bloodstream infections	KP	No infection	260	447	138
Deaths	Western Europe	Both	All Ages	All causes	2019	Number	Bloodstream infections	KP	Drug-susceptible infection	1953	3394	994
Deaths	Western Europe	Both	All Ages	All causes	2019	Number	Bloodstream infections	KP	No infection	7795	13165	4154
Deaths	Southern Latin America	Both	All Ages	All causes	2019	Number	Bloodstream infections	KP	Drug-susceptible infection	523	927	265
Deaths	Southern Latin America	Both	All Ages	All causes	2019	Number	Bloodstream infections	KP	No infection	1822	3079	991
Deaths	High-income North America	Both	All Ages	All causes	2019	Number	Bloodstream infections	KP	Drug-susceptible infection	953	1681	485
Deaths	High-income North America	Both	All Ages	All causes	2019	Number	Bloodstream infections	KP	No infection	3956	6702	2140
Deaths	Caribbean	Both	All Ages	All causes	2019	Number	Bloodstream infections	KP	Drug-susceptible infection	395	679	206
Deaths	Caribbean	Both	All Ages	All causes	2019	Number	Bloodstream infections	KP	No infection	1519	2379	876
Deaths	Andean Latin America	Both	All Ages	All causes	2019	Number	Bloodstream infections	KP	Drug-susceptible infection	556	923	306
Deaths	Andean Latin America	Both	All Ages	All causes	2019	Number	Bloodstream infections	KP	No infection	2041	3186	1180
Deaths	Central Latin America	Both	All Ages	All causes	2019	Number	Bloodstream infections	KP	Drug-susceptible infection	1678	2882	871
Deaths	Central Latin America	Both	All Ages	All causes	2019	Number	Bloodstream infections	KP	No infection	6239	10049	3449
Deaths	Tropical Latin America	Both	All Ages	All causes	2019	Number	Bloodstream infections	KP	Drug-susceptible infection	1849	3047	1004
Deaths	Tropical Latin America	Both	All Ages	All causes	2019	Number	Bloodstream infections	KP	No infection	6213	10157	3580
Deaths	North Africa and Middle East	Both	All Ages	All causes	2019	Number	Bloodstream infections	KP	Drug-susceptible infection	4016	6768	2150
Deaths	North Africa and Middle East	Both	All Ages	All causes	2019	Number	Bloodstream infections	KP	No infection	13387	21301	7627
Deaths	South Asia	Both	All Ages	All causes	2019	Number	Bloodstream infections	KP	Drug-susceptible infection	20796	33393	11683
Deaths	South Asia	Both	All Ages	All causes	2019	Number	Bloodstream infections	KP	No infection	59573	95764	34167
Deaths	Central Sub-Saharan Africa	Both	All Ages	All causes	2019	Number	Bloodstream infections	KP	Drug-susceptible infection	1359	2224	756
Deaths	Central Sub-Saharan Africa	Both	All Ages	All causes	2019	Number	Bloodstream infections	KP	No infection	5223	7641	3444
Deaths	Eastern Sub-Saharan Africa	Both	All Ages	All causes	2019	Number	Bloodstream infections	KP	Drug-susceptible infection	5653	8800	3418
Deaths	Eastern Sub-Saharan Africa	Both	All Ages	All causes	2019	Number	Bloodstream infections	KP	No infection	20877	30247	13824
Deaths	Southern Sub-Saharan Africa	Both	All Ages	All causes	2019	Number	Bloodstream infections	KP	Drug-susceptible infection	1036	1658	599
Deaths	Southern Sub-Saharan Africa	Both	All Ages	All causes	2019	Number	Bloodstream infections	KP	No infection	3750	5524	2440
Deaths	Western Sub-Saharan Africa	Both	All Ages	All causes	2019	Number	Bloodstream infections	KP	Drug-susceptible infection	7144	11004	4263
Deaths	Western Sub-Saharan Africa	Both	All Ages	All causes	2019	Number	Bloodstream infections	KP	No infection	26013	37682	17165
Deaths	Global	Both	All Ages	All causes	2019	Number	Endocarditis and other cardiac infections	KP	Drug-susceptible infection	2271	3316	1525
Deaths	Global	Both	All Ages	All causes	2019	Number	Endocarditis and other cardiac infections	KP	No infection	7777	10805	5707
Deaths	East Asia	Both	All Ages	All causes	2019	Number	Endocarditis and other cardiac infections	KP	Drug-susceptible infection	182	339	89
Deaths	East Asia	Both	All Ages	All causes	2019	Number	Endocarditis and other cardiac infections	KP	No infection	657	1102	368
Deaths	Southeast Asia	Both	All Ages	All causes	2019	Number	Endocarditis and other cardiac infections	KP	Drug-susceptible infection	191	276	134
Deaths	Southeast Asia	Both	All Ages	All causes	2019	Number	Endocarditis and other cardiac infections	KP	No infection	624	837	471
Deaths	Oceania	Both	All Ages	All causes	2019	Number	Endocarditis and other cardiac infections	KP	Drug-susceptible infection	3	5	2
Deaths	Oceania	Both	All Ages	All causes	2019	Number	Endocarditis and other cardiac infections	KP	No infection	13	18	9

Deaths	Central Asia	Both	All Ages	All causes	2019	Number	Endocarditis and other cardiac infections	KP	Drug-susceptible infection	21	36	11
Deaths	Central Asia	Both	All Ages	All causes	2019	Number	Endocarditis and other cardiac infections	KP	No infection	76	123	45
Deaths	Central Europe	Both	All Ages	All causes	2019	Number	Endocarditis and other cardiac infections	KP	Drug-susceptible infection	127	229	65
Deaths	Central Europe	Both	All Ages	All causes	2019	Number	Endocarditis and other cardiac infections	KP	No infection	447	740	245
Deaths	Eastern Europe	Both	All Ages	All causes	2019	Number	Endocarditis and other cardiac infections	KP	Drug-susceptible infection	181	298	106
Deaths	Eastern Europe	Both	All Ages	All causes	2019	Number	Endocarditis and other cardiac infections	KP	No infection	564	930	335
Deaths	High-income Asia Pacific	Both	All Ages	All causes	2019	Number	Endocarditis and other cardiac infections	KP	Drug-susceptible infection	44	68	24
Deaths	High-income Asia Pacific	Both	All Ages	All causes	2019	Number	Endocarditis and other cardiac infections	KP	No infection	196	273	114
Deaths	Australasia	Both	All Ages	All causes	2019	Number	Endocarditis and other cardiac infections	KP	Drug-susceptible infection	5	8	3
Deaths	Australasia	Both	All Ages	All causes	2019	Number	Endocarditis and other cardiac infections	KP	No infection	25	36	16
Deaths	Western Europe	Both	All Ages	All causes	2019	Number	Endocarditis and other cardiac infections	KP	Drug-susceptible infection	273	407	151
Deaths	Western Europe	Both	All Ages	All causes	2019	Number	Endocarditis and other cardiac infections	KP	No infection	1109	1533	694
Deaths	Southern Latin America	Both	All Ages	All causes	2019	Number	Endocarditis and other cardiac infections	KP	Drug-susceptible infection	55	76	38
Deaths	Southern Latin America	Both	All Ages	All causes	2019	Number	Endocarditis and other cardiac infections	KP	No infection	190	238	149
Deaths	High-income North America	Both	All Ages	All causes	2019	Number	Endocarditis and other cardiac infections	KP	Drug-susceptible infection	104	153	58
Deaths	High-income North America	Both	All Ages	All causes	2019	Number	Endocarditis and other cardiac infections	KP	No infection	433	574	265
Deaths	Caribbean	Both	All Ages	All causes	2019	Number	Endocarditis and other cardiac infections	KP	Drug-susceptible infection	13	19	8
Deaths	Caribbean	Both	All Ages	All causes	2019	Number	Endocarditis and other cardiac infections	KP	No infection	48	63	36
Deaths	Andean Latin America	Both	All Ages	All causes	2019	Number	Endocarditis and other cardiac infections	KP	Drug-susceptible infection	13	19	9
Deaths	Andean Latin America	Both	All Ages	All causes	2019	Number	Endocarditis and other cardiac infections	KP	No infection	48	64	35
Deaths	Central Latin America	Both	All Ages	All causes	2019	Number	Endocarditis and other cardiac infections	KP	Drug-susceptible infection	46	70	30
Deaths	Central Latin America	Both	All Ages	All causes	2019	Number	Endocarditis and other cardiac infections	KP	No infection	170	235	123
Deaths	Tropical Latin America	Both	All Ages	All causes	2019	Number	Endocarditis and other cardiac infections	KP	Drug-susceptible infection	103	139	72
Deaths	Tropical Latin America	Both	All Ages	All causes	2019	Number	Endocarditis and other cardiac infections	KP	No infection	345	436	258
Deaths	North Africa and Middle East	Both	All Ages	All causes	2019	Number	Endocarditis and other cardiac infections	KP	Drug-susceptible infection	151	228	98
Deaths	North Africa and Middle East	Both	All Ages	All causes	2019	Number	Endocarditis and other cardiac infections	KP	No infection	495	708	343
Deaths	South Asia	Both	All Ages	All causes	2019	Number	Endocarditis and other cardiac infections	KP	Drug-susceptible infection	544	775	372
Deaths	South Asia	Both	All Ages	All causes	2019	Number	Endocarditis and other cardiac infections	KP	No infection	1539	2164	1088
Deaths	Central Sub-Saharan Africa	Both	All Ages	All causes	2019	Number	Endocarditis and other cardiac infections	KP	Drug-susceptible infection	24	40	13
Deaths	Central Sub-Saharan Africa	Both	All Ages	All causes	2019	Number	Endocarditis and other cardiac infections	KP	No infection	93	141	60
Deaths	Eastern Sub-Saharan Africa	Both	All Ages	All causes	2019	Number	Endocarditis and other cardiac infections	KP	Drug-susceptible infection	78	130	46
Deaths	Eastern Sub-Saharan Africa	Both	All Ages	All causes	2019	Number	Endocarditis and other cardiac infections	KP	No infection	292	434	196
Deaths	Southern Sub-Saharan Africa	Both	All Ages	All causes	2019	Number	Endocarditis and other cardiac infections	KP	Drug-susceptible infection	25	38	16
Deaths	Southern Sub-Saharan Africa	Both	All Ages	All causes	2019	Number	Endocarditis and other cardiac infections	KP	No infection	90	125	66
Deaths	Western Sub-Saharan Africa	Both	All Ages	All causes	2019	Number	Endocarditis and other cardiac infections	KP	Drug-susceptible infection	88	130	58
Deaths	Western Sub-Saharan Africa	Both	All Ages	All causes	2019	Number	Endocarditis and other cardiac infections	KP	No infection	325	439	246
Deaths	Global	Both	All Ages	All causes	2019	Number	Infections of bones, joints, and related organs	KP	Drug-susceptible infection	349	861	93
Deaths	Global	Both	All Ages	All causes	2019	Number	Infections of bones, joints, and related organs	KP	No infection	1121	2664	312
Deaths	East Asia	Both	All Ages	All causes	2019	Number	Infections of bones, joints, and related organs	KP	Drug-susceptible infection	34	83	9
Deaths	East Asia	Both	All Ages	All causes	2019	Number	Infections of bones, joints, and related organs	KP	No infection	123	289	37
Deaths	Southeast Asia	Both	All Ages	All causes	2019	Number	Infections of bones, joints, and related organs	KP	Drug-susceptible infection	25	65	6
Deaths	Southeast Asia	Both	All Ages	All causes	2019	Number	Infections of bones, joints, and related organs	KP	No infection	80	195	22
Deaths	Oceania	Both	All Ages	All causes	2019	Number	Infections of bones, joints, and related organs	KP	Drug-susceptible infection	0	1	0
Deaths	Oceania	Both	All Ages	All causes	2019	Number	Infections of bones, joints, and related organs	KP	No infection	2	5	0
Deaths	Central Asia	Both	All Ages	All causes	2019	Number	Infections of bones, joints, and related organs	KP	Drug-susceptible infection	2	6	1
Deaths	Central Asia	Both	All Ages	All causes	2019	Number	Infections of bones, joints, and related organs	KP	No infection	8	20	2
Deaths	Central Europe	Both	All Ages	All causes	2019	Number	Infections of bones, joints, and related organs	KP	Drug-susceptible infection	4	10	1
Deaths	Central Europe	Both	All Ages	All causes	2019	Number	Infections of bones, joints, and related organs	KP	No infection	14	34	4

Deaths	Eastern Europe	Both	All Ages	All causes	2019	Number	Infections of bones, joints, and related organs	KP	Drug-susceptible infection	12	28	3
Deaths	Eastern Europe	Both	All Ages	All causes	2019	Number	Infections of bones, joints, and related organs	KP	No infection	36	85	10
Deaths	High-income Asia Pacific	Both	All Ages	All causes	2019	Number	Infections of bones, joints, and related organs	KP	Drug-susceptible infection	2	4	1
Deaths	High-income Asia Pacific	Both	All Ages	All causes	2019	Number	Infections of bones, joints, and related organs	KP	No infection	8	19	3
Deaths	Australasia	Both	All Ages	All causes	2019	Number	Infections of bones, joints, and related organs	KP	Drug-susceptible infection	0	1	0
Deaths	Australasia	Both	All Ages	All causes	2019	Number	Infections of bones, joints, and related organs	KP	No infection	1	3	0
Deaths	Western Europe	Both	All Ages	All causes	2019	Number	Infections of bones, joints, and related organs	KP	Drug-susceptible infection	8	18	2
Deaths	Western Europe	Both	All Ages	All causes	2019	Number	Infections of bones, joints, and related organs	KP	No infection	32	71	10
Deaths	Southern Latin America	Both	All Ages	All causes	2019	Number	Infections of bones, joints, and related organs	KP	Drug-susceptible infection	2	5	1
Deaths	Southern Latin America	Both	All Ages	All causes	2019	Number	Infections of bones, joints, and related organs	KP	No infection	7	16	2
Deaths	High-income North America	Both	All Ages	All causes	2019	Number	Infections of bones, joints, and related organs	KP	Drug-susceptible infection	5	11	1
Deaths	High-income North America	Both	All Ages	All causes	2019	Number	Infections of bones, joints, and related organs	KP	No infection	19	43	6
Deaths	Caribbean	Both	All Ages	All causes	2019	Number	Infections of bones, joints, and related organs	KP	Drug-susceptible infection	2	6	1
Deaths	Caribbean	Both	All Ages	All causes	2019	Number	Infections of bones, joints, and related organs	KP	No infection	8	20	2
Deaths	Andean Latin America	Both	All Ages	All causes	2019	Number	Infections of bones, joints, and related organs	KP	Drug-susceptible infection	2	5	1
Deaths	Andean Latin America	Both	All Ages	All causes	2019	Number	Infections of bones, joints, and related organs	KP	No infection	7	17	2
Deaths	Central Latin America	Both	All Ages	All causes	2019	Number	Infections of bones, joints, and related organs	KP	Drug-susceptible infection	7	19	2
Deaths	Central Latin America	Both	All Ages	All causes	2019	Number	Infections of bones, joints, and related organs	KP	No infection	27	65	8
Deaths	Tropical Latin America	Both	All Ages	All causes	2019	Number	Infections of bones, joints, and related organs	KP	Drug-susceptible infection	8	20	2
Deaths	Tropical Latin America	Both	All Ages	All causes	2019	Number	Infections of bones, joints, and related organs	KP	No infection	27	65	7
Deaths	North Africa and Middle East	Both	All Ages	All causes	2019	Number	Infections of bones, joints, and related organs	KP	Drug-susceptible infection	30	76	7
Deaths	North Africa and Middle East	Both	All Ages	All causes	2019	Number	Infections of bones, joints, and related organs	KP	No infection	101	244	25
Deaths	South Asia	Both	All Ages	All causes	2019	Number	Infections of bones, joints, and related organs	KP	Drug-susceptible infection	148	354	40
Deaths	South Asia	Both	All Ages	All causes	2019	Number	Infections of bones, joints, and related organs	KP	No infection	409	968	108
Deaths	Central Sub-Saharan Africa	Both	All Ages	All causes	2019	Number	Infections of bones, joints, and related organs	KP	Drug-susceptible infection	7	18	2
Deaths	Central Sub-Saharan Africa	Both	All Ages	All causes	2019	Number	Infections of bones, joints, and related organs	KP	No infection	26	65	7
Deaths	Eastern Sub-Saharan Africa	Both	All Ages	All causes	2019	Number	Infections of bones, joints, and related organs	KP	Drug-susceptible infection	23	59	7
Deaths	Eastern Sub-Saharan Africa	Both	All Ages	All causes	2019	Number	Infections of bones, joints, and related organs	KP	No infection	83	191	23
Deaths	Southern Sub-Saharan Africa	Both	All Ages	All causes	2019	Number	Infections of bones, joints, and related organs	KP	Drug-susceptible infection	5	13	1
Deaths	Southern Sub-Saharan Africa	Both	All Ages	All causes	2019	Number	Infections of bones, joints, and related organs	KP	No infection	17	40	5
Deaths	Western Sub-Saharan Africa	Both	All Ages	All causes	2019	Number	Infections of bones, joints, and related organs	KP	Drug-susceptible infection	23	61	6
Deaths	Western Sub-Saharan Africa	Both	All Ages	All causes	2019	Number	Infections of bones, joints, and related organs	KP	No infection	84	199	22
Deaths	Global	Both	All Ages	All causes	2019	Number	LRIs and all related infections in the thorax	KP	Drug-susceptible infection	69295	93725	50546
Deaths	Global	Both	All Ages	All causes	2019	Number	LRIs and all related infections in the thorax	KP	No infection	231776	288677	184551
Deaths	East Asia	Both	All Ages	All causes	2019	Number	LRIs and all related infections in the thorax	KP	Drug-susceptible infection	3136	5373	1686
Deaths	East Asia	Both	All Ages	All causes	2019	Number	LRIs and all related infections in the thorax	KP	No infection	11392	16946	7485
Deaths	Southeast Asia	Both	All Ages	All causes	2019	Number	LRIs and all related infections in the thorax	KP	Drug-susceptible infection	4894	6764	3442
Deaths	Southeast Asia	Both	All Ages	All causes	2019	Number	LRIs and all related infections in the thorax	KP	No infection	15923	20652	12363
Deaths	Oceania	Both	All Ages	All causes	2019	Number	LRIs and all related infections in the thorax	KP	Drug-susceptible infection	219	341	126
Deaths	Oceania	Both	All Ages	All causes	2019	Number	LRIs and all related infections in the thorax	KP	No infection	793	1097	567
Deaths	Central Asia	Both	All Ages	All causes	2019	Number	LRIs and all related infections in the thorax	KP	Drug-susceptible infection	602	862	394
Deaths	Central Asia	Both	All Ages	All causes	2019	Number	LRIs and all related infections in the thorax	KP	No infection	2218	2842	1738
Deaths	Central Europe	Both	All Ages	All causes	2019	Number	LRIs and all related infections in the thorax	KP	Drug-susceptible infection	603	855	416
Deaths	Central Europe	Both	All Ages	All causes	2019	Number	LRIs and all related infections in the thorax	KP	No infection	2105	2825	1584
Deaths	Eastern Europe	Both	All Ages	All causes	2019	Number	LRIs and all related infections in the thorax	KP	Drug-susceptible infection	1375	1961	954
Deaths	Eastern Europe	Both	All Ages	All causes	2019	Number	LRIs and all related infections in the thorax	KP	No infection	4080	5654	2966
Deaths	High-income Asia Pacific	Both	All Ages	All causes	2019	Number	LRIs and all related infections in the thorax	KP	Drug-susceptible infection	538	747	373
Deaths	High-income Asia Pacific	Both	All Ages	All causes	2019	Number	LRIs and all related infections in the thorax	KP	No infection	2399	2843	1958

Deaths	Australasia	Both	All Ages	All causes	2019	Number	LRIs and all related infections in the thorax	KP	Drug-susceptible infection	30	45	20
Deaths	Australasia	Both	All Ages	All causes	2019	Number	LRIs and all related infections in the thorax	KP	No infection	143	195	105
Deaths	Western Europe	Both	All Ages	All causes	2019	Number	LRIs and all related infections in the thorax	KP	Drug-susceptible infection	1278	1735	916
Deaths	Western Europe	Both	All Ages	All causes	2019	Number	LRIs and all related infections in the thorax	KP	No infection	5210	6445	4218
Deaths	Southern Latin America	Both	All Ages	All causes	2019	Number	LRIs and all related infections in the thorax	KP	Drug-susceptible infection	725	959	532
Deaths	Southern Latin America	Both	All Ages	All causes	2019	Number	LRIs and all related infections in the thorax	KP	No infection	2512	2953	2130
Deaths	High-income North America	Both	All Ages	All causes	2019	Number	LRIs and all related infections in the thorax	KP	Drug-susceptible infection	637	911	431
Deaths	High-income North America	Both	All Ages	All causes	2019	Number	LRIs and all related infections in the thorax	KP	No infection	2648	3559	1983
Deaths	Caribbean	Both	All Ages	All causes	2019	Number	LRIs and all related infections in the thorax	KP	Drug-susceptible infection	396	585	253
Deaths	Caribbean	Both	All Ages	All causes	2019	Number	LRIs and all related infections in the thorax	KP	No infection	1508	1977	1125
Deaths	Andean Latin America	Both	All Ages	All causes	2019	Number	LRIs and all related infections in the thorax	KP	Drug-susceptible infection	697	998	463
Deaths	Andean Latin America	Both	All Ages	All causes	2019	Number	LRIs and all related infections in the thorax	KP	No infection	2582	3306	2005
Deaths	Central Latin America	Both	All Ages	All causes	2019	Number	LRIs and all related infections in the thorax	KP	Drug-susceptible infection	1178	1748	770
Deaths	Central Latin America	Both	All Ages	All causes	2019	Number	LRIs and all related infections in the thorax	KP	No infection	4401	5968	3215
Deaths	Tropical Latin America	Both	All Ages	All causes	2019	Number	LRIs and all related infections in the thorax	KP	Drug-susceptible infection	2045	2690	1510
Deaths	Tropical Latin America	Both	All Ages	All causes	2019	Number	LRIs and all related infections in the thorax	KP	No infection	6864	8531	5565
Deaths	North Africa and Middle East	Both	All Ages	All causes	2019	Number	LRIs and all related infections in the thorax	KP	Drug-susceptible infection	3525	4927	2364
Deaths	North Africa and Middle East	Both	All Ages	All causes	2019	Number	LRIs and all related infections in the thorax	KP	No infection	11900	15420	8994
Deaths	South Asia	Both	All Ages	All causes	2019	Number	LRIs and all related infections in the thorax	KP	Drug-susceptible infection	22914	30145	17042
Deaths	South Asia	Both	All Ages	All causes	2019	Number	LRIs and all related infections in the thorax	KP	No infection	64980	84021	49662
Deaths	Central Sub-Saharan Africa	Both	All Ages	All causes	2019	Number	LRIs and all related infections in the thorax	KP	Drug-susceptible infection	2558	3735	1613
Deaths	Central Sub-Saharan Africa	Both	All Ages	All causes	2019	Number	LRIs and all related infections in the thorax	KP	No infection	9840	12674	7550
Deaths	Eastern Sub-Saharan Africa	Both	All Ages	All causes	2019	Number	LRIs and all related infections in the thorax	KP	Drug-susceptible infection	7759	10953	5228
Deaths	Eastern Sub-Saharan Africa	Both	All Ages	All causes	2019	Number	LRIs and all related infections in the thorax	KP	No infection	28847	36203	22932
Deaths	Southern Sub-Saharan Africa	Both	All Ages	All causes	2019	Number	LRIs and all related infections in the thorax	KP	Drug-susceptible infection	1377	1956	923
Deaths	Southern Sub-Saharan Africa	Both	All Ages	All causes	2019	Number	LRIs and all related infections in the thorax	KP	No infection	5035	6167	4137
Deaths	Western Sub-Saharan Africa	Both	All Ages	All causes	2019	Number	LRIs and all related infections in the thorax	KP	Drug-susceptible infection	12808	17752	8663
Deaths	Western Sub-Saharan Africa	Both	All Ages	All causes	2019	Number	LRIs and all related infections in the thorax	KP	No infection	46409	58151	36643
Deaths	Global	Both	All Ages	All causes	2019	Number	Meningitis and other bacterial CNS infections	KP	Drug-susceptible infection	8974	13636	5847
Deaths	Global	Both	All Ages	All causes	2019	Number	Meningitis and other bacterial CNS infections	KP	No infection	30318	42755	21378
Deaths	East Asia	Both	All Ages	All causes	2019	Number	Meningitis and other bacterial CNS infections	KP	Drug-susceptible infection	235	482	114
Deaths	East Asia	Both	All Ages	All causes	2019	Number	Meningitis and other bacterial CNS infections	KP	No infection	857	1669	484
Deaths	Southeast Asia	Both	All Ages	All causes	2019	Number	Meningitis and other bacterial CNS infections	KP	Drug-susceptible infection	370	586	238
Deaths	Southeast Asia	Both	All Ages	All causes	2019	Number	Meningitis and other bacterial CNS infections	KP	No infection	1209	1829	832
Deaths	Oceania	Both	All Ages	All causes	2019	Number	Meningitis and other bacterial CNS infections	KP	Drug-susceptible infection	17	30	9
Deaths	Oceania	Both	All Ages	All causes	2019	Number	Meningitis and other bacterial CNS infections	KP	No infection	62	98	38
Deaths	Central Asia	Both	All Ages	All causes	2019	Number	Meningitis and other bacterial CNS infections	KP	Drug-susceptible infection	26	49	15
Deaths	Central Asia	Both	All Ages	All causes	2019	Number	Meningitis and other bacterial CNS infections	KP	No infection	98	171	62
Deaths	Central Europe	Both	All Ages	All causes	2019	Number	Meningitis and other bacterial CNS infections	KP	Drug-susceptible infection	25	50	13
Deaths	Central Europe	Both	All Ages	All causes	2019	Number	Meningitis and other bacterial CNS infections	KP	No infection	86	172	47
Deaths	Eastern Europe	Both	All Ages	All causes	2019	Number	Meningitis and other bacterial CNS infections	KP	Drug-susceptible infection	83	145	52
Deaths	Eastern Europe	Both	All Ages	All causes	2019	Number	Meningitis and other bacterial CNS infections	KP	No infection	253	438	165
Deaths	High-income Asia Pacific	Both	All Ages	All causes	2019	Number	Meningitis and other bacterial CNS infections	KP	Drug-susceptible infection	7	14	4
Deaths	High-income Asia Pacific	Both	All Ages	All causes	2019	Number	Meningitis and other bacterial CNS infections	KP	No infection	30	59	18
Deaths	Australasia	Both	All Ages	All causes	2019	Number	Meningitis and other bacterial CNS infections	KP	Drug-susceptible infection	1	2	1
Deaths	Australasia	Both	All Ages	All causes	2019	Number	Meningitis and other bacterial CNS infections	KP	No infection	5	10	3
Deaths	Western Europe	Both	All Ages	All causes	2019	Number	Meningitis and other bacterial CNS infections	KP	Drug-susceptible infection	32	60	19
Deaths	Western Europe	Both	All Ages	All causes	2019	Number	Meningitis and other bacterial CNS infections	KP	No infection	130	236	84

Deaths	Southern Latin America	Both	All Ages	All causes	2019	Number	Meningitis and other bacterial CNS infections	KP	Drug-susceptible infection	17	27	11
Deaths	Southern Latin America	Both	All Ages	All causes	2019	Number	Meningitis and other bacterial CNS infections	KP	No infection	59	89	43
Deaths	High-income North America	Both	All Ages	All causes	2019	Number	Meningitis and other bacterial CNS infections	KP	Drug-susceptible infection	18	34	11
Deaths	High-income North America	Both	All Ages	All causes	2019	Number	Meningitis and other bacterial CNS infections	KP	No infection	76	140	48
Deaths	Caribbean	Both	All Ages	All causes	2019	Number	Meningitis and other bacterial CNS infections	KP	Drug-susceptible infection	61	105	32
Deaths	Caribbean	Both	All Ages	All causes	2019	Number	Meningitis and other bacterial CNS infections	KP	No infection	230	374	140
Deaths	Andean Latin America	Both	All Ages	All causes	2019	Number	Meningitis and other bacterial CNS infections	KP	Drug-susceptible infection	16	28	9
Deaths	Andean Latin America	Both	All Ages	All causes	2019	Number	Meningitis and other bacterial CNS infections	KP	No infection	57	97	36
Deaths	Central Latin America	Both	All Ages	All causes	2019	Number	Meningitis and other bacterial CNS infections	KP	Drug-susceptible infection	49	85	29
Deaths	Central Latin America	Both	All Ages	All causes	2019	Number	Meningitis and other bacterial CNS infections	KP	No infection	183	303	122
Deaths	Tropical Latin America	Both	All Ages	All causes	2019	Number	Meningitis and other bacterial CNS infections	KP	Drug-susceptible infection	62	96	42
Deaths	Tropical Latin America	Both	All Ages	All causes	2019	Number	Meningitis and other bacterial CNS infections	KP	No infection	209	315	147
Deaths	North Africa and Middle East	Both	All Ages	All causes	2019	Number	Meningitis and other bacterial CNS infections	KP	Drug-susceptible infection	361	675	194
Deaths	North Africa and Middle East	Both	All Ages	All causes	2019	Number	Meningitis and other bacterial CNS infections	KP	No infection	1228	2242	710
Deaths	South Asia	Both	All Ages	All causes	2019	Number	Meningitis and other bacterial CNS infections	KP	Drug-susceptible infection	2520	3666	1746
Deaths	South Asia	Both	All Ages	All causes	2019	Number	Meningitis and other bacterial CNS infections	KP	No infection	7165	10283	5130
Deaths	Central Sub-Saharan Africa	Both	All Ages	All causes	2019	Number	Meningitis and other bacterial CNS infections	KP	Drug-susceptible infection	287	461	165
Deaths	Central Sub-Saharan Africa	Both	All Ages	All causes	2019	Number	Meningitis and other bacterial CNS infections	KP	No infection	1107	1636	742
Deaths	Eastern Sub-Saharan Africa	Both	All Ages	All causes	2019	Number	Meningitis and other bacterial CNS infections	KP	Drug-susceptible infection	1385	2112	846
Deaths	Eastern Sub-Saharan Africa	Both	All Ages	All causes	2019	Number	Meningitis and other bacterial CNS infections	KP	No infection	5039	6979	3593
Deaths	Southern Sub-Saharan Africa	Both	All Ages	All causes	2019	Number	Meningitis and other bacterial CNS infections	KP	Drug-susceptible infection	116	170	72
Deaths	Southern Sub-Saharan Africa	Both	All Ages	All causes	2019	Number	Meningitis and other bacterial CNS infections	KP	No infection	424	568	314
Deaths	Western Sub-Saharan Africa	Both	All Ages	All causes	2019	Number	Meningitis and other bacterial CNS infections	KP	Drug-susceptible infection	3286	5470	1846
Deaths	Western Sub-Saharan Africa	Both	All Ages	All causes	2019	Number	Meningitis and other bacterial CNS infections	KP	No infection	11809	17805	7646
Deaths	Global	Both	All Ages	All causes	2019	Number	Peritoneal and intra-abdominal infections	KP	Drug-susceptible infection	37447	57239	23574
Deaths	Global	Both	All Ages	All causes	2019	Number	Peritoneal and intra-abdominal infections	KP	No infection	123158	182226	80452
Deaths	East Asia	Both	All Ages	All causes	2019	Number	Peritoneal and intra-abdominal infections	KP	Drug-susceptible infection	3563	6605	1738
Deaths	East Asia	Both	All Ages	All causes	2019	Number	Peritoneal and intra-abdominal infections	KP	No infection	12969	21620	7371
Deaths	Southeast Asia	Both	All Ages	All causes	2019	Number	Peritoneal and intra-abdominal infections	KP	Drug-susceptible infection	4099	6271	2479
Deaths	Southeast Asia	Both	All Ages	All causes	2019	Number	Peritoneal and intra-abdominal infections	KP	No infection	13441	19359	8799
Deaths	Oceania	Both	All Ages	All causes	2019	Number	Peritoneal and intra-abdominal infections	KP	Drug-susceptible infection	28	51	13
Deaths	Oceania	Both	All Ages	All causes	2019	Number	Peritoneal and intra-abdominal infections	KP	No infection	100	176	55
Deaths	Central Asia	Both	All Ages	All causes	2019	Number	Peritoneal and intra-abdominal infections	KP	Drug-susceptible infection	514	846	285
Deaths	Central Asia	Both	All Ages	All causes	2019	Number	Peritoneal and intra-abdominal infections	KP	No infection	1900	2857	1193
Deaths	Central Europe	Both	All Ages	All causes	2019	Number	Peritoneal and intra-abdominal infections	KP	Drug-susceptible infection	868	1339	519
Deaths	Central Europe	Both	All Ages	All causes	2019	Number	Peritoneal and intra-abdominal infections	KP	No infection	3025	4496	1933
Deaths	Eastern Europe	Both	All Ages	All causes	2019	Number	Peritoneal and intra-abdominal infections	KP	Drug-susceptible infection	2153	3189	1374
Deaths	Eastern Europe	Both	All Ages	All causes	2019	Number	Peritoneal and intra-abdominal infections	KP	No infection	6532	9470	4227
Deaths	High-income Asia Pacific	Both	All Ages	All causes	2019	Number	Peritoneal and intra-abdominal infections	KP	Drug-susceptible infection	399	652	219
Deaths	High-income Asia Pacific	Both	All Ages	All causes	2019	Number	Peritoneal and intra-abdominal infections	KP	No infection	1725	2610	1068
Deaths	Australasia	Both	All Ages	All causes	2019	Number	Peritoneal and intra-abdominal infections	KP	Drug-susceptible infection	39	62	22
Deaths	Australasia	Both	All Ages	All causes	2019	Number	Peritoneal and intra-abdominal infections	KP	No infection	185	285	116
Deaths	Western Europe	Both	All Ages	All causes	2019	Number	Peritoneal and intra-abdominal infections	KP	Drug-susceptible infection	1640	2480	987
Deaths	Western Europe	Both	All Ages	All causes	2019	Number	Peritoneal and intra-abdominal infections	KP	No infection	6618	9614	4408
Deaths	Southern Latin America	Both	All Ages	All causes	2019	Number	Peritoneal and intra-abdominal infections	KP	Drug-susceptible infection	416	627	257
Deaths	Southern Latin America	Both	All Ages	All causes	2019	Number	Peritoneal and intra-abdominal infections	KP	No infection	1451	2094	986
Deaths	High-income North America	Both	All Ages	All causes	2019	Number	Peritoneal and intra-abdominal infections	KP	Drug-susceptible infection	737	1126	440
Deaths	High-income North America	Both	All Ages	All causes	2019	Number	Peritoneal and intra-abdominal infections	KP	No infection	3069	4486	2035

Deaths	Caribbean	Both	All Ages	All causes	2019	Number	Peritoneal and intra-abdominal infections	KP	Drug-susceptible infection	242	402	134
Deaths	Caribbean	Both	All Ages	All causes	2019	Number	Peritoneal and intra-abdominal infections	KP	No infection	919	1406	578
Deaths	Andean Latin America	Both	All Ages	All causes	2019	Number	Peritoneal and intra-abdominal infections	KP	Drug-susceptible infection	389	607	231
Deaths	Andean Latin America	Both	All Ages	All causes	2019	Number	Peritoneal and intra-abdominal infections	KP	No infection	1427	2121	918
Deaths	Central Latin America	Both	All Ages	All causes	2019	Number	Peritoneal and intra-abdominal infections	KP	Drug-susceptible infection	1358	2131	813
Deaths	Central Latin America	Both	All Ages	All causes	2019	Number	Peritoneal and intra-abdominal infections	KP	No infection	5048	7291	3339
Deaths	Tropical Latin America	Both	All Ages	All causes	2019	Number	Peritoneal and intra-abdominal infections	KP	Drug-susceptible infection	1215	1794	785
Deaths	Tropical Latin America	Both	All Ages	All causes	2019	Number	Peritoneal and intra-abdominal infections	KP	No infection	4080	5831	2805
Deaths	North Africa and Middle East	Both	All Ages	All causes	2019	Number	Peritoneal and intra-abdominal infections	KP	Drug-susceptible infection	2765	4316	1631
Deaths	North Africa and Middle East	Both	All Ages	All causes	2019	Number	Peritoneal and intra-abdominal infections	KP	No infection	8533	12979	5218
Deaths	South Asia	Both	All Ages	All causes	2019	Number	Peritoneal and intra-abdominal infections	KP	Drug-susceptible infection	11797	18456	7262
Deaths	South Asia	Both	All Ages	All causes	2019	Number	Peritoneal and intra-abdominal infections	KP	No infection	33011	50014	20782
Deaths	Central Sub-Saharan Africa	Both	All Ages	All causes	2019	Number	Peritoneal and intra-abdominal infections	KP	Drug-susceptible infection	559	956	292
Deaths	Central Sub-Saharan Africa	Both	All Ages	All causes	2019	Number	Peritoneal and intra-abdominal infections	KP	No infection	2150	3464	1285
Deaths	Eastern Sub-Saharan Africa	Both	All Ages	All causes	2019	Number	Peritoneal and intra-abdominal infections	KP	Drug-susceptible infection	2094	3387	1181
Deaths	Eastern Sub-Saharan Africa	Both	All Ages	All causes	2019	Number	Peritoneal and intra-abdominal infections	KP	No infection	7658	11404	4920
Deaths	Southern Sub-Saharan Africa	Both	All Ages	All causes	2019	Number	Peritoneal and intra-abdominal infections	KP	Drug-susceptible infection	347	568	194
Deaths	Southern Sub-Saharan Africa	Both	All Ages	All causes	2019	Number	Peritoneal and intra-abdominal infections	KP	No infection	1259	1901	819
Deaths	Western Sub-Saharan Africa	Both	All Ages	All causes	2019	Number	Peritoneal and intra-abdominal infections	KP	Drug-susceptible infection	2227	3685	1173
Deaths	Western Sub-Saharan Africa	Both	All Ages	All causes	2019	Number	Peritoneal and intra-abdominal infections	KP	No infection	8068	12542	4845
Deaths	Global	Both	All Ages	All causes	2019	Number	Urinary tract infections and pyelonephritis	KP	Drug-susceptible infection	8334	12475	5477
Deaths	Global	Both	All Ages	All causes	2019	Number	Urinary tract infections and pyelonephritis	KP	No infection	27682	40307	19015
Deaths	East Asia	Both	All Ages	All causes	2019	Number	Urinary tract infections and pyelonephritis	KP	Drug-susceptible infection	1011	2099	408
Deaths	East Asia	Both	All Ages	All causes	2019	Number	Urinary tract infections and pyelonephritis	KP	No infection	3659	6958	1681
Deaths	Southeast Asia	Both	All Ages	All causes	2019	Number	Urinary tract infections and pyelonephritis	KP	Drug-susceptible infection	739	1184	428
Deaths	Southeast Asia	Both	All Ages	All causes	2019	Number	Urinary tract infections and pyelonephritis	KP	No infection	2433	3691	1517
Deaths	Oceania	Both	All Ages	All causes	2019	Number	Urinary tract infections and pyelonephritis	KP	Drug-susceptible infection	8	14	4
Deaths	Oceania	Both	All Ages	All causes	2019	Number	Urinary tract infections and pyelonephritis	KP	No infection	28	45	15
Deaths	Central Asia	Both	All Ages	All causes	2019	Number	Urinary tract infections and pyelonephritis	KP	Drug-susceptible infection	127	201	75
Deaths	Central Asia	Both	All Ages	All causes	2019	Number	Urinary tract infections and pyelonephritis	KP	No infection	467	687	319
Deaths	Central Europe	Both	All Ages	All causes	2019	Number	Urinary tract infections and pyelonephritis	KP	Drug-susceptible infection	242	408	131
Deaths	Central Europe	Both	All Ages	All causes	2019	Number	Urinary tract infections and pyelonephritis	KP	No infection	851	1386	501
Deaths	Eastern Europe	Both	All Ages	All causes	2019	Number	Urinary tract infections and pyelonephritis	KP	Drug-susceptible infection	656	1005	418
Deaths	Eastern Europe	Both	All Ages	All causes	2019	Number	Urinary tract infections and pyelonephritis	KP	No infection	1967	2942	1298
Deaths	High-income Asia Pacific	Both	All Ages	All causes	2019	Number	Urinary tract infections and pyelonephritis	KP	Drug-susceptible infection	130	200	80
Deaths	High-income Asia Pacific	Both	All Ages	All causes	2019	Number	Urinary tract infections and pyelonephritis	KP	No infection	573	815	406
Deaths	Australasia	Both	All Ages	All causes	2019	Number	Urinary tract infections and pyelonephritis	KP	Drug-susceptible infection	17	26	11
Deaths	Australasia	Both	All Ages	All causes	2019	Number	Urinary tract infections and pyelonephritis	KP	No infection	81	112	58
Deaths	Western Europe	Both	All Ages	All causes	2019	Number	Urinary tract infections and pyelonephritis	KP	Drug-susceptible infection	652	923	440
Deaths	Western Europe	Both	All Ages	All causes	2019	Number	Urinary tract infections and pyelonephritis	KP	No infection	2681	3589	2001
Deaths	Southern Latin America	Both	All Ages	All causes	2019	Number	Urinary tract infections and pyelonephritis	KP	Drug-susceptible infection	185	261	122
Deaths	Southern Latin America	Both	All Ages	All causes	2019	Number	Urinary tract infections and pyelonephritis	KP	No infection	645	832	473
Deaths	High-income North America	Both	All Ages	All causes	2019	Number	Urinary tract infections and pyelonephritis	KP	Drug-susceptible infection	312	448	213
Deaths	High-income North America	Both	All Ages	All causes	2019	Number	Urinary tract infections and pyelonephritis	KP	No infection	1299	1741	997
Deaths	Caribbean	Both	All Ages	All causes	2019	Number	Urinary tract infections and pyelonephritis	KP	Drug-susceptible infection	49	79	29
Deaths	Caribbean	Both	All Ages	All causes	2019	Number	Urinary tract infections and pyelonephritis	KP	No infection	183	259	127
Deaths	Andean Latin America	Both	All Ages	All causes	2019	Number	Urinary tract infections and pyelonephritis	KP	Drug-susceptible infection	77	121	46
Deaths	Andean Latin America	Both	All Ages	All causes	2019	Number	Urinary tract infections and pyelonephritis	KP	No infection	285	408	189

Deaths	Central Latin America	Both	All Ages	All causes	2019	Number	Urinary tract infections and pyelonephritis	KP	Drug-susceptible infection	309	492	191
Deaths	Central Latin America	Both	All Ages	All causes	2019	Number	Urinary tract infections and pyelonephritis	KP	No infection	1144	1650	784
Deaths	Tropical Latin America	Both	All Ages	All causes	2019	Number	Urinary tract infections and pyelonephritis	KP	Drug-susceptible infection	556	734	381
Deaths	Tropical Latin America	Both	All Ages	All causes	2019	Number	Urinary tract infections and pyelonephritis	KP	No infection	1864	2347	1365
Deaths	North Africa and Middle East	Both	All Ages	All causes	2019	Number	Urinary tract infections and pyelonephritis	KP	Drug-susceptible infection	379	686	184
Deaths	North Africa and Middle East	Both	All Ages	All causes	2019	Number	Urinary tract infections and pyelonephritis	KP	No infection	1223	2107	611
Deaths	South Asia	Both	All Ages	All causes	2019	Number	Urinary tract infections and pyelonephritis	KP	Drug-susceptible infection	2568	3604	1777
Deaths	South Asia	Both	All Ages	All causes	2019	Number	Urinary tract infections and pyelonephritis	KP	No infection	7129	9867	5106
Deaths	Central Sub-Saharan Africa	Both	All Ages	All causes	2019	Number	Urinary tract infections and pyelonephritis	KP	Drug-susceptible infection	33	55	17
Deaths	Central Sub-Saharan Africa	Both	All Ages	All causes	2019	Number	Urinary tract infections and pyelonephritis	KP	No infection	127	195	73
Deaths	Eastern Sub-Saharan Africa	Both	All Ages	All causes	2019	Number	Urinary tract infections and pyelonephritis	KP	Drug-susceptible infection	118	187	64
Deaths	Eastern Sub-Saharan Africa	Both	All Ages	All causes	2019	Number	Urinary tract infections and pyelonephritis	KP	No infection	433	642	233
Deaths	Southern Sub-Saharan Africa	Both	All Ages	All causes	2019	Number	Urinary tract infections and pyelonephritis	KP	Drug-susceptible infection	37	63	19
Deaths	Southern Sub-Saharan Africa	Both	All Ages	All causes	2019	Number	Urinary tract infections and pyelonephritis	KP	No infection	134	213	75
Deaths	Western Sub-Saharan Africa	Both	All Ages	All causes	2019	Number	Urinary tract infections and pyelonephritis	KP	Drug-susceptible infection	130	204	76
Deaths	Western Sub-Saharan Africa	Both	All Ages	All causes	2019	Number	Urinary tract infections and pyelonephritis	KP	No infection	477	687	302

S.Figure 2

measure	location	sex	age	cause	year	metric	infectious syndrome	pathogen	counterfactual	value (per 100,000)	upper (per 100,000)	lower (per 100,000)
Deaths	Global	Both	All Ages	All causes	2019	Rate	All infectious syndromes	KP	Drug-susceptible infection	2.490	3.520	1.680
Deaths	Global	Both	All Ages	All causes	2019	Rate	All infectious syndromes	KP	No infection	8.290	11.157	6.010
Deaths	Global	Both	All Ages	All causes	2019	Rate	Bacterial infections ofthe skin and subcutaneous systems	KP	Drug-susceptible infection	0.021	0.077	0.003
Deaths	Global	Both	All Ages	All causes	2019	Rate	Bacterial infections ofthe skin and subcutaneous systems	KP	No infection	0.069	0.256	0.010
Deaths	Global	Both	All Ages	All causes	2019	Rate	Bloodstream infections	KP	Drug-susceptible infection	0.831	1.350	0.467
Deaths	Global	Both	All Ages	All causes	2019	Rate	Bloodstream infections	KP	No infection	2.770	4.330	1.660
Deaths	Global	Both	All Ages	All causes	2019	Rate	Endocarditis and other cardiac infections	KP	Drug-susceptible infection	0.029	0.043	0.020
Deaths	Global	Both	All Ages	All causes	2019	Rate	Endocarditis and other cardiac infections	KP	No infection	0.101	0.140	0.074
Deaths	Global	Both	All Ages	All causes	2019	Rate	Infections of bones, joints, and related organs	KP	Drug-susceptible infection	0.005	0.011	0.001
Deaths	Global	Both	All Ages	All causes	2019	Rate	Infections of bones, joints, and related organs	KP	No infection	0.015	0.034	0.004
Deaths	Global	Both	All Ages	All causes	2019	Rate	LRIs and all related infections in the thorax	KP	Drug-susceptible infection	0.896	1.210	0.653
Deaths	Global	Both	All Ages	All causes	2019	Rate	LRIs and all related infections in the thorax	KP	No infection	3.000	3.730	2.390
Deaths	Global	Both	All Ages	All causes	2019	Rate	Meningitis and other bacterial CNS infections	KP	Drug-susceptible infection	0.116	0.176	0.076
Deaths	Global	Both	All Ages	All causes	2019	Rate	Meningitis and other bacterial CNS infections	KP	No infection	0.392	0.553	0.276
Deaths	Global	Both	All Ages	All causes	2019	Rate	Peritoneal and intra-abdominal infections	KP	Drug-susceptible infection	0.484	0.740	0.305
Deaths	Global	Both	All Ages	All causes	2019	Rate	Peritoneal and intra-abdominal infections	KP	No infection	1.590	2.360	1.040
Deaths	Global	Both	All Ages	All causes	2019	Rate	Urinary tract infections and pyelonephritis	KP	Drug-susceptible infection	0.108	0.161	0.071
Deaths	Global	Both	All Ages	All causes	2019	Rate	Urinary tract infections and pyelonephritis	KP	No infection	0.358	0.521	0.246
Deaths	East Asia	Both	All Ages	All causes	2019	Rate	All infectious syndromes	KP	Drug-susceptible infection	0.984	1.720	0.497
Deaths	East Asia	Both	All Ages	All causes	2019	Rate	All infectious syndromes	KP	No infection	3.580	5.550	2.150
Deaths	East Asia	Both	All Ages	All causes	2019	Rate	Bacterial infections ofthe skin and subcutaneous systems	KP	Drug-susceptible infection	0.015	0.055	0.002
Deaths	East Asia	Both	All Ages	All causes	2019	Rate	Bacterial infections ofthe skin and subcutaneous systems	KP	No infection	0.054	0.199	0.007
Deaths	East Asia	Both	All Ages	All causes	2019	Rate	Bloodstream infections	KP	Drug-susceptible infection	0.415	0.797	0.188
Deaths	East Asia	Both	All Ages	All causes	2019	Rate	Bloodstream infections	KP	No infection	1.510	2.580	0.814
Deaths	East Asia	Both	All Ages	All causes	2019	Rate	Endocarditis and other cardiac infections	KP	Drug-susceptible infection	0.012	0.023	0.006
Deaths	East Asia	Both	All Ages	All causes	2019	Rate	Endocarditis and other cardiac infections	KP	No infection	0.045	0.075	0.025
Deaths	East Asia	Both	All Ages	All causes	2019	Rate	Infections of bones, joints, and related organs	KP	Drug-susceptible infection	0.002	0.006	0.001
Deaths	East Asia	Both	All Ages	All causes	2019	Rate	Infections of bones, joints, and related organs	KP	No infection	0.008	0.020	0.002
Deaths	East Asia	Both	All Ages	All causes	2019	Rate	LRIs and all related infections in the thorax	KP	Drug-susceptible infection	0.213	0.365	0.115
Deaths	East Asia	Both	All Ages	All causes	2019	Rate	LRIs and all related infections in the thorax	KP	No infection	0.774	1.150	0.508
Deaths	East Asia	Both	All Ages	All causes	2019	Rate	Meningitis and other bacterial CNS infections	KP	Drug-susceptible infection	0.016	0.033	0.008
Deaths	East Asia	Both	All Ages	All causes	2019	Rate	Meningitis and other bacterial CNS infections	KP	No infection	0.058	0.113	0.033
Deaths	East Asia	Both	All Ages	All causes	2019	Rate	Peritoneal and intra-abdominal infections	KP	Drug-susceptible infection	0.242	0.449	0.118
Deaths	East Asia	Both	All Ages	All causes	2019	Rate	Peritoneal and intra-abdominal infections	KP	No infection	0.881	1.470	0.501
Deaths	East Asia	Both	All Ages	All causes	2019	Rate	Urinary tract infections and pyelonephritis	KP	Drug-susceptible infection	0.069	0.143	0.028
Deaths	East Asia	Both	All Ages	All causes	2019	Rate	Urinary tract infections and pyelonephritis	KP	No infection	0.249	0.473	0.114
Deaths	Southeast Asia	Both	All Ages	All causes	2019	Rate	All infectious syndromes	KP	Drug-susceptible infection	2.260	3.300	1.460
Deaths	Southeast Asia	Both	All Ages	All causes	2019	Rate	All infectious syndromes	KP	No infection	7.370	10.229	5.140
Deaths	Southeast Asia	Both	All Ages	All causes	2019	Rate	Bacterial infections ofthe skin and subcutaneous systems	KP	Drug-susceptible infection	0.025	0.091	0.004
Deaths	Southeast Asia	Both	All Ages	All causes	2019	Rate	Bacterial infections ofthe skin and subcutaneous systems	KP	No infection	0.082	0.302	0.012
Deaths	Southeast Asia	Both	All Ages	All causes	2019	Rate	Bloodstream infections	KP	Drug-susceptible infection	0.706	1.190	0.380
Deaths	Southeast Asia	Both	All Ages	All causes	2019	Rate	Bloodstream infections	KP	No infection	2.280	3.700	1.320
Deaths	Southeast Asia	Both	All Ages	All causes	2019	Rate	Endocarditis and other cardiac infections	KP	Drug-susceptible infection	0.028	0.041	0.020

Deaths	Southeast Asia	Both	All Ages	All causes	2019	Rate	Endocarditis and other cardiac infections	KP	No infection	0.093	0.124	0.070
Deaths	Southeast Asia	Both	All Ages	All causes	2019	Rate	Infections of bones, joints, and related organs	KP	Drug-susceptible infection	0.004	0.010	0.001
Deaths	Southeast Asia	Both	All Ages	All causes	2019	Rate	Infections of bones, joints, and related organs	KP	No infection	0.012	0.029	0.003
Deaths	Southeast Asia	Both	All Ages	All causes	2019	Rate	LRIs and all related infections in the thorax	KP	Drug-susceptible infection	0.726	1.000	0.511
Deaths	Southeast Asia	Both	All Ages	All causes	2019	Rate	LRIs and all related infections in the thorax	KP	No infection	2.360	3.070	1.830
Deaths	Southeast Asia	Both	All Ages	All causes	2019	Rate	Meningitis and other bacterial CNS infections	KP	Drug-susceptible infection	0.055	0.087	0.035
Deaths	Southeast Asia	Both	All Ages	All causes	2019	Rate	Meningitis and other bacterial CNS infections	KP	No infection	0.179	0.271	0.123
Deaths	Southeast Asia	Both	All Ages	All causes	2019	Rate	Peritoneal and intra-abdominal infections	KP	Drug-susceptible infection	0.608	0.931	0.368
Deaths	Southeast Asia	Both	All Ages	All causes	2019	Rate	Peritoneal and intra-abdominal infections	KP	No infection	1.990	2.870	1.310
Deaths	Southeast Asia	Both	All Ages	All causes	2019	Rate	Urinary tract infections and pyelonephritis	KP	Drug-susceptible infection	0.110	0.176	0.064
Deaths	Southeast Asia	Both	All Ages	All causes	2019	Rate	Urinary tract infections and pyelonephritis	KP	No infection	0.361	0.548	0.225
Deaths	Oceania	Both	All Ages	All causes	2019	Rate	All infectious syndromes	KP	Drug-susceptible infection	2.970	4.600	1.680
Deaths	Oceania	Both	All Ages	All causes	2019	Rate	All infectious syndromes	KP	No infection	10.736	14.919	7.450
Deaths	Oceania	Both	All Ages	All causes	2019	Rate	Bacterial infections ofthe skin and subcutaneous systems	KP	Drug-susceptible infection	0.018	0.069	0.002
Deaths	Oceania	Both	All Ages	All causes	2019	Rate	Bacterial infections ofthe skin and subcutaneous systems	KP	No infection	0.065	0.270	0.007
Deaths	Oceania	Both	All Ages	All causes	2019	Rate	Bloodstream infections	KP	Drug-susceptible infection	0.874	1.540	0.439
Deaths	Oceania	Both	All Ages	All causes	2019	Rate	Bloodstream infections	KP	No infection	3.160	4.970	1.830
Deaths	Oceania	Both	All Ages	All causes	2019	Rate	Endocarditis and other cardiac infections	KP	Drug-susceptible infection	0.026	0.041	0.016
Deaths	Oceania	Both	All Ages	All causes	2019	Rate	Endocarditis and other cardiac infections	KP	No infection	0.095	0.134	0.067
Deaths	Oceania	Both	All Ages	All causes	2019	Rate	Infections of bones, joints, and related organs	KP	Drug-susceptible infection	0.004	0.010	0.001
Deaths	Oceania	Both	All Ages	All causes	2019	Rate	Infections of bones, joints, and related organs	KP	No infection	0.013	0.035	0.003
Deaths	Oceania	Both	All Ages	All causes	2019	Rate	LRIs and all related infections in the thorax	KP	Drug-susceptible infection	1.650	2.570	0.949
Deaths	Oceania	Both	All Ages	All causes	2019	Rate	LRIs and all related infections in the thorax	KP	No infection	5.970	8.260	4.270
Deaths	Oceania	Both	All Ages	All causes	2019	Rate	Meningitis and other bacterial CNS infections	KP	Drug-susceptible infection	0.130	0.227	0.068
Deaths	Oceania	Both	All Ages	All causes	2019	Rate	Meningitis and other bacterial CNS infections	KP	No infection	0.470	0.738	0.285
Deaths	Oceania	Both	All Ages	All causes	2019	Rate	Peritoneal and intra-abdominal infections	KP	Drug-susceptible infection	0.208	0.386	0.102
Deaths	Oceania	Both	All Ages	All causes	2019	Rate	Peritoneal and intra-abdominal infections	KP	No infection	0.750	1.320	0.415
Deaths	Oceania	Both	All Ages	All causes	2019	Rate	Urinary tract infections and pyelonephritis	KP	Drug-susceptible infection	0.057	0.102	0.028
Deaths	Oceania	Both	All Ages	All causes	2019	Rate	Urinary tract infections and pyelonephritis	KP	No infection	0.207	0.337	0.112
Deaths	Central Asia	Both	All Ages	All causes	2019	Rate	All infectious syndromes	KP	Drug-susceptible infection	2.180	3.410	1.280
Deaths	Central Asia	Both	All Ages	All causes	2019	Rate	All infectious syndromes	KP	No infection	8.040	11.476	5.480
Deaths	Central Asia	Both	All Ages	All causes	2019	Rate	Bacterial infections ofthe skin and subcutaneous systems	KP	Drug-susceptible infection	0.014	0.052	0.001
Deaths	Central Asia	Both	All Ages	All causes	2019	Rate	Bacterial infections ofthe skin and subcutaneous systems	KP	No infection	0.050	0.195	0.005
Deaths	Central Asia	Both	All Ages	All causes	2019	Rate	Bloodstream infections	KP	Drug-susceptible infection	0.785	1.410	0.399
Deaths	Central Asia	Both	All Ages	All causes	2019	Rate	Bloodstream infections	KP	No infection	2.900	4.860	1.620
Deaths	Central Asia	Both	All Ages	All causes	2019	Rate	Endocarditis and other cardiac infections	KP	Drug-susceptible infection	0.022	0.038	0.012
Deaths	Central Asia	Both	All Ages	All causes	2019	Rate	Endocarditis and other cardiac infections	KP	No infection	0.081	0.131	0.048
Deaths	Central Asia	Both	All Ages	All causes	2019	Rate	Infections of bones, joints, and related organs	KP	Drug-susceptible infection	0.002	0.006	0.001
Deaths	Central Asia	Both	All Ages	All causes	2019	Rate	Infections of bones, joints, and related organs	KP	No infection	0.008	0.021	0.002
Deaths	Central Asia	Both	All Ages	All causes	2019	Rate	LRIs and all related infections in the thorax	KP	Drug-susceptible infection	0.644	0.922	0.421
Deaths	Central Asia	Both	All Ages	All causes	2019	Rate	LRIs and all related infections in the thorax	KP	No infection	2.370	3.040	1.860
Deaths	Central Asia	Both	All Ages	All causes	2019	Rate	Meningitis and other bacterial CNS infections	KP	Drug-susceptible infection	0.028	0.052	0.016
Deaths	Central Asia	Both	All Ages	All causes	2019	Rate	Meningitis and other bacterial CNS infections	KP	No infection	0.104	0.183	0.066
Deaths	Central Asia	Both	All Ages	All causes	2019	Rate	Peritoneal and intra-abdominal infections	KP	Drug-susceptible infection	0.549	0.905	0.304
Deaths	Central Asia	Both	All Ages	All causes	2019	Rate	Peritoneal and intra-abdominal infections	KP	No infection	2.030	3.050	1.280
Deaths	Central Asia	Both	All Ages	All causes	2019	Rate	Urinary tract infections and pyelonephritis	KP	Drug-susceptible infection	0.136	0.215	0.081
Deaths	Central Asia	Both	All Ages	All causes	2019	Rate	Urinary tract infections and pyelonephritis	KP	No infection	0.499	0.735	0.341

Deaths	Central Europe	Both	All Ages	All causes	2019	Rate	All infectious syndromes	KP	Drug-susceptible infection	2.750	4.290	1.650
Deaths	Central Europe	Both	All Ages	All causes	2019	Rate	All infectious syndromes	KP	No infection	9.590	14.394	6.170
Deaths	Central Europe	Both	All Ages	All causes	2019	Rate	Bacterial infections ofthe skin and subcutaneous systems	KP	Drug-susceptible infection	0.027	0.103	0.003
Deaths	Central Europe	Both	All Ages	All causes	2019	Rate	Bacterial infections ofthe skin and subcutaneous systems	KP	No infection	0.094	0.349	0.012
Deaths	Central Europe	Both	All Ages	All causes	2019	Rate	Bloodstream infections	KP	Drug-susceptible infection	1.090	1.940	0.525
Deaths	Central Europe	Both	All Ages	All causes	2019	Rate	Bloodstream infections	KP	No infection	3.780	6.520	2.000
Deaths	Central Europe	Both	All Ages	All causes	2019	Rate	Endocarditis and other cardiac infections	KP	Drug-susceptible infection	0.111	0.200	0.057
Deaths	Central Europe	Both	All Ages	All causes	2019	Rate	Endocarditis and other cardiac infections	KP	No infection	0.391	0.648	0.214
Deaths	Central Europe	Both	All Ages	All causes	2019	Rate	Infections of bones, joints, and related organs	KP	Drug-susceptible infection	0.004	0.009	0.001
Deaths	Central Europe	Both	All Ages	All causes	2019	Rate	Infections of bones, joints, and related organs	KP	No infection	0.013	0.029	0.004
Deaths	Central Europe	Both	All Ages	All causes	2019	Rate	LRIs and all related infections in the thorax	KP	Drug-susceptible infection	0.528	0.748	0.364
Deaths	Central Europe	Both	All Ages	All causes	2019	Rate	LRIs and all related infections in the thorax	KP	No infection	1.840	2.470	1.390
Deaths	Central Europe	Both	All Ages	All causes	2019	Rate	Meningitis and other bacterial CNS infections	KP	Drug-susceptible infection	0.022	0.044	0.011
Deaths	Central Europe	Both	All Ages	All causes	2019	Rate	Meningitis and other bacterial CNS infections	KP	No infection	0.075	0.150	0.041
Deaths	Central Europe	Both	All Ages	All causes	2019	Rate	Peritoneal and intra-abdominal infections	KP	Drug-susceptible infection	0.760	1.170	0.455
Deaths	Central Europe	Both	All Ages	All causes	2019	Rate	Peritoneal and intra-abdominal infections	KP	No infection	2.650	3.940	1.690
Deaths	Central Europe	Both	All Ages	All causes	2019	Rate	Urinary tract infections and pyelonephritis	KP	Drug-susceptible infection	0.212	0.357	0.115
Deaths	Central Europe	Both	All Ages	All causes	2019	Rate	Urinary tract infections and pyelonephritis	KP	No infection	0.745	1.210	0.438
Deaths	Eastern Europe	Both	All Ages	All causes	2019	Rate	All infectious syndromes	KP	Drug-susceptible infection	3.550	5.270	2.280
Deaths	Eastern Europe	Both	All Ages	All causes	2019	Rate	All infectious syndromes	KP	No infection	10.696	15.454	6.970
Deaths	Eastern Europe	Both	All Ages	All causes	2019	Rate	Bacterial infections ofthe skin and subcutaneous systems	KP	Drug-susceptible infection	0.043	0.151	0.007
Deaths	Eastern Europe	Both	All Ages	All causes	2019	Rate	Bacterial infections ofthe skin and subcutaneous systems	KP	No infection	0.128	0.447	0.020
Deaths	Eastern Europe	Both	All Ages	All causes	2019	Rate	Bloodstream infections	KP	Drug-susceptible infection	1.390	2.420	0.727
Deaths	Eastern Europe	Both	All Ages	All causes	2019	Rate	Bloodstream infections	KP	No infection	4.170	7.030	2.210
Deaths	Eastern Europe	Both	All Ages	All causes	2019	Rate	Endocarditis and other cardiac infections	KP	Drug-susceptible infection	0.086	0.142	0.051
Deaths	Eastern Europe	Both	All Ages	All causes	2019	Rate	Endocarditis and other cardiac infections	KP	No infection	0.268	0.443	0.160
Deaths	Eastern Europe	Both	All Ages	All causes	2019	Rate	Infections of bones, joints, and related organs	KP	Drug-susceptible infection	0.006	0.013	0.002
Deaths	Eastern Europe	Both	All Ages	All causes	2019	Rate	Infections of bones, joints, and related organs	KP	No infection	0.017	0.041	0.005
Deaths	Eastern Europe	Both	All Ages	All causes	2019	Rate	LRIs and all related infections in the thorax	KP	Drug-susceptible infection	0.655	0.934	0.454
Deaths	Eastern Europe	Both	All Ages	All causes	2019	Rate	LRIs and all related infections in the thorax	KP	No infection	1.940	2.690	1.410
Deaths	Eastern Europe	Both	All Ages	All causes	2019	Rate	Meningitis and other bacterial CNS infections	KP	Drug-susceptible infection	0.040	0.069	0.025
Deaths	Eastern Europe	Both	All Ages	All causes	2019	Rate	Meningitis and other bacterial CNS infections	KP	No infection	0.120	0.208	0.079
Deaths	Eastern Europe	Both	All Ages	All causes	2019	Rate	Peritoneal and intra-abdominal infections	KP	Drug-susceptible infection	1.030	1.520	0.654
Deaths	Eastern Europe	Both	All Ages	All causes	2019	Rate	Peritoneal and intra-abdominal infections	KP	No infection	3.110	4.510	2.010
Deaths	Eastern Europe	Both	All Ages	All causes	2019	Rate	Urinary tract infections and pyelonephritis	KP	Drug-susceptible infection	0.312	0.479	0.199
Deaths	Eastern Europe	Both	All Ages	All causes	2019	Rate	Urinary tract infections and pyelonephritis	KP	No infection	0.937	1.400	0.618
Deaths	High-income Asia Pacific	Both	All Ages	All causes	2019	Rate	All infectious syndromes	KP	Drug-susceptible infection	0.860	1.300	0.533
Deaths	High-income Asia Pacific	Both	All Ages	All causes	2019	Rate	All infectious syndromes	KP	No infection	3.760	5.080	2.770
Deaths	High-income Asia Pacific	Both	All Ages	All causes	2019	Rate	Bacterial infections ofthe skin and subcutaneous systems	KP	Drug-susceptible infection	0.008	0.028	0.001
Deaths	High-income Asia Pacific	Both	All Ages	All causes	2019	Rate	Bacterial infections ofthe skin and subcutaneous systems	KP	No infection	0.034	0.124	0.005
Deaths	High-income Asia Pacific	Both	All Ages	All causes	2019	Rate	Bloodstream infections	KP	Drug-susceptible infection	0.254	0.441	0.131
Deaths	High-income Asia Pacific	Both	All Ages	All causes	2019	Rate	Bloodstream infections	KP	No infection	1.090	1.790	0.606
Deaths	High-income Asia Pacific	Both	All Ages	All causes	2019	Rate	Endocarditis and other cardiac infections	KP	Drug-susceptible infection	0.023	0.037	0.013
Deaths	High-income Asia Pacific	Both	All Ages	All causes	2019	Rate	Endocarditis and other cardiac infections	KP	No infection	0.105	0.146	0.061
Deaths	High-income Asia Pacific	Both	All Ages	All causes	2019	Rate	Infections of bones, joints, and related organs	KP	Drug-susceptible infection	0.001	0.002	0.000
Deaths	High-income Asia Pacific	Both	All Ages	All causes	2019	Rate	Infections of bones, joints, and related organs	KP	No infection	0.004	0.010	0.001
Deaths	High-income Asia Pacific	Both	All Ages	All causes	2019	Rate	LRIs and all related infections in the thorax	KP	Drug-susceptible infection	0.287	0.399	0.199

Deaths	High-income Asia Pacific	Both	All Ages	All causes	2019	Rate	LRIs and all related infections in the thorax	KP	No infection	1.280	1.520	1.050
Deaths	High-income Asia Pacific	Both	All Ages	All causes	2019	Rate	Meningitis and other bacterial CNS infections	KP	Drug-susceptible infection	0.004	0.008	0.002
Deaths	High-income Asia Pacific	Both	All Ages	All causes	2019	Rate	Meningitis and other bacterial CNS infections	KP	No infection	0.016	0.032	0.010
Deaths	High-income Asia Pacific	Both	All Ages	All causes	2019	Rate	Peritoneal and intra-abdominal infections	KP	Drug-susceptible infection	0.213	0.348	0.117
Deaths	High-income Asia Pacific	Both	All Ages	All causes	2019	Rate	Peritoneal and intra-abdominal infections	KP	No infection	0.921	1.390	0.570
Deaths	High-income Asia Pacific	Both	All Ages	All causes	2019	Rate	Urinary tract infections and pyelonephritis	KP	Drug-susceptible infection	0.069	0.107	0.043
Deaths	High-income Asia Pacific	Both	All Ages	All causes	2019	Rate	Urinary tract infections and pyelonephritis	KP	No infection	0.306	0.435	0.217
Deaths	Australasia	Both	All Ages	All causes	2019	Rate	All infectious syndromes	KP	Drug-susceptible infection	0.514	0.803	0.317
Deaths	Australasia	Both	All Ages	All causes	2019	Rate	All infectious syndromes	KP	No infection	2.450	3.540	1.630
Deaths	Australasia	Both	All Ages	All causes	2019	Rate	Bacterial infections ofthe skin and subcutaneous systems	KP	Drug-susceptible infection	0.009	0.033	0.001
Deaths	Australasia	Both	All Ages	All causes	2019	Rate	Bacterial infections ofthe skin and subcutaneous systems	KP	No infection	0.043	0.143	0.007
Deaths	Australasia	Both	All Ages	All causes	2019	Rate	Bloodstream infections	KP	Drug-susceptible infection	0.188	0.342	0.096
Deaths	Australasia	Both	All Ages	All causes	2019	Rate	Bloodstream infections	KP	No infection	0.894	1.540	0.475
Deaths	Australasia	Both	All Ages	All causes	2019	Rate	Endocarditis and other cardiac infections	KP	Drug-susceptible infection	0.018	0.028	0.010
Deaths	Australasia	Both	All Ages	All causes	2019	Rate	Endocarditis and other cardiac infections	KP	No infection	0.085	0.124	0.053
Deaths	Australasia	Both	All Ages	All causes	2019	Rate	Infections of bones, joints, and related organs	KP	Drug-susceptible infection	0.001	0.002	0.000
Deaths	Australasia	Both	All Ages	All causes	2019	Rate	Infections of bones, joints, and related organs	KP	No infection	0.004	0.010	0.001
Deaths	Australasia	Both	All Ages	All causes	2019	Rate	LRIs and all related infections in the thorax	KP	Drug-susceptible infection	0.104	0.154	0.069
Deaths	Australasia	Both	All Ages	All causes	2019	Rate	LRIs and all related infections in the thorax	KP	No infection	0.494	0.670	0.362
Deaths	Australasia	Both	All Ages	All causes	2019	Rate	Meningitis and other bacterial CNS infections	KP	Drug-susceptible infection	0.004	0.007	0.002
Deaths	Australasia	Both	All Ages	All causes	2019	Rate	Meningitis and other bacterial CNS infections	KP	No infection	0.017	0.033	0.009
Deaths	Australasia	Both	All Ages	All causes	2019	Rate	Peritoneal and intra-abdominal infections	KP	Drug-susceptible infection	0.133	0.214	0.077
Deaths	Australasia	Both	All Ages	All causes	2019	Rate	Peritoneal and intra-abdominal infections	KP	No infection	0.636	0.981	0.399
Deaths	Australasia	Both	All Ages	All causes	2019	Rate	Urinary tract infections and pyelonephritis	KP	Drug-susceptible infection	0.058	0.090	0.038
Deaths	Australasia	Both	All Ages	All causes	2019	Rate	Urinary tract infections and pyelonephritis	KP	No infection	0.277	0.386	0.201
Deaths	Western Europe	Both	All Ages	All causes	2019	Rate	All infectious syndromes	KP	Drug-susceptible infection	1.360	2.020	0.884
Deaths	Western Europe	Both	All Ages	All causes	2019	Rate	All infectious syndromes	KP	No infection	5.480	7.590	3.870
Deaths	Western Europe	Both	All Ages	All causes	2019	Rate	Bacterial infections ofthe skin and subcutaneous systems	KP	Drug-susceptible infection	0.018	0.062	0.003
Deaths	Western Europe	Both	All Ages	All causes	2019	Rate	Bacterial infections ofthe skin and subcutaneous systems	KP	No infection	0.074	0.253	0.011
Deaths	Western Europe	Both	All Ages	All causes	2019	Rate	Bloodstream infections	KP	Drug-susceptible infection	0.448	0.778	0.228
Deaths	Western Europe	Both	All Ages	All causes	2019	Rate	Bloodstream infections	KP	No infection	1.790	3.020	0.952
Deaths	Western Europe	Both	All Ages	All causes	2019	Rate	Endocarditis and other cardiac infections	KP	Drug-susceptible infection	0.063	0.093	0.035
Deaths	Western Europe	Both	All Ages	All causes	2019	Rate	Endocarditis and other cardiac infections	KP	No infection	0.254	0.351	0.159
Deaths	Western Europe	Both	All Ages	All causes	2019	Rate	Infections of bones, joints, and related organs	KP	Drug-susceptible infection	0.002	0.004	0.001
Deaths	Western Europe	Both	All Ages	All causes	2019	Rate	Infections of bones, joints, and related organs	KP	No infection	0.007	0.016	0.002
Deaths	Western Europe	Both	All Ages	All causes	2019	Rate	LRIs and all related infections in the thorax	KP	Drug-susceptible infection	0.293	0.398	0.210
Deaths	Western Europe	Both	All Ages	All causes	2019	Rate	LRIs and all related infections in the thorax	KP	No infection	1.190	1.480	0.967
Deaths	Western Europe	Both	All Ages	All causes	2019	Rate	Meningitis and other bacterial CNS infections	KP	Drug-susceptible infection	0.007	0.014	0.004
Deaths	Western Europe	Both	All Ages	All causes	2019	Rate	Meningitis and other bacterial CNS infections	KP	No infection	0.030	0.054	0.019
Deaths	Western Europe	Both	All Ages	All causes	2019	Rate	Peritoneal and intra-abdominal infections	KP	Drug-susceptible infection	0.376	0.568	0.226
Deaths	Western Europe	Both	All Ages	All causes	2019	Rate	Peritoneal and intra-abdominal infections	KP	No infection	1.520	2.200	1.010
Deaths	Western Europe	Both	All Ages	All causes	2019	Rate	Urinary tract infections and pyelonephritis	KP	Drug-susceptible infection	0.149	0.212	0.101
Deaths	Western Europe	Both	All Ages	All causes	2019	Rate	Urinary tract infections and pyelonephritis	KP	No infection	0.615	0.822	0.459
Deaths	Southern Latin America	Both	All Ages	All causes	2019	Rate	All infectious syndromes	KP	Drug-susceptible infection	2.910	4.190	1.970
Deaths	Southern Latin America	Both	All Ages	All causes	2019	Rate	All infectious syndromes	KP	No infection	10.130	13.329	7.630
Deaths	Southern Latin America	Both	All Ages	All causes	2019	Rate	Bacterial infections ofthe skin and subcutaneous systems	KP	Drug-susceptible infection	0.032	0.110	0.005
Deaths	Southern Latin America	Both	All Ages	All causes	2019	Rate	Bacterial infections ofthe skin and subcutaneous systems	KP	No infection	0.113	0.366	0.019

Deaths	Southern Latin America	Both	All Ages	All causes	2019	Rate	Bloodstream infections	KP	Drug-susceptible infection	0.784	1.390	0.397
Deaths	Southern Latin America	Both	All Ages	All causes	2019	Rate	Bloodstream infections	KP	No infection	2.730	4.610	1.480
Deaths	Southern Latin America	Both	All Ages	All causes	2019	Rate	Endocarditis and other cardiac infections	KP	Drug-susceptible infection	0.082	0.114	0.056
Deaths	Southern Latin America	Both	All Ages	All causes	2019	Rate	Endocarditis and other cardiac infections	KP	No infection	0.285	0.356	0.224
Deaths	Southern Latin America	Both	All Ages	All causes	2019	Rate	Infections of bones, joints, and related organs	KP	Drug-susceptible infection	0.003	0.007	0.001
Deaths	Southern Latin America	Both	All Ages	All causes	2019	Rate	Infections of bones, joints, and related organs	KP	No infection	0.010	0.024	0.003
Deaths	Southern Latin America	Both	All Ages	All causes	2019	Rate	LRIs and all related infections in the thorax	KP	Drug-susceptible infection	1.090	1.440	0.798
Deaths	Southern Latin America	Both	All Ages	All causes	2019	Rate	LRIs and all related infections in the thorax	KP	No infection	3.760	4.420	3.190
Deaths	Southern Latin America	Both	All Ages	All causes	2019	Rate	Meningitis and other bacterial CNS infections	KP	Drug-susceptible infection	0.025	0.040	0.017
Deaths	Southern Latin America	Both	All Ages	All causes	2019	Rate	Meningitis and other bacterial CNS infections	KP	No infection	0.088	0.133	0.065
Deaths	Southern Latin America	Both	All Ages	All causes	2019	Rate	Peritoneal and intra-abdominal infections	KP	Drug-susceptible infection	0.622	0.939	0.386
Deaths	Southern Latin America	Both	All Ages	All causes	2019	Rate	Peritoneal and intra-abdominal infections	KP	No infection	2.170	3.140	1.480
Deaths	Southern Latin America	Both	All Ages	All causes	2019	Rate	Urinary tract infections and pyelonephritis	KP	Drug-susceptible infection	0.277	0.391	0.182
Deaths	Southern Latin America	Both	All Ages	All causes	2019	Rate	Urinary tract infections and pyelonephritis	KP	No infection	0.966	1.250	0.708
Deaths	High-income North America	Both	All Ages	All causes	2019	Rate	All infectious syndromes	KP	Drug-susceptible infection	0.768	1.190	0.490
Deaths	High-income North America	Both	All Ages	All causes	2019	Rate	All infectious syndromes	KP	No infection	3.190	4.540	2.220
Deaths	High-income North America	Both	All Ages	All causes	2019	Rate	Bacterial infections ofthe skin and subcutaneous systems	KP	Drug-susceptible infection	0.009	0.031	0.002
Deaths	High-income North America	Both	All Ages	All causes	2019	Rate	Bacterial infections ofthe skin and subcutaneous systems	KP	No infection	0.040	0.128	0.007
Deaths	High-income North America	Both	All Ages	All causes	2019	Rate	Bloodstream infections	KP	Drug-susceptible infection	0.261	0.461	0.133
Deaths	High-income North America	Both	All Ages	All causes	2019	Rate	Bloodstream infections	KP	No infection	1.090	1.840	0.587
Deaths	High-income North America	Both	All Ages	All causes	2019	Rate	Endocarditis and other cardiac infections	KP	Drug-susceptible infection	0.029	0.042	0.016
Deaths	High-income North America	Both	All Ages	All causes	2019	Rate	Endocarditis and other cardiac infections	KP	No infection	0.119	0.157	0.073
Deaths	High-income North America	Both	All Ages	All causes	2019	Rate	Infections of bones, joints, and related organs	KP	Drug-susceptible infection	0.001	0.003	0.000
Deaths	High-income North America	Both	All Ages	All causes	2019	Rate	Infections of bones, joints, and related organs	KP	No infection	0.005	0.012	0.002
Deaths	High-income North America	Both	All Ages	All causes	2019	Rate	LRIs and all related infections in the thorax	KP	Drug-susceptible infection	0.175	0.250	0.118
Deaths	High-income North America	Both	All Ages	All causes	2019	Rate	LRIs and all related infections in the thorax	KP	No infection	0.726	0.976	0.544
Deaths	High-income North America	Both	All Ages	All causes	2019	Rate	Meningitis and other bacterial CNS infections	KP	Drug-susceptible infection	0.005	0.009	0.003
Deaths	High-income North America	Both	All Ages	All causes	2019	Rate	Meningitis and other bacterial CNS infections	KP	No infection	0.021	0.038	0.013
Deaths	High-income North America	Both	All Ages	All causes	2019	Rate	Peritoneal and intra-abdominal infections	KP	Drug-susceptible infection	0.202	0.309	0.121
Deaths	High-income North America	Both	All Ages	All causes	2019	Rate	Peritoneal and intra-abdominal infections	KP	No infection	0.842	1.230	0.558
Deaths	High-income North America	Both	All Ages	All causes	2019	Rate	Urinary tract infections and pyelonephritis	KP	Drug-susceptible infection	0.086	0.123	0.059
Deaths	High-income North America	Both	All Ages	All causes	2019	Rate	Urinary tract infections and pyelonephritis	KP	No infection	0.356	0.478	0.273
Deaths	Caribbean	Both	All Ages	All causes	2019	Rate	All infectious syndromes	KP	Drug-susceptible infection	2.490	3.870	1.490
Deaths	Caribbean	Both	All Ages	All causes	2019	Rate	All infectious syndromes	KP	No infection	9.490	13.423	6.560
Deaths	Caribbean	Both	All Ages	All causes	2019	Rate	Bacterial infections ofthe skin and subcutaneous systems	KP	Drug-susceptible infection	0.035	0.125	0.006
Deaths	Caribbean	Both	All Ages	All causes	2019	Rate	Bacterial infections ofthe skin and subcutaneous systems	KP	No infection	0.133	0.439	0.021
Deaths	Caribbean	Both	All Ages	All causes	2019	Rate	Bloodstream infections	KP	Drug-susceptible infection	0.838	1.440	0.438
Deaths	Caribbean	Both	All Ages	All causes	2019	Rate	Bloodstream infections	KP	No infection	3.220	5.040	1.860
Deaths	Caribbean	Both	All Ages	All causes	2019	Rate	Endocarditis and other cardiac infections	KP	Drug-susceptible infection	0.027	0.039	0.017
Deaths	Caribbean	Both	All Ages	All causes	2019	Rate	Endocarditis and other cardiac infections	KP	No infection	0.102	0.133	0.077
Deaths	Caribbean	Both	All Ages	All causes	2019	Rate	Infections of bones, joints, and related organs	KP	Drug-susceptible infection	0.005	0.012	0.001
Deaths	Caribbean	Both	All Ages	All causes	2019	Rate	Infections of bones, joints, and related organs	KP	No infection	0.017	0.042	0.005
Deaths	Caribbean	Both	All Ages	All causes	2019	Rate	LRIs and all related infections in the thorax	KP	Drug-susceptible infection	0.839	1.240	0.537
Deaths	Caribbean	Both	All Ages	All causes	2019	Rate	LRIs and all related infections in the thorax	KP	No infection	3.200	4.190	2.390
Deaths	Caribbean	Both	All Ages	All causes	2019	Rate	Meningitis and other bacterial CNS infections	KP	Drug-susceptible infection	0.128	0.223	0.068
Deaths	Caribbean	Both	All Ages	All causes	2019	Rate	Meningitis and other bacterial CNS infections	KP	No infection	0.488	0.792	0.298
Deaths	Caribbean	Both	All Ages	All causes	2019	Rate	Peritoneal and intra-abdominal infections	KP	Drug-susceptible infection	0.512	0.852	0.285

Deaths	Caribbean	Both	All Ages	All causes	2019	Rate	Peritoneal and intra-abdominal infections	KP	No infection	1.950	2.980	1.230
Deaths	Caribbean	Both	All Ages	All causes	2019	Rate	Urinary tract infections and pyelonephritis	KP	Drug-susceptible infection	0.104	0.168	0.062
Deaths	Caribbean	Both	All Ages	All causes	2019	Rate	Urinary tract infections and pyelonephritis	KP	No infection	0.388	0.550	0.269
Deaths	Andean Latin America	Both	All Ages	All causes	2019	Rate	All infectious syndromes	KP	Drug-susceptible infection	2.760	4.070	1.780
Deaths	Andean Latin America	Both	All Ages	All causes	2019	Rate	All infectious syndromes	KP	No infection	10.188	13.945	7.120
Deaths	Andean Latin America	Both	All Ages	All causes	2019	Rate	Bacterial infections ofthe skin and subcutaneous systems	KP	Drug-susceptible infection	0.014	0.052	0.002
Deaths	Andean Latin America	Both	All Ages	All causes	2019	Rate	Bacterial infections ofthe skin and subcutaneous systems	KP	No infection	0.051	0.192	0.006
Deaths	Andean Latin America	Both	All Ages	All causes	2019	Rate	Bloodstream infections	KP	Drug-susceptible infection	0.875	1.450	0.481
Deaths	Andean Latin America	Both	All Ages	All causes	2019	Rate	Bloodstream infections	KP	No infection	3.210	5.010	1.860
Deaths	Andean Latin America	Both	All Ages	All causes	2019	Rate	Endocarditis and other cardiac infections	KP	Drug-susceptible infection	0.020	0.030	0.013
Deaths	Andean Latin America	Both	All Ages	All causes	2019	Rate	Endocarditis and other cardiac infections	KP	No infection	0.075	0.100	0.056
Deaths	Andean Latin America	Both	All Ages	All causes	2019	Rate	Infections of bones, joints, and related organs	KP	Drug-susceptible infection	0.003	0.008	0.001
Deaths	Andean Latin America	Both	All Ages	All causes	2019	Rate	Infections of bones, joints, and related organs	KP	No infection	0.011	0.027	0.003
Deaths	Andean Latin America	Both	All Ages	All causes	2019	Rate	LRIs and all related infections in the thorax	KP	Drug-susceptible infection	1.100	1.570	0.729
Deaths	Andean Latin America	Both	All Ages	All causes	2019	Rate	LRIs and all related infections in the thorax	KP	No infection	4.060	5.200	3.150
Deaths	Andean Latin America	Both	All Ages	All causes	2019	Rate	Meningitis and other bacterial CNS infections	KP	Drug-susceptible infection	0.024	0.045	0.014
Deaths	Andean Latin America	Both	All Ages	All causes	2019	Rate	Meningitis and other bacterial CNS infections	KP	No infection	0.089	0.153	0.056
Deaths	Andean Latin America	Both	All Ages	All causes	2019	Rate	Peritoneal and intra-abdominal infections	KP	Drug-susceptible infection	0.612	0.954	0.363
Deaths	Andean Latin America	Both	All Ages	All causes	2019	Rate	Peritoneal and intra-abdominal infections	KP	No infection	2.240	3.330	1.440
Deaths	Andean Latin America	Both	All Ages	All causes	2019	Rate	Urinary tract infections and pyelonephritis	KP	Drug-susceptible infection	0.121	0.190	0.073
Deaths	Andean Latin America	Both	All Ages	All causes	2019	Rate	Urinary tract infections and pyelonephritis	KP	No infection	0.448	0.642	0.298
Deaths	Central Latin America	Both	All Ages	All causes	2019	Rate	All infectious syndromes	KP	Drug-susceptible infection	1.870	2.870	1.130
Deaths	Central Latin America	Both	All Ages	All causes	2019	Rate	All infectious syndromes	KP	No infection	6.960	9.840	4.720
Deaths	Central Latin America	Both	All Ages	All causes	2019	Rate	Bacterial infections ofthe skin and subcutaneous systems	KP	Drug-susceptible infection	0.020	0.069	0.003
Deaths	Central Latin America	Both	All Ages	All causes	2019	Rate	Bacterial infections ofthe skin and subcutaneous systems	KP	No infection	0.074	0.263	0.012
Deaths	Central Latin America	Both	All Ages	All causes	2019	Rate	Bloodstream infections	KP	Drug-susceptible infection	0.671	1.150	0.348
Deaths	Central Latin America	Both	All Ages	All causes	2019	Rate	Bloodstream infections	KP	No infection	2.500	4.020	1.380
Deaths	Central Latin America	Both	All Ages	All causes	2019	Rate	Endocarditis and other cardiac infections	KP	Drug-susceptible infection	0.019	0.028	0.012
Deaths	Central Latin America	Both	All Ages	All causes	2019	Rate	Endocarditis and other cardiac infections	KP	No infection	0.068	0.094	0.049
Deaths	Central Latin America	Both	All Ages	All causes	2019	Rate	Infections of bones, joints, and related organs	KP	Drug-susceptible infection	0.003	0.007	0.001
Deaths	Central Latin America	Both	All Ages	All causes	2019	Rate	Infections of bones, joints, and related organs	KP	No infection	0.011	0.026	0.003
Deaths	Central Latin America	Both	All Ages	All causes	2019	Rate	LRIs and all related infections in the thorax	KP	Drug-susceptible infection	0.471	0.699	0.308
Deaths	Central Latin America	Both	All Ages	All causes	2019	Rate	LRIs and all related infections in the thorax	KP	No infection	1.760	2.390	1.290
Deaths	Central Latin America	Both	All Ages	All causes	2019	Rate	Meningitis and other bacterial CNS infections	KP	Drug-susceptible infection	0.020	0.034	0.012
Deaths	Central Latin America	Both	All Ages	All causes	2019	Rate	Meningitis and other bacterial CNS infections	KP	No infection	0.073	0.121	0.049
Deaths	Central Latin America	Both	All Ages	All causes	2019	Rate	Peritoneal and intra-abdominal infections	KP	Drug-susceptible infection	0.543	0.852	0.325
Deaths	Central Latin America	Both	All Ages	All causes	2019	Rate	Peritoneal and intra-abdominal infections	KP	No infection	2.020	2.920	1.340
Deaths	Central Latin America	Both	All Ages	All causes	2019	Rate	Urinary tract infections and pyelonephritis	KP	Drug-susceptible infection	0.124	0.197	0.076
Deaths	Central Latin America	Both	All Ages	All causes	2019	Rate	Urinary tract infections and pyelonephritis	KP	No infection	0.458	0.660	0.313
Deaths	Tropical Latin America	Both	All Ages	All causes	2019	Rate	All infectious syndromes	KP	Drug-susceptible infection	2.640	3.690	1.840
Deaths	Tropical Latin America	Both	All Ages	All causes	2019	Rate	All infectious syndromes	KP	No infection	8.860	11.819	6.500
Deaths	Tropical Latin America	Both	All Ages	All causes	2019	Rate	Bacterial infections ofthe skin and subcutaneous systems	KP	Drug-susceptible infection	0.029	0.099	0.005
Deaths	Tropical Latin America	Both	All Ages	All causes	2019	Rate	Bacterial infections ofthe skin and subcutaneous systems	KP	No infection	0.096	0.324	0.017
Deaths	Tropical Latin America	Both	All Ages	All causes	2019	Rate	Bloodstream infections	KP	Drug-susceptible infection	0.827	1.360	0.449
Deaths	Tropical Latin America	Both	All Ages	All causes	2019	Rate	Bloodstream infections	KP	No infection	2.780	4.540	1.600
Deaths	Tropical Latin America	Both	All Ages	All causes	2019	Rate	Endocarditis and other cardiac infections	KP	Drug-susceptible infection	0.046	0.062	0.032
Deaths	Tropical Latin America	Both	All Ages	All causes	2019	Rate	Endocarditis and other cardiac infections	KP	No infection	0.155	0.195	0.116

Deaths	Tropical Latin America	Both	All Ages	All causes	2019	Rate	Infections of bones, joints, and related organs	KP	Drug-susceptible infection	0.004	0.009	0.001
Deaths	Tropical Latin America	Both	All Ages	All causes	2019	Rate	Infections of bones, joints, and related organs	KP	No infection	0.012	0.029	0.003
Deaths	Tropical Latin America	Both	All Ages	All causes	2019	Rate	LRIs and all related infections in the thorax	KP	Drug-susceptible infection	0.914	1.200	0.676
Deaths	Tropical Latin America	Both	All Ages	All causes	2019	Rate	LRIs and all related infections in the thorax	KP	No infection	3.070	3.820	2.490
Deaths	Tropical Latin America	Both	All Ages	All causes	2019	Rate	Meningitis and other bacterial CNS infections	KP	Drug-susceptible infection	0.028	0.043	0.019
Deaths	Tropical Latin America	Both	All Ages	All causes	2019	Rate	Meningitis and other bacterial CNS infections	KP	No infection	0.094	0.141	0.066
Deaths	Tropical Latin America	Both	All Ages	All causes	2019	Rate	Peritoneal and intra-abdominal infections	KP	Drug-susceptible infection	0.543	0.802	0.351
Deaths	Tropical Latin America	Both	All Ages	All causes	2019	Rate	Peritoneal and intra-abdominal infections	KP	No infection	1.820	2.610	1.250
Deaths	Tropical Latin America	Both	All Ages	All causes	2019	Rate	Urinary tract infections and pyelonephritis	KP	Drug-susceptible infection	0.248	0.328	0.171
Deaths	Tropical Latin America	Both	All Ages	All causes	2019	Rate	Urinary tract infections and pyelonephritis	KP	No infection	0.834	1.050	0.610
Deaths	North Africa and Middle East	Both	All Ages	All causes	2019	Rate	All infectious syndromes	KP	Drug-susceptible infection	1.860	2.780	1.170
Deaths	North Africa and Middle East	Both	All Ages	All causes	2019	Rate	All infectious syndromes	KP	No infection	6.100	8.770	4.120
Deaths	North Africa and Middle East	Both	All Ages	All causes	2019	Rate	Bacterial infections ofthe skin and subcutaneous systems	KP	Drug-susceptible infection	0.015	0.056	0.002
Deaths	North Africa and Middle East	Both	All Ages	All causes	2019	Rate	Bacterial infections ofthe skin and subcutaneous systems	KP	No infection	0.048	0.186	0.006
Deaths	North Africa and Middle East	Both	All Ages	All causes	2019	Rate	Bloodstream infections	KP	Drug-susceptible infection	0.660	1.110	0.353
Deaths	North Africa and Middle East	Both	All Ages	All causes	2019	Rate	Bloodstream infections	KP	No infection	2.200	3.500	1.250
Deaths	North Africa and Middle East	Both	All Ages	All causes	2019	Rate	Endocarditis and other cardiac infections	KP	Drug-susceptible infection	0.025	0.037	0.016
Deaths	North Africa and Middle East	Both	All Ages	All causes	2019	Rate	Endocarditis and other cardiac infections	KP	No infection	0.081	0.116	0.056
Deaths	North Africa and Middle East	Both	All Ages	All causes	2019	Rate	Infections of bones, joints, and related organs	KP	Drug-susceptible infection	0.005	0.013	0.001
Deaths	North Africa and Middle East	Both	All Ages	All causes	2019	Rate	Infections of bones, joints, and related organs	KP	No infection	0.017	0.040	0.004
Deaths	North Africa and Middle East	Both	All Ages	All causes	2019	Rate	LRIs and all related infections in the thorax	KP	Drug-susceptible infection	0.579	0.809	0.388
Deaths	North Africa and Middle East	Both	All Ages	All causes	2019	Rate	LRIs and all related infections in the thorax	KP	No infection	1.950	2.530	1.480
Deaths	North Africa and Middle East	Both	All Ages	All causes	2019	Rate	Meningitis and other bacterial CNS infections	KP	Drug-susceptible infection	0.059	0.111	0.032
Deaths	North Africa and Middle East	Both	All Ages	All causes	2019	Rate	Meningitis and other bacterial CNS infections	KP	No infection	0.202	0.368	0.117
Deaths	North Africa and Middle East	Both	All Ages	All causes	2019	Rate	Peritoneal and intra-abdominal infections	KP	Drug-susceptible infection	0.454	0.709	0.268
Deaths	North Africa and Middle East	Both	All Ages	All causes	2019	Rate	Peritoneal and intra-abdominal infections	KP	No infection	1.400	2.130	0.857
Deaths	North Africa and Middle East	Both	All Ages	All causes	2019	Rate	Urinary tract infections and pyelonephritis	KP	Drug-susceptible infection	0.062	0.113	0.030
Deaths	North Africa and Middle East	Both	All Ages	All causes	2019	Rate	Urinary tract infections and pyelonephritis	KP	No infection	0.201	0.346	0.100
Deaths	South Asia	Both	All Ages	All causes	2019	Rate	All infectious syndromes	KP	Drug-susceptible infection	3.420	4.870	2.370
Deaths	South Asia	Both	All Ages	All causes	2019	Rate	All infectious syndromes	KP	No infection	9.710	13.353	6.880
Deaths	South Asia	Both	All Ages	All causes	2019	Rate	Bacterial infections ofthe skin and subcutaneous systems	KP	Drug-susceptible infection	0.028	0.112	0.003
Deaths	South Asia	Both	All Ages	All causes	2019	Rate	Bacterial infections ofthe skin and subcutaneous systems	KP	No infection	0.078	0.308	0.008
Deaths	South Asia	Both	All Ages	All causes	2019	Rate	Bloodstream infections	KP	Drug-susceptible infection	1.150	1.850	0.647
Deaths	South Asia	Both	All Ages	All causes	2019	Rate	Bloodstream infections	KP	No infection	3.300	5.300	1.890
Deaths	South Asia	Both	All Ages	All causes	2019	Rate	Endocarditis and other cardiac infections	KP	Drug-susceptible infection	0.030	0.043	0.021
Deaths	South Asia	Both	All Ages	All causes	2019	Rate	Endocarditis and other cardiac infections	KP	No infection	0.085	0.120	0.060
Deaths	South Asia	Both	All Ages	All causes	2019	Rate	Infections of bones, joints, and related organs	KP	Drug-susceptible infection	0.008	0.020	0.002
Deaths	South Asia	Both	All Ages	All causes	2019	Rate	Infections of bones, joints, and related organs	KP	No infection	0.023	0.054	0.006
Deaths	South Asia	Both	All Ages	All causes	2019	Rate	LRIs and all related infections in the thorax	KP	Drug-susceptible infection	1.270	1.670	0.944
Deaths	South Asia	Both	All Ages	All causes	2019	Rate	LRIs and all related infections in the thorax	KP	No infection	3.600	4.650	2.750
Deaths	South Asia	Both	All Ages	All causes	2019	Rate	Meningitis and other bacterial CNS infections	KP	Drug-susceptible infection	0.140	0.203	0.097
Deaths	South Asia	Both	All Ages	All causes	2019	Rate	Meningitis and other bacterial CNS infections	KP	No infection	0.397	0.570	0.284
Deaths	South Asia	Both	All Ages	All causes	2019	Rate	Peritoneal and intra-abdominal infections	KP	Drug-susceptible infection	0.654	1.020	0.402
Deaths	South Asia	Both	All Ages	All causes	2019	Rate	Peritoneal and intra-abdominal infections	KP	No infection	1.830	2.770	1.150
Deaths	South Asia	Both	All Ages	All causes	2019	Rate	Urinary tract infections and pyelonephritis	KP	Drug-susceptible infection	0.142	0.200	0.098
Deaths	South Asia	Both	All Ages	All causes	2019	Rate	Urinary tract infections and pyelonephritis	KP	No infection	0.395	0.547	0.283
Deaths	Central Sub-Saharan Africa	Both	All Ages	All causes	2019	Rate	All infectious syndromes	KP	Drug-susceptible infection	3.690	5.430	2.300

Deaths	Central Sub-Saharan Africa	Both	All Ages	All causes	2019	Rate	All infectious syndromes	KP	No infection	14.177	18.627	10.735
Deaths	Central Sub-Saharan Africa	Both	All Ages	All causes	2019	Rate	Bacterial infections ofthe skin and subcutaneous systems	KP	Drug-susceptible infection	0.016	0.070	0.001
Deaths	Central Sub-Saharan Africa	Both	All Ages	All causes	2019	Rate	Bacterial infections ofthe skin and subcutaneous systems	KP	No infection	0.063	0.266	0.005
Deaths	Central Sub-Saharan Africa	Both	All Ages	All causes	2019	Rate	Bloodstream infections	KP	Drug-susceptible infection	1.030	1.690	0.575
Deaths	Central Sub-Saharan Africa	Both	All Ages	All causes	2019	Rate	Bloodstream infections	KP	No infection	3.970	5.810	2.620
Deaths	Central Sub-Saharan Africa	Both	All Ages	All causes	2019	Rate	Endocarditis and other cardiac infections	KP	Drug-susceptible infection	0.018	0.031	0.010
Deaths	Central Sub-Saharan Africa	Both	All Ages	All causes	2019	Rate	Endocarditis and other cardiac infections	KP	No infection	0.071	0.107	0.046
Deaths	Central Sub-Saharan Africa	Both	All Ages	All causes	2019	Rate	Infections of bones, joints, and related organs	KP	Drug-susceptible infection	0.005	0.014	0.001
Deaths	Central Sub-Saharan Africa	Both	All Ages	All causes	2019	Rate	Infections of bones, joints, and related organs	KP	No infection	0.020	0.050	0.005
Deaths	Central Sub-Saharan Africa	Both	All Ages	All causes	2019	Rate	LRIs and all related infections in the thorax	KP	Drug-susceptible infection	1.940	2.840	1.230
Deaths	Central Sub-Saharan Africa	Both	All Ages	All causes	2019	Rate	LRIs and all related infections in the thorax	KP	No infection	7.480	9.630	5.740
Deaths	Central Sub-Saharan Africa	Both	All Ages	All causes	2019	Rate	Meningitis and other bacterial CNS infections	KP	Drug-susceptible infection	0.218	0.351	0.125
Deaths	Central Sub-Saharan Africa	Both	All Ages	All causes	2019	Rate	Meningitis and other bacterial CNS infections	KP	No infection	0.841	1.240	0.564
Deaths	Central Sub-Saharan Africa	Both	All Ages	All causes	2019	Rate	Peritoneal and intra-abdominal infections	KP	Drug-susceptible infection	0.425	0.727	0.222
Deaths	Central Sub-Saharan Africa	Both	All Ages	All causes	2019	Rate	Peritoneal and intra-abdominal infections	KP	No infection	1.630	2.630	0.977
Deaths	Central Sub-Saharan Africa	Both	All Ages	All causes	2019	Rate	Urinary tract infections and pyelonephritis	KP	Drug-susceptible infection	0.025	0.042	0.013
Deaths	Central Sub-Saharan Africa	Both	All Ages	All causes	2019	Rate	Urinary tract infections and pyelonephritis	KP	No infection	0.097	0.148	0.056
Deaths	Eastern Sub-Saharan Africa	Both	All Ages	All causes	2019	Rate	All infectious syndromes	KP	Drug-susceptible infection	4.170	5.960	2.730
Deaths	Eastern Sub-Saharan Africa	Both	All Ages	All causes	2019	Rate	All infectious syndromes	KP	No infection	15.420	19.831	11.856
Deaths	Eastern Sub-Saharan Africa	Both	All Ages	All causes	2019	Rate	Bacterial infections ofthe skin and subcutaneous systems	KP	Drug-susceptible infection	0.018	0.073	0.002
Deaths	Eastern Sub-Saharan Africa	Both	All Ages	All causes	2019	Rate	Bacterial infections ofthe skin and subcutaneous systems	KP	No infection	0.065	0.263	0.006
Deaths	Eastern Sub-Saharan Africa	Both	All Ages	All causes	2019	Rate	Bloodstream infections	KP	Drug-susceptible infection	1.370	2.140	0.830
Deaths	Eastern Sub-Saharan Africa	Both	All Ages	All causes	2019	Rate	Bloodstream infections	KP	No infection	5.070	7.350	3.360
Deaths	Eastern Sub-Saharan Africa	Both	All Ages	All causes	2019	Rate	Endocarditis and other cardiac infections	KP	Drug-susceptible infection	0.019	0.032	0.011
Deaths	Eastern Sub-Saharan Africa	Both	All Ages	All causes	2019	Rate	Endocarditis and other cardiac infections	KP	No infection	0.071	0.106	0.048
Deaths	Eastern Sub-Saharan Africa	Both	All Ages	All causes	2019	Rate	Infections of bones, joints, and related organs	KP	Drug-susceptible infection	0.006	0.014	0.002
Deaths	Eastern Sub-Saharan Africa	Both	All Ages	All causes	2019	Rate	Infections of bones, joints, and related organs	KP	No infection	0.020	0.046	0.006
Deaths	Eastern Sub-Saharan Africa	Both	All Ages	All causes	2019	Rate	LRIs and all related infections in the thorax	KP	Drug-susceptible infection	1.880	2.660	1.270
Deaths	Eastern Sub-Saharan Africa	Both	All Ages	All causes	2019	Rate	LRIs and all related infections in the thorax	KP	No infection	7.010	8.790	5.570
Deaths	Eastern Sub-Saharan Africa	Both	All Ages	All causes	2019	Rate	Meningitis and other bacterial CNS infections	KP	Drug-susceptible infection	0.336	0.513	0.206
Deaths	Eastern Sub-Saharan Africa	Both	All Ages	All causes	2019	Rate	Meningitis and other bacterial CNS infections	KP	No infection	1.220	1.690	0.873
Deaths	Eastern Sub-Saharan Africa	Both	All Ages	All causes	2019	Rate	Peritoneal and intra-abdominal infections	KP	Drug-susceptible infection	0.509	0.823	0.287
Deaths	Eastern Sub-Saharan Africa	Both	All Ages	All causes	2019	Rate	Peritoneal and intra-abdominal infections	KP	No infection	1.860	2.770	1.190
Deaths	Eastern Sub-Saharan Africa	Both	All Ages	All causes	2019	Rate	Urinary tract infections and pyelonephritis	KP	Drug-susceptible infection	0.029	0.046	0.016
Deaths	Eastern Sub-Saharan Africa	Both	All Ages	All causes	2019	Rate	Urinary tract infections and pyelonephritis	KP	No infection	0.105	0.156	0.057
Deaths	Southern Sub-Saharan Africa	Both	All Ages	All causes	2019	Rate	All infectious syndromes	KP	Drug-susceptible infection	3.770	5.540	2.400
Deaths	Southern Sub-Saharan Africa	Both	All Ages	All causes	2019	Rate	All infectious syndromes	KP	No infection	13.736	17.766	10.505
Deaths	Southern Sub-Saharan Africa	Both	All Ages	All causes	2019	Rate	Bacterial infections ofthe skin and subcutaneous systems	KP	Drug-susceptible infection	0.029	0.113	0.004
Deaths	Southern Sub-Saharan Africa	Both	All Ages	All causes	2019	Rate	Bacterial infections ofthe skin and subcutaneous systems	KP	No infection	0.106	0.407	0.012
Deaths	Southern Sub-Saharan Africa	Both	All Ages	All causes	2019	Rate	Bloodstream infections	KP	Drug-susceptible infection	1.320	2.110	0.762
Deaths	Southern Sub-Saharan Africa	Both	All Ages	All causes	2019	Rate	Bloodstream infections	KP	No infection	4.770	7.030	3.110
Deaths	Southern Sub-Saharan Africa	Both	All Ages	All causes	2019	Rate	Endocarditis and other cardiac infections	KP	Drug-susceptible infection	0.032	0.048	0.020
Deaths	Southern Sub-Saharan Africa	Both	All Ages	All causes	2019	Rate	Endocarditis and other cardiac infections	KP	No infection	0.115	0.160	0.084
Deaths	Southern Sub-Saharan Africa	Both	All Ages	All causes	2019	Rate	Infections of bones, joints, and related organs	KP	Drug-susceptible infection	0.006	0.016	0.002
Deaths	Southern Sub-Saharan Africa	Both	All Ages	All causes	2019	Rate	Infections of bones, joints, and related organs	KP	No infection	0.022	0.051	0.006
Deaths	Southern Sub-Saharan Africa	Both	All Ages	All causes	2019	Rate	LRIs and all related infections in the thorax	KP	Drug-susceptible infection	1.750	2.490	1.170
Deaths	Southern Sub-Saharan Africa	Both	All Ages	All causes	2019	Rate	LRIs and all related infections in the thorax	KP	No infection	6.410	7.850	5.260

Deaths	Southern Sub-Saharan Africa	Both	All Ages	All causes	2019	Rate	Meningitis and other bacterial CNS infections	KP	Drug-susceptible infection	0.147	0.216	0.091
Deaths	Southern Sub-Saharan Africa	Both	All Ages	All causes	2019	Rate	Meningitis and other bacterial CNS infections	KP	No infection	0.540	0.723	0.399
Deaths	Southern Sub-Saharan Africa	Both	All Ages	All causes	2019	Rate	Peritoneal and intra-abdominal infections	KP	Drug-susceptible infection	0.442	0.723	0.247
Deaths	Southern Sub-Saharan Africa	Both	All Ages	All causes	2019	Rate	Peritoneal and intra-abdominal infections	KP	No infection	1.600	2.420	1.040
Deaths	Southern Sub-Saharan Africa	Both	All Ages	All causes	2019	Rate	Urinary tract infections and pyelonephritis	KP	Drug-susceptible infection	0.047	0.080	0.024
Deaths	Southern Sub-Saharan Africa	Both	All Ages	All causes	2019	Rate	Urinary tract infections and pyelonephritis	KP	No infection	0.170	0.271	0.095
Deaths	Western Sub-Saharan Africa	Both	All Ages	All causes	2019	Rate	All infectious syndromes	KP	Drug-susceptible infection	5.650	8.080	3.790
Deaths	Western Sub-Saharan Africa	Both	All Ages	All causes	2019	Rate	All infectious syndromes	KP	No infection	20.490	26.312	15.720
Deaths	Western Sub-Saharan Africa	Both	All Ages	All causes	2019	Rate	Bacterial infections ofthe skin and subcutaneous systems	KP	Drug-susceptible infection	0.019	0.076	0.002
Deaths	Western Sub-Saharan Africa	Both	All Ages	All causes	2019	Rate	Bacterial infections ofthe skin and subcutaneous systems	KP	No infection	0.069	0.273	0.006
Deaths	Western Sub-Saharan Africa	Both	All Ages	All causes	2019	Rate	Bloodstream infections	KP	Drug-susceptible infection	1.570	2.410	0.934
Deaths	Western Sub-Saharan Africa	Both	All Ages	All causes	2019	Rate	Bloodstream infections	KP	No infection	5.700	8.260	3.760
Deaths	Western Sub-Saharan Africa	Both	All Ages	All causes	2019	Rate	Endocarditis and other cardiac infections	KP	Drug-susceptible infection	0.019	0.029	0.013
Deaths	Western Sub-Saharan Africa	Both	All Ages	All causes	2019	Rate	Endocarditis and other cardiac infections	KP	No infection	0.071	0.096	0.054
Deaths	Western Sub-Saharan Africa	Both	All Ages	All causes	2019	Rate	Infections of bones, joints, and related organs	KP	Drug-susceptible infection	0.005	0.013	0.001
Deaths	Western Sub-Saharan Africa	Both	All Ages	All causes	2019	Rate	Infections of bones, joints, and related organs	KP	No infection	0.019	0.044	0.005
Deaths	Western Sub-Saharan Africa	Both	All Ages	All causes	2019	Rate	LRIs and all related infections in the thorax	KP	Drug-susceptible infection	2.810	3.890	1.900
Deaths	Western Sub-Saharan Africa	Both	All Ages	All causes	2019	Rate	LRIs and all related infections in the thorax	KP	No infection	10.170	12.744	8.030
Deaths	Western Sub-Saharan Africa	Both	All Ages	All causes	2019	Rate	Meningitis and other bacterial CNS infections	KP	Drug-susceptible infection	0.720	1.200	0.404
Deaths	Western Sub-Saharan Africa	Both	All Ages	All causes	2019	Rate	Meningitis and other bacterial CNS infections	KP	No infection	2.590	3.900	1.680
Deaths	Western Sub-Saharan Africa	Both	All Ages	All causes	2019	Rate	Peritoneal and intra-abdominal infections	KP	Drug-susceptible infection	0.488	0.808	0.257
Deaths	Western Sub-Saharan Africa	Both	All Ages	All causes	2019	Rate	Peritoneal and intra-abdominal infections	KP	No infection	1.770	2.750	1.060
Deaths	Western Sub-Saharan Africa	Both	All Ages	All causes	2019	Rate	Urinary tract infections and pyelonephritis	KP	Drug-susceptible infection	0.029	0.045	0.017
Deaths	Western Sub-Saharan Africa	Both	All Ages	All causes	2019	Rate	Urinary tract infections and pyelonephritis	KP	No infection	0.104	0.151	0.066
